# Solvent-Dependent Chemoselectivity Switch to Arg-Lys Imidazole Cross-Links

**DOI:** 10.1021/acs.orglett.4c03101

**Published:** 2024-09-20

**Authors:** Ana Villalobos Galindo, Monika Raj

**Affiliations:** Department of Chemistry, Emory University, Atlanta, Georgia 30322, United States

## Abstract

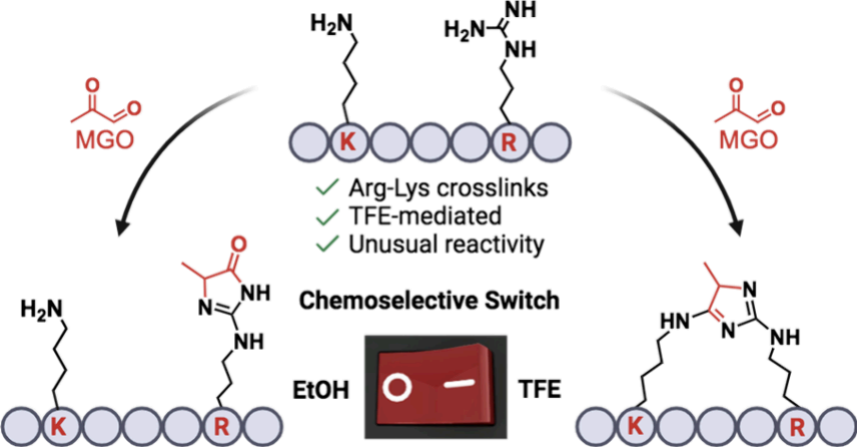

Herein, we report a trifluoroethanol-mediated, chemoselective method for the formation of Arg-Lys imidazole cross-links with methylglyoxal and its application in the selective macrocyclization of peptides between Lys and Arg and the late-stage diversification of Lys-containing peptides with guanidine. Our findings highlight the critical role of solvent choice in controlling chemoselectivity, providing valuable insights into solvent-dependent peptide modification.

## Introduction

**Figure 1:**
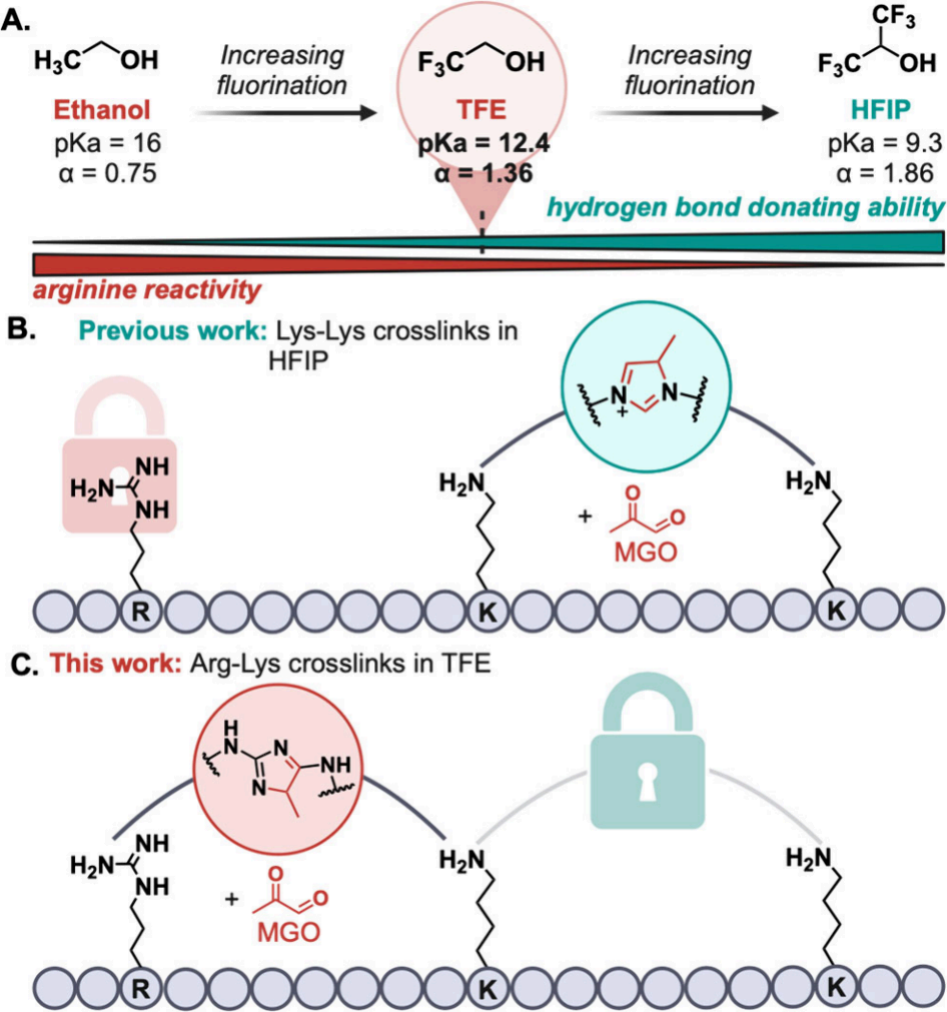
(A) Solvent-reactivity spectrum of ethanol, TFE, and HFIP. (B) HFIP-mediated Lys-Lys imidazole cross-links. (C) Chemoselectivity switch to Arg-Lys cross-links in TFE.

Solvents are well-known to affect the thermodynamics, kinetics, and selectivity of chemical reactions by impacting factors such as solubility, stability, polarity, dielectric constant, proticity, viscosity, solvent-reactant interactions, and pH.^1^ Among these, alcoholic solvents like ethanol (EtOH), trifluoroethanol (TFE), and hexafluoroisopropanol (HFIP) are frequently employed in structural biology because they stabilize the secondary structure of macromolecules, especially proteins.^2−9^ Despite their seemingly minor structural differences, these solvents exhibit distinct physical and chemical properties that affect their interactions with macromolecules.^10,11^ Specifically, the absence of electronegative fluorine atoms in EtOH, compared to the increasing fluorine content in TFE and HFIP (Figure 1A). This creates a solvent-reactivity spectrum that can be exploited to achieve diverse chemoselective patterns of amino acids (Figure 1B,C). The presence of electron-withdrawing fluorine atoms enhances the Brønsted acidity of the hydroxy proton in TFE and HFIP, resulting in greater acidity (p*K*_a_ = 12.4 for TFE and p*K*_a_ = 9.3 for HFIP) and higher hydrogen bond donating ability (α = 1.86 for HFIP and α = 1.36 for TFE) compared to the EtOH (p*K*_a_ = 16, α = 0.75) (Figure 1A).^10,12−17^ Consequently, these enhanced chemical attributes have been particularly effective in stabilizing the guanidinium group of arginine (Arg) by the formation of H-bonds.^18−20^

Recently, the Chen lab utilized the proton-shuttling properties of HFIP to achieve the chemoselective reaction of methylglyoxal (MGO) between two lysines (Lys) in the presence of Arg (Figure 1B).^21^ The high acidity and strong hydrogen bonding capacity of HFIP significantly suppress the reactivity of Arg toward MGO, thereby favoring the exclusive coupling between Lys residues. We hypothesized that partial stabilization of Arg residues using a solvent like TFE could shift the selectivity of the MGO reaction from Lys-Lys to Lys-Arg, leading to the formation of unique imidazole moieties (Figure 1C). Herein, we demonstrate a chemoselectivity shift in the MGO reaction, from two lysines to between one Lys and one Arg in the presence of TFE, producing Arg-Lys imidazole cross-link. This reaction is critically dependent on the choice of solvent, with TFE selectively promoting cyclization between Arg and Lys, in contrast to the Arg-MGO adducts formed in the presence of EtOH and Lys-Lys coupling observed in HFIP.^21^ Furthermore, we applied this TFE-mediated Arg-Lys imidazole cross-link formation for the late-stage functionalization of Lys containing peptides in nearly quantitative conversions. These results demonstrate the robustness and versatility of the solvent impact in this chemistry, demonstrating its potential for selective macrocylization and late-stage diversification of peptides.

## Results and Discussion

**Figure 2:**
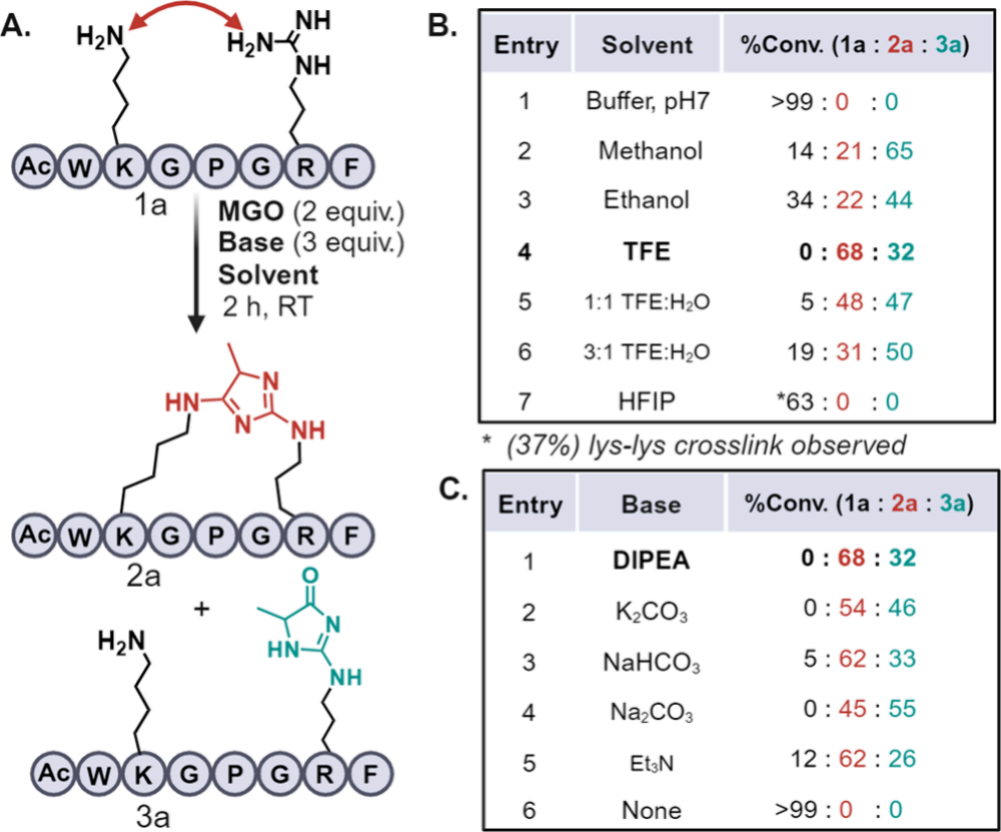
(A) Arg-Lys MGO cross-linking to generate imidazole product **2a** and Arg-MGO adduct **3a**. (B) Effect of solvents on the reaction. (C) Effect of bases on the reaction.

The high reactivity of Arg with MGO to form several adducts in aqueous or ethanolic solutions is well documented.^22,23^ Recently, Chen et al. demonstrated the ablation of Arg reactivity with MGO in HFIP.^21^ To investigate the impact of solvent variability on the selectivity of MGO reaction, we carried out reactions on a model peptide Ac-WKGPGRF (**1a**) with 2 equiv of MGO and 3 equiv of N,N-Diisopropylethylamine (DIPEA) in varying solvents (Figure 2; Supporting Information Figure S1).

No modification was observed in sodium phosphate buffer (pH 7) due to the high p*K*_a_ of Arg (Figure 2B, entry 1; Figure S1a). Next, we evaluated the reaction in nonfluorinated alcoholic solvents, specifically methanol and ethanol, which have p*K*_a_ values of 15.5 and 16, respectively (Figure S1b,c). The results showed poor conversion to the desired Arg-Lys imidazole cross-link (**2a**), with a predominant formation of the Arg-MGO adduct (**3a**) (Figure 2B, entries 2 and 3). Small amount of Lys-Lys intermolecular cross-link product was also observed under the reaction conditions. This finding underscores the increased reactivity of Arg with MGO when exposed to nonstabilizing solvents like methanol and ethanol. Additionally, the heightened reactivity of the imide nitrogen of Arg in these nonfluorinated solvents hinders the effective trapping of the Arg-MGO imine intermediate by Lys, leading to the extensive formation of the undesired Arg-MGO adduct (**3a**). To slightly mitigate the reactivity of Arg’s imide nitrogen toward MGO, we carried out the reaction of peptide **1a** in a fluorinated alcohol, trifluoroethanol (TFE), for 2 h (Figure 2B, entry 4; Figure S1d). Surprisingly, this resulted in a 68% conversion of peptide **1a** to the Lys-Arg imidazole cross-link product (**2a**), with a 32% conversion to the Arg-MGO adduct (**3a**). Next, we screened cosolvent mixtures of TFE with water and observed reduction in the formation of the desired Arg-Lys imidazole cross-link **2a** to 48% in TFE:H_2_O 1:1 mixture and to 31% in 3:1 TFE:H_2_O mixture along with an increased formation of Arg-MGO adducts (**3a**) (Figure 2B, entries 5 and 6, Figure S1e,f). These results further support our hypothesis that TFE forms strong H-bonds with imide of Arg as compared to water or ethanol thus significantly decreases the reactivity toward MGO.

As expected, no product was observed in HFIP, as the extensive hydrogen bonding interactions with Arg completely inhibited its reactivity (Figure 2B, entry 7).

**Figure 3:**
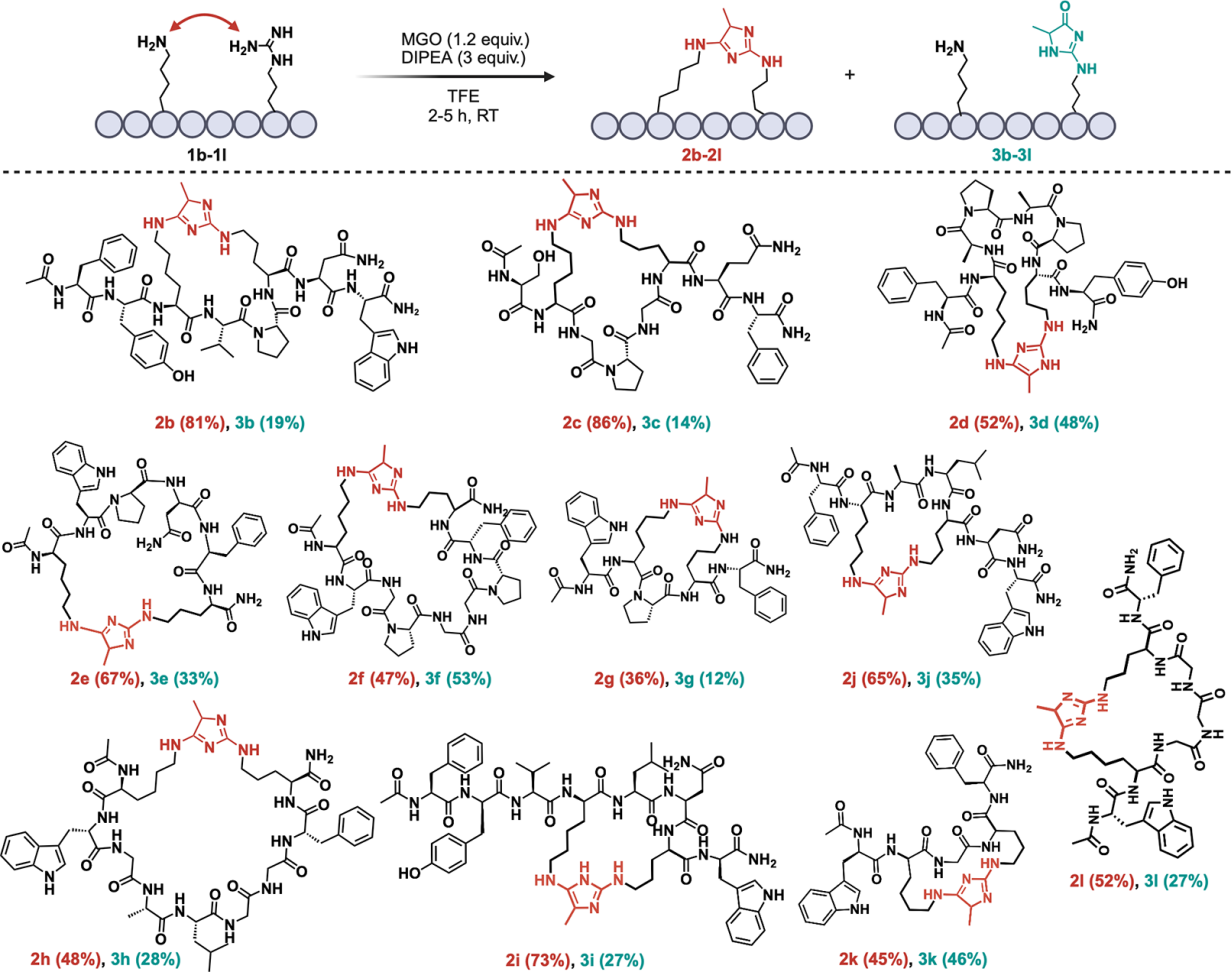
Substrate scope for TFE-mediated macrocylization of peptides generating Arg-Lys imidazole cross-link products.

However, we observed 37% of the intermolecular Lys-Lys MGO cross-link product as reported previously^21^ (Figure S1g). These results clearly highlight the crucial role of the solvent in determining the chemoselectivity of the reaction outcome. To characterize the Arg-Lys imidazole cross-link adduct, a small molecule reaction was performed with benzylamine, MGO and guanidine followed by the isolation of the product and analysis by NMR spectroscopy (Figure S2). With TFE identified as the optimized solvent for Arg-Lys imidazole cross-link, we next focused on optimizing the base to increase the conversion of **1a** to **2a** (Figure 2C; Figure S3). In addition to DIPEA, we screened several bases, including K_2_CO_3_, NaHCO_3_, Na_2_CO_3_, and Et_3_N (Figure 2C, entries 1–5; Figure S3a–f). No significant improvement in the product conversion was observed under these reaction conditions. Increasing the equivalence of MGO did not improve the conversion to the Arg-Lys imidazole cross-link product. Instead, it led to a more complex reaction profile (Figure S4). Based on these studies, we established the optimized reaction conditions as 1.2 equiv of MGO and 3 equiv of DIPEA in TFE, reacting for 2 h at room temperature (Figure S4). With the optimized conditions, we proceeded to explore the scope on peptides (**1b**–**1l**) of varying lengths and amino acid sequences, excluding cysteine, which is known to react with MGO (Figure 3).^21,24^ All modified peptides showed medium to high conversions to cyclic products with Arg-Lys imidazole cross-links at the site of cyclization (**2b**–**2l**) along with minimal formation of the Arg-MGO adducts (**3b**–**3l**) (Figure 3; Figure S5a–l).

**Figure 4:**
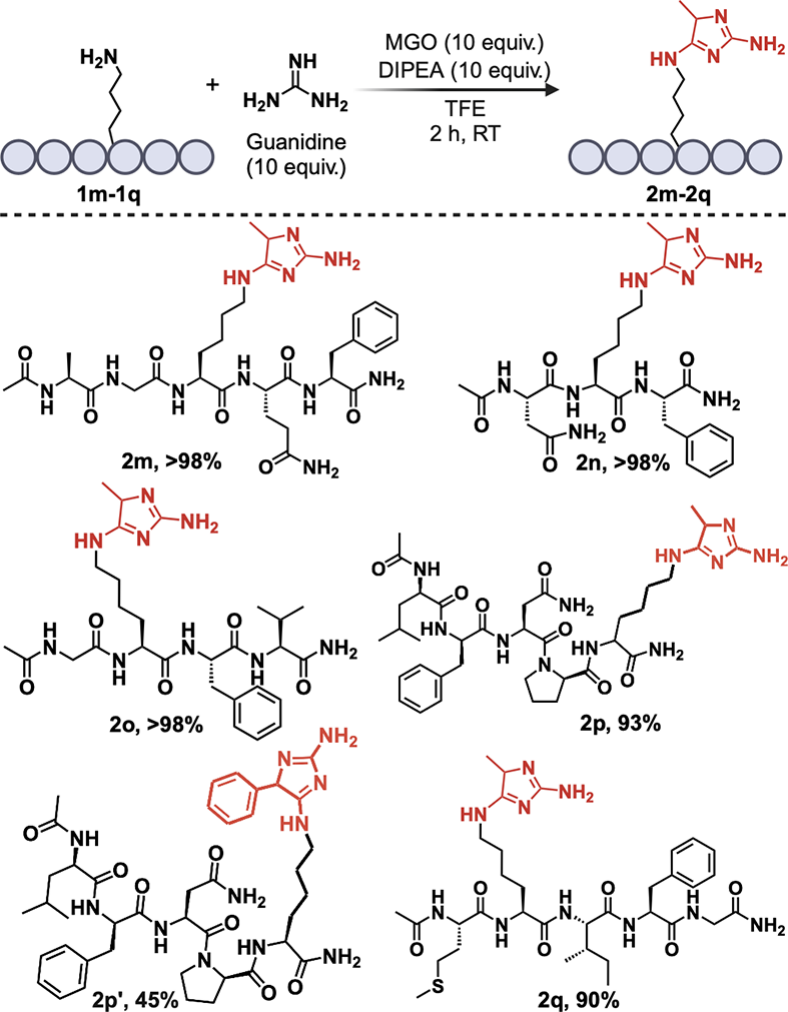
Late stage functionalization of lysine with guanidine hydrochloride to generate imidazole adducts.

Peptides **1b** and **1c** (Ac-FYKVPRNW and Ac-SKGPGRQF), which contain 2 and 3 amino acids between Arg and Lys, respectively, exhibited exceptional conversion to the desired Arg-Lys imidazole cross-link products **2b** (81%) and **2c** (86%) (Figure 3; Figures S5b,c). In contrast, peptides **1d** and **1e** (Ac-FKAPAPRY and Ac-KWPNFR), with 4 amino acids separating Arg and Lys, showed slightly reduced conversions to the cyclized Arg-Lys products **2d** (52%) and **2e** (67%), accompanied by a slight increase in the formation of Arg-MGO adducts **3d** (48%) and **3e** (33%) (Figure 3; Figure S5d,e). A similar trend was observed with peptide **1f** (Ac-KWGPGGPFR), where Arg and Lys are separated by 7 amino acids, resulting in 47% conversion to Arg-Lys imidazole product **2f** and 53% to Arg-MGO adducts **3f** (Figure 3; Figure S5f). Interestingly, when the optimized reaction conditions were applied to peptide **1g** (Ac-WKPRF), containing only one amino acid between Arg and Lys, the cyclized Arg-Lys imidazole cross-link **2g** was formed with 36% conversion. Additionally, an unexpected double addition product **2g′** was observed, suggesting the addition of two molecules of MGO (Figure S5g). Notably, when the reaction was applied to peptides lacking a turn inducing proline (Pro) amino acid such as **1h** (Ac-KWGALGGFR), **1i** (Ac-FYVKLNRW), **1j** (Ac-FKALRNW), **1k** (Ac-WKGRF), **1l** (Ac-WKGGGRF), medium to high conversions to the cyclized imidazole products (**2h–2l)** (45–73%) was observed (Figure 3; Figure S5h–l). This highlights the method’s capability to efficiently cyclize a diverse range of peptide sequences, regardless of the presence of Pro, showcasing its broad applicability and effectiveness in peptide macrocyclization. The reaction with free N-terminus peptide **1l′** WGPGRF generated imidazole cross-links **2l′** with Arg (Figure S5m). Building upon solvent-influenced chemoselective macrocyclization between Lys and Arg, we applied this chemistry for the late-stage functionalization of peptides containing Lys with imidazole adducts. To explore this, we incubated Lys-containing peptides (**1m**–**1q**) with MGO to facilitate imine formation, followed by the addition of guanidine hydrochloride at room temperature for 2 h (Figure 4, Figure S6a–h). To our delight, we observed very high conversions to imidazole products (**2m**–**2q**, 90->98%) independent of the length and sequence diversity. Peptide **1p** was subjected to phenylglyoxal to expand the substrate scope for labeling (Figure 4; Figure S6e). This resulted in 45% conversion to the labeled product **2p′**. Finally, the same reaction conditions were applied to histidine containing peptides (**1r**, **WKGHDLAM** and **1r′**, **HAF**). The reaction was not affected by the presence of histidine and generated imidazole product **2r** with high conversion (70%) (Figure S6g,h). The successful and efficient intermolecular labeling of Lys-containing peptides with external guanidine further underscores the robustness of our solvent-mediated platform for the late-stage diversification of Lys and Arg residues. We next explored the elegant use of solvent variation to generate different imidazole analogs.

**Figure 5:**
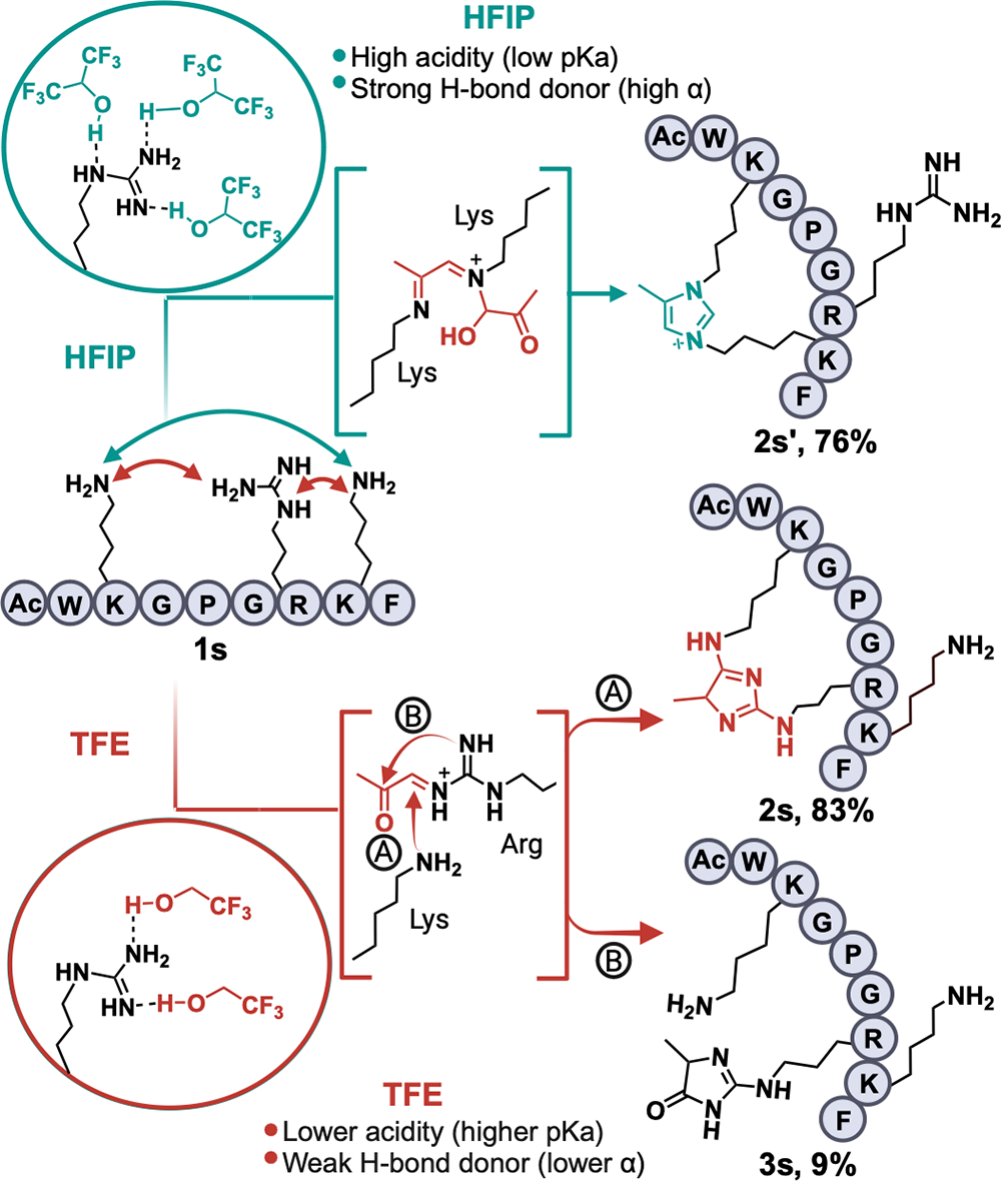
Solvent-dependent chemoselectivity switch. The high acidity and strong H-bond donating ability of HFIP stabilizes Arg, favoring a Lys-Lys imidazole cross-link. Lower acidity and weak H-bond donor ability of TFE lead to weaker stabilization of Arg, favoring formation of an Arg-Lys imidazole cross-link. Out of the two Lys on peptide **1s** any of the Lys can form a cross-link with Arg.

To achieve this, we synthesized a peptide containing an Arg residue and two Lys residues, **1s** (Ac-WKGPGRKF), and treated it with optimized reaction conditions using TFE and HFIP as solvents (Figure 5, Figures S7 and S8). As expected, when HFIP was used as the solvent, the reaction favored the formation of the Lys-Lys imidazole cross-link (**2s′**), thus validating the findings of Chen and colleagues (Figure 5, Figure S8).^21^ Importantly, no Arg-MGO adducts were detected in HFIP, suggesting that Arg reactivity is significantly reduced in this solvent (Figure S8). The selective switch to Arg-Lys imidazole cross-link was achieved in the presence of TFE (**2s**), further validating the role of solvents in the directing reaction specificity (Figure 5, Figure S7). Additionally, the formation of the Arg-MGO adduct (**3s**, 19%) was observed, reiterating the mild reactivity of Arg with MGO in TFE. Based on these observations, we propose that solvents with high acidity (low p*K*_a_) and strong hydrogen bond donating ability (high α) render Arg unreactive to MGO, likely due to increased solvation of the Arg residue (Figure 5). Conversely, solvents with low acidity (high p*K*_a_) and poor hydrogen bond donating ability (low α) increase the Arg’s reactivity toward MGO (Figure 5). Taken together, these results clearly demonstrate how the solvent reactivity spectrum of alcoholic solvents can be strategically utilized to switch the chemoselectivity of MGO reaction from Arg to Arg-Lys to Lys-Lys adducts with an increase in the number of fluorine atoms on alcoholic solvents. In conclusion, our study demonstrates the profound impact of solvent choice on the chemoselectivity of peptide macrocyclization. The ability to achieve a selective switch to Arg-Lys imidazole cross-linking using TFE highlights the critical role of solvent-dependent factors in directing reaction outcomes. Our findings reveal that nonfluorinated solvents, such as methanol and ethanol, lead to increased reactivity of Arg with MGO, resulting in undesired adduct formation, whereas fluorinated solvents, like TFE and HFIP, enable more precise control over the reaction, with TFE facilitating effective Arg-Lys cross-linking and HFIP stabilizing Arg, facilitating Lys-Lys cross-link. Additionally, the study underscores the versatility of our solvent-mediated approach for late-stage peptide functionalization, exemplified by the near-quantitative conversion of Lys-containing peptides to imidazole products with external guanidine. Overall, these results not only advance our understanding of solvent effects in peptide chemistry but also provide a robust platform for selective peptide cyclization and diversification.

## Data Availability

The data underlying this study are available in the published article and its Supporting Information.

## Supporting Information Available

General experimental procedures and characterization details, including HPLC, HRMS, and ^1^H and ^13^C NMR spectra of all reported compounds (PDF)
