# Supplementary material for: Solvent-Dependent Chemoselectivity Switch to Arg-Lys Imidazole Cross-Links
Source: Org Lett. 2024 Sep 20;26(39):8356–60. doi: 10.1021/acs.orglett.4c03101 (PMC11459505; doi:10.1021/acs.orglett.4c03101)
Supplement: Supplementary file 1 — ol4c03101_si_001.pdf [file ol4c03101_si_001.pdf]

Supporting Information

Solvent-Dependent Chemoselectivity Switch to Arg-Lys Imidazole Crosslinks

Ana Villalobos Galindo, Monika Raj*

Department of Chemistry, Emory University, Atlanta, Georgia 30322, United States

Table of Contents

1. General. 3

2. Materials. 3

3. Purification. 3

4. Instrumentation and sample analysis. 3

4a. Analytical HPLC. 3

4b. LC/MS. 4

4c. HRMS. 4

5. Fmoc Solid-Phase Peptide Synthesis (Fmoc-SPPS).[1] 4

6. General procedure for optimization of reaction conditions. 4

Supplementary Figure 1. Evaluation of Different Solvents 5

Supplemental Figure 1a. Evaluation of 10 mM Phosphate Buffer, pH7 5

Supplemental Figure 1b. Evaluation of MeOH 6

Supplemental Figure 1c. Evaluation of EtOH 8

Supplemental Figure 1d. Evaluation of TFE 10

Supplemental Figure 1e. Evaluation of 1:1 TFE:H_2O 11

Supplemental Figure 1f. Evaluation of 3:1 TFE:H_2O 13

Supplemental Figure 1g. Evaluation of HFIP 16

Supplementary Figure 2: Synthesis of N^5-benzyl-4-methyl-1*H*-imidazole-2,5-diamine 18

Supplementary Figure 3. Evaluation of Different Bases 20

Supplemental Figure 3a. Evaluation of DIPEA 20

Supplemental Figure 3b. Evaluation of K_2CO_3 22

Supplemental Figure 3c. Evaluation of $NaHCO_3$ 24

Supplemental Figure 3d. Evaluation of Na_2CO_3 26

Supplemental Figure 3e. Evaluation of Et_3N 27

Supplemental Figure 3f. Evaluation of no base 29

Supplementary Figure 4. Evaluation of Methylglyoxal Equivalences 30

General Procedure 1. Optimal Reaction Conditions for the Intramolecular Cyclization of Arginine and Lysine 30

General Procedure 2. Reaction Conditions for the Intermolecular Reaction of Guanidine Hydrochloride and Lysine 30

Supplementary Figure 5a. Cyclization of Optimization Peptide 1a 31

Supplementary Figure 5b. Cyclization of Peptide 1b 33

Supplementary Figure 5c. Cyclization of Peptide 1c 36

Supplementary Figure 5d. Cyclization of Peptide 1d 38

Supplementary Figure 5e. Cyclization of Peptide 1e 41

Supplementary Figure 5f. Cyclization of Peptide 1f 43

Supplementary Figure 5g. Cyclization of Peptide 1g 46

Supplementary Figure 5h. Cyclization of Peptide 1h 49

Supplementary Figure 5i. Cyclization of Peptide 1i 53

Supplementary Figure 5j.Cyclization of Peptide 1j 55

Supplementary Figure 5k. Cyclization of Peptide 1k 58

Supplementary Figure 5l. Cyclization of Peptide 1l 61

Supplementary Figure 5m. Cyclization of Peptide 1l’ 64

Supplementary Figure 6a: Intermolecular Labeling of Peptide 1m with Guanidine Hydrochloride67

Supplementary Figure 6b: Intermolecular Labeling of Peptide 1n with Guanidine Hydrochloride 69

Supplementary Figure 6c: Intermolecular Labeling of Peptide 1o with Guanidine Hydrochloride 71

Supplementary Figure 6d: Intermolecular Labeling of Peptide 1p with Guanidine Hydrochloride 73

Supplementary Figure 6e: Intermolecular Labeling of Peptide 1p’ with Guanidine Hydrochloride and Phenylglyoxal 75

Supplementary Figure 6f: Intermolecular Labeling of Peptide 1q with Guanidine Hydrochloride 77

Supplementary Figure 6g: Intermolecular Labeling of Peptide 1r with Guanidine Hydrochloride 79

Supplementary Figure 6h: Intermolecular Labeling of Peptide 1r’ with Guanidine Hydrochloride 82

Supplementary Figure 7: Cyclization of Peptide 1s in TFE 84

Supplementary Figure 8. Cyclization of Peptide 2s in HFIP[2] 87

References 89

1. General. All commercial materials (Sigma-Aldrich, TCI America, Oakwood Chemical) were used without further purification. All solvents were reagent or HPLC (Fisher) grade. All reactions were performed under air in glass vials. Yields refer to chromatographically pure compounds; % yields were obtained by comparing HPLC peak areas of products and starting materials. HPLC and MS were used to monitor reaction progress, and product elucidation was done using MS and NMR.

2. Materials. Fmoc-amino acids, Rink amide resin, 3-[bis(dimethylamino)methyliumyl]-3H-benzotriazol-1-oxide hexafluorophosphate (HBTU), 1-hydroxy-7-azabenzotriazole (HOAt), N,N′-iisopropylcarbodiimide(DIC) was obtained from CreoSalus (Louisville, Kentucky) and N,N-diisopropylethylamine (DIPEA) was obtained from TCI America. Piperidine, trifluoroacetic acid (TFA), were obtained from Alfa Aesar (Ward Hill, Massachusetts). N,N-dimethylformamide (DMF), dichloromethane (DCM), methanol (MeOH), acetonitrile (ACN), were obtained from VWR (100 Matsonford Road Radnor, Pennsylvania). Methylglyoxal solution and Benzylamine were obtained from Sigma-Aldrich, 2,2,2-Trifluoroethanol (TFE) and 1,1,1,3,3,3-Hexafluoro-2-propanol (HFIP) were obtained from Oakwood Chemical, Guanidine hydrochloride was obtained from Thermo Fisher Scientific.

3. Purification. HPLC: Purification of peptide starting materials was performed using high performance liquid chromatography (HPLC) on an Agilent 1100 series HPLC equipped with a C-18 reverse phase column with a particle size of 5 µm. All separations involved a mobile phase of water (solvent A) and acetonitrile (solvent B). The HPLC method used a linear gradient of 0- 80% solvent B over 30 minutes at ambient temperature with a flow rate of 1 mL/min . The eluent was monitored by absorbance at 220 nm.

4. Instrumentation and sample analysis. NMR. ^{1}H and ^{13}C spectra were acquired at 25 °C in DMSO-d^6 using an Agilent DD2 (600 MHz) spectrometer with a 3-mm He triple resonance (HCN) cryoprobe. All ^{1}H NMR chemical shifts (δ) were referenced relative to the residual DMSO-d^6 peak at 2.50 ppm or internal tetramethylsilane (TMS) at 0.00 ppm. ^{13}C NMR chemical shifts were referenced to DMSO-d^6 at 39.52 ppm. ^{13}C NMR spectra were proton decoupled. NMR spectral data are reported as chemical shift (multiplicity, coupling constants (J), integration). Multiplicity is reported as follows: singlet (s), broad singlet (br s), doublet (d), doublet of doublets (dd), doublet of triplets (td), triplet (t) and multiplet (m). Coupling constant (J) in hertz (Hz).

4a. Analytical HPLC. Analytical HPLC chromatography (HPLC) was performed on an Agilent 1100 series HPLC equipped with a 4.6 x 150 mm RediSep Prep C^{18} Aq, 100 A, 5 µm column. The reaction was monitored by analytical reverse phase HPLC using a gradient of water versus acetonitrile in linear gradients with a constant flow rate of 1 mL/min. Separations involved a mobile phase of 0.1% formic acid in water (solvent A) and 0.1 % formic acid in acetonitrile (solvent B) or mobile phase of water (solvent A) and acetonitrile (solvent B). The eluent was monitored with a detection wavelength of 220 nm.

HPLC Method A: Gradient: 0 to 80 % B (0.1% formic acid in ACN) in 30 min; 80-100 % B in 31-35 min at a flow rate of 1 mL/min.

HPLC Method B: Gradient: 0 to 30 % B (0.1% formic acid in ACN) in 30 min; 30-100 % B in 31-35 min at a flow rate of 0.5 mL/min.

4b. LC/MS. High resolution LC-MS conditions for all purified peptides: Analyses were performed on an ultraperformance LC system (ACQUITY, Waters Corp., USA) coupled with a quadrupole 3 time-of-flight mass spectrometer (Q-ToF Premier, Waters) with electrospray ionization (ESI) in positive mode using Mass lynx software (V4.1) or high-performance LC system (Agilent, 1100 series) coupled with triple quadrupole.

LC-MS (Agilent technologies 6460) with electrospray ionization (ESI) in positive mode using Agilent mass hunter (10.0). Unless otherwise mentioned a sample was injected either onto a C4 column (Phenomenex Aeris™ 3.6 µm WIDEPORE C^4 200 Å, LC Column 50 x 2.1 mm) with a 400 µL/min flow rate of mobile phase of solution A (90 % H_2O, 10 % acetonitrile and 0.1 % formic acid (FA)) and solution B (95 % acetonitrile, 5 % H_2O, and 0.1 % formic acid) beginning gradient- Time- 0 min 10 % B; 5 min 28 % B; 20 min 38 % B; 22 min 90 % B; C18 column (ACQUITY UPLC BEH 1.7 µm 1x 50 mm) with a 200 µL/min flow rate of mobile phase of solution A (90 % H_2O, 10 % acetonitrile and 0.1 % formic acid) and solution B (90 % acetonitrile, 10 % H_2O, and 0.1 % formic acid) beginning gradient 1 min 0% B; 1-10 min 100% B for chromatography analysis (or) directly injected with mobile phase 90 % H_2O: 10 % ACN, 0.1% formic acid at 400 µL/min flow rate in ESI positive mode.

4c. HRMS. High resolution MS data were acquired on Thermo Exactive Plus using a heated electrospray source. The solution was infused at a rate of 10-25 µL/min/electrospray using 3.3 KV. The typical settings were Capillary temp 320 °C. S-lens RF level was between 30-80 with an AGC setting of 1 E6. The maximum injection time as set to 50 ms. Spectra were taken at 140,000 resolutions at m/z 200 using Tune software and analyze with Thermo's Freestyle software.

5. Fmoc Solid-Phase Peptide Synthesis (Fmoc-SPPS).[1] Peptides were synthesized manually on a 0.25 mm scale using Rink amide resin. Resin was swollen with DCM for 1 h at room temperature. Fmoc was deprotected using 20% piperidine–DMF for 5 min to obtain a deprotected peptide-resin. First, Fmoc protected amino acid (1.25 mm/5 equiv.) was coupled using HOAt (1.25 mm/5 equiv.) and DIC (1.25 mm/5 equiv.) in DMF for 15 min at room temperature. Fmoc-protected amino acids (0.75 mm/3 equiv.) were sequentially coupled on the resin using HBTU (0.75 mm/3 equiv.) and DIEA (1.5 mm/6 equiv.) in DMF for 5 min at room temperature. Peptides were synthesized using standard protocols. Peptides were cleaved from the resin using a cocktail of 95:5, trifluoroacetic acid: water for 2 h. The resin was removed by filtration and the resulting solution was concentrated. The residue was diluted with ACN/ H_2O mixture. The resulting solution was purified by HPLC

6. General procedure for optimization of reaction conditions. In a one-dram vial, linear peptide **1a** (1.0 mg, 0.001 mmol, 1.0 eq) was dissolved in 250 µL of solvent followed by the addition of base (3 eq) and left to stir at room temperature for 5 minutes. Next, methylglyoxal (2 eq) was introduced. The reaction was stirred at room temperature for 2 hours and subsequently injected into the HPLC for determining the % conversion of linear peptide **1a** to the cyclized peptide **2a** and its mass confirmed with LC-MS. % conversions to the desired products and byproducts were determined by calculating the area under the LC peaks. HPLC analysis was carried out utilizing **HPLC Method A** at detection wavelength 220 nm.

Supplementary Figure 1. Evaluation of Different Solvents

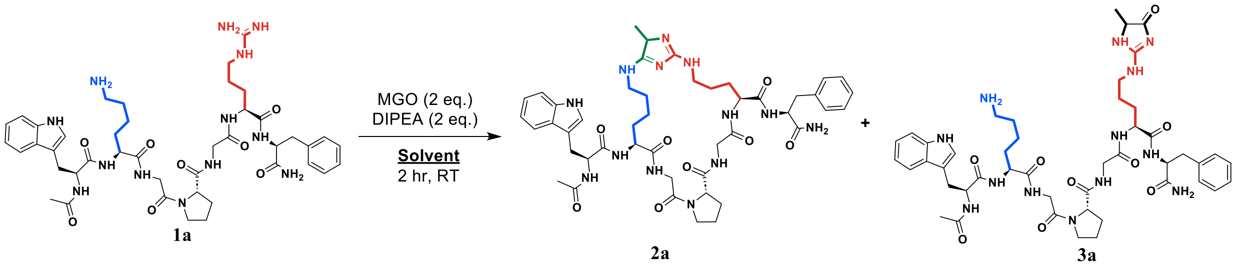

In a one-dram vial, linear peptide **1a** (1.0 mg, 0.001 mmol, 1.0 eq) was dissolved in 250 µL of solvent followed by the addition of DIPEA (3 eq) and left to stir at room temperature for 5 minutes. Next, methylglyoxal (2 eq) was introduced. The reaction was stirred at room temperature for 2 hours and subsequently injected into the HPLC for determining the % conversion of linear peptide **1a** to the cyclized peptide **2a** and its mass confirmed with LC-MS. HPLC analysis was carried out utilizing **HPLC Method A** at detection wavelength 220 nm. % conversions to the desired products and byproducts were determined by calculating the area under the LC peaks.

Entry	Solvent	HPLC Conversion to 2a
1	10 mM Phosphate Buffer, pH 7	N.R.
2	MeOH	21%
3	EtOH	22%
4	TFE	68%
5	1:1 TFE:H_2O	48%
6	3:1 TFE:H_2O	31%
7	HFIP	N.R.

N.R = No Reaction

Supplemental Figure 1a. Evaluation of 10 mM Phosphate Buffer, pH7

Ac-WKGPGRF-CONH$_2$ ($C_{43}H_{61}N_{13}O_8$) linear peptide 1a. LCMS: *m/z* 888.4847 (calcd $[M+H]^+$ = 888.4839), *m/z* 444.7463 (calcd $[M+H/2]^+$ = 444.7462) (HPLC analysis at 220 nm). Retention time in HPLC: 10.6 min

HPLC Trace of Entry 1 at 220 nm

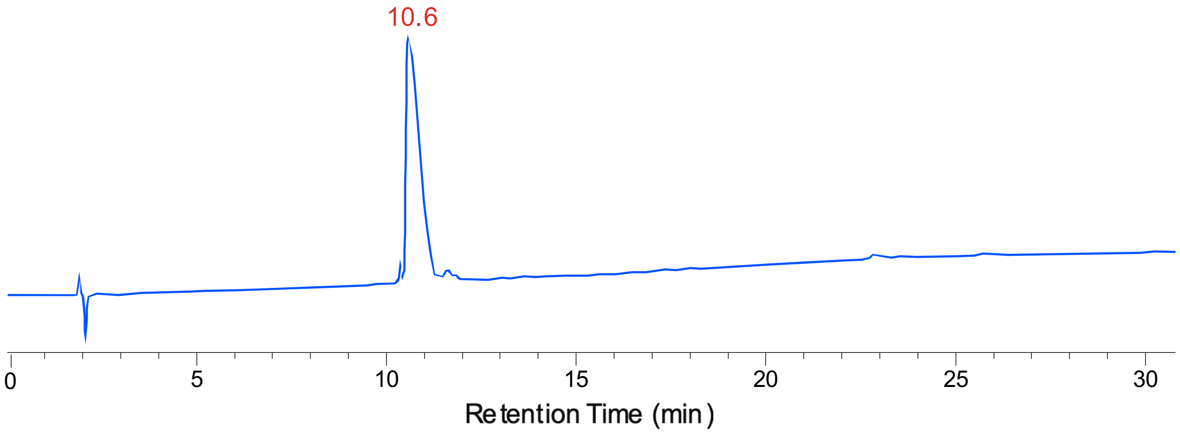

HRMS Trace of Peak at 10.6 min

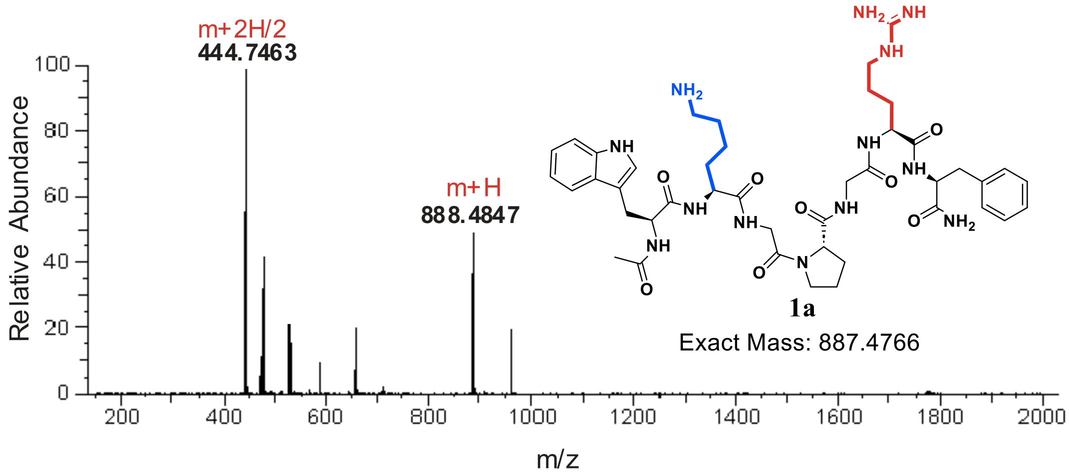

Supplemental Figure 1b. Evaluation of MeOH

Ac-WKGPGRF-CONH2 ($C_{46}H_{63}N_{13}O_9$) arginine adduct 3a. LCMS: *m/z* 942.4960 (calcd $[M+H]^+$ = 942.4944), *m/z* 471.7518 (calcd $[M+2H/2]^+$ = 471.7514), (HPLC analysis at 220 nm). Retention time in HPLC: 11.4 min

Ac-WKGPGRF-CONH2 ($C_{96}H_{131}N_{26}O_{20}^+$) Lys-Lys intermolecular peptide product. LCMS: *m/z* 984.5060 (calcd $[M+H/2]^+$ = 984.5050), (HPLC analysis at 220 nm). Retention time in HPLC: 13.2 min

Ac-WKGPGRF-CONH2 ($C_{46}H_{61}N_{13}O_8$) cyclized peptide product 2a. LCMS: *m/z* 924.4842 (calcd $[M+H]^+$ = 924.4839), (HPLC analysis at 220 nm). Retention time in HPLC: 13.9 min

Ac-WKGPGRF-CONH2 ($C_{49}H_{66}N_{13}O_{10}$) double MGO cyclized product. LCMS: *m/z* 996.5051 (calcd $[M+H]^+$ = 996.5050), (HPLC analysis at 220 nm). Retention time in HPLC: 13.9 min

HPLC Trace of Entry 2 at 220 nm

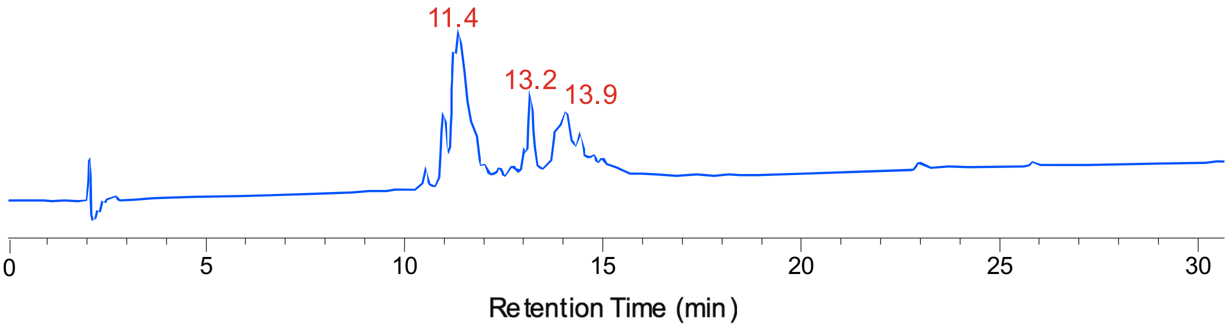

HRMS Trace of Peak at 11.4 min

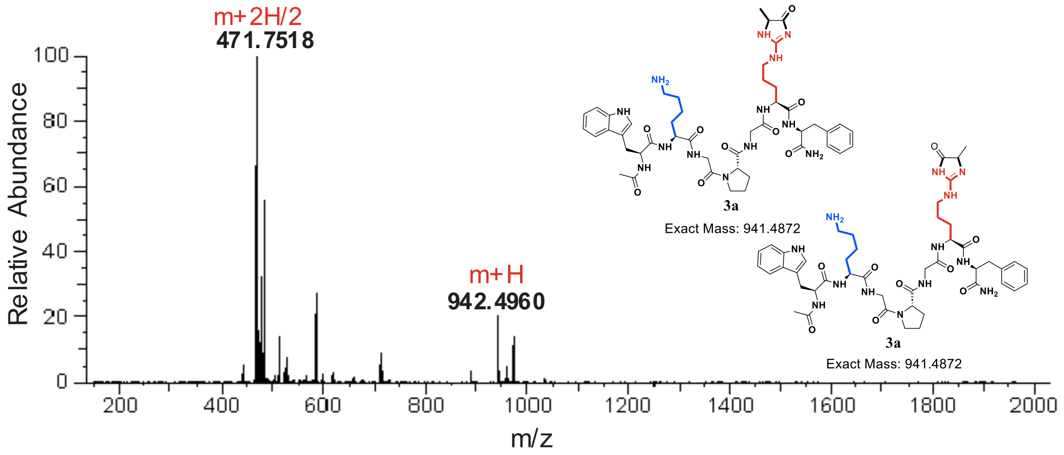

HRMS Trace of Peak at 13.2 min

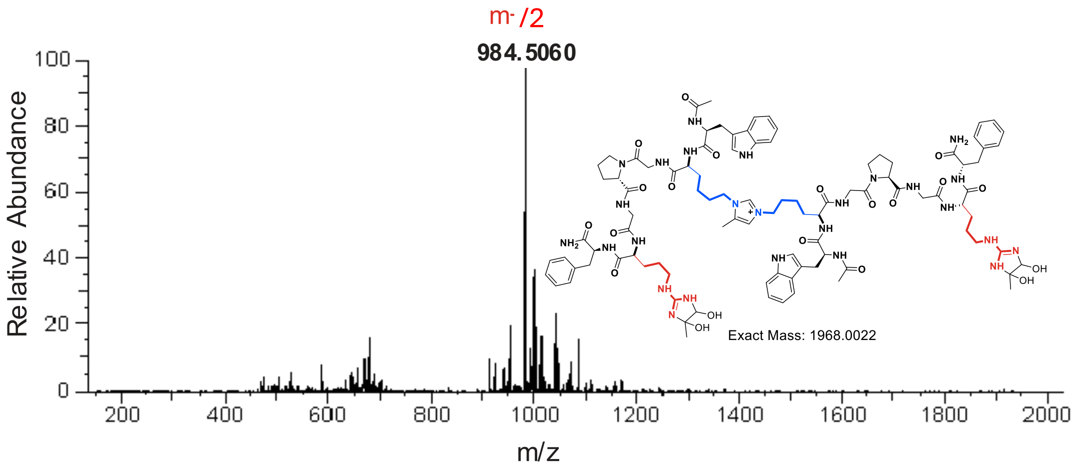

HRMS Trace of Peak at 13.9 min

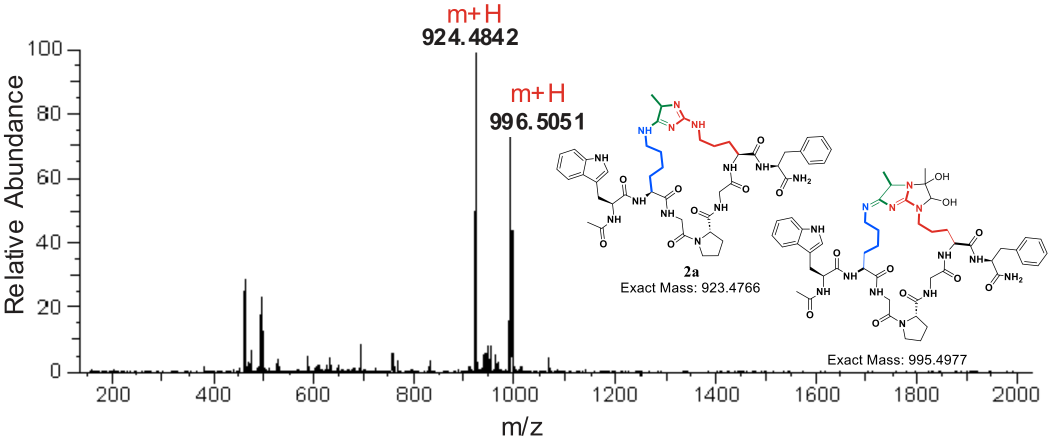

Supplemental Figure 1c. Evaluation of EtOH

Ac-WKGPGRF-CONH2 ($C_{43}H_{61}N_{13}O_8$) linear peptide 1a. LCMS: *m/z* 888.4855 (calcd $[M+H]^+$ = 888.4839), *m/z* (HPLC analysis at 220 nm). Retention time in HPLC: 10.9 min

Ac-WKGPGRF-CONH2 ($C_{46}H_{65}N_{13}O_{10}$) arginine adduct. LCMS: *m/z* 960.5077 (calcd $[M+H]^+$ = 960.5050), *m/z* 480.7574 (calcd $[M+2H/2]^+$ = 480.7567) (HPLC analysis at 220 nm). Retention time in HPLC: 10.9 min

Ac-WKGPGRF-CONH2 ($C_{46}H_{63}N_{13}O_9$) arginine adduct 3a. LCMS: *m/z* 942.4952 (calcd $[M+H]^+$ = 942.4944), *m/z* 471.7514 (calcd $[M+2H/2]^+$ = 471.7514), (HPLC analysis at 220 nm). Retention time in HPLC: 11.4 min

Ac-WKGPGRF-CONH2 ($C_{96}H_{131}N_{26}O_{20}^+$) Lys-Lys intermolecular peptide product. LCMS: *m/z* 984.5053 (calcd $[M+H/2]^+$ = 984.5050), (HPLC analysis at 220 nm). Retention time in HPLC: 13.0 min

Ac-WKGPGRF-CONH2 ($C_{46}H_{61}N_{13}O_8$) cyclized peptide product 2a. LCMS: *m/z* 924.4842 (calcd $[M+H]^+$ = 924.4839), (HPLC analysis at 220 nm). Retention time in HPLC: 13.8 min

Ac-WKGPGRF-CONH2 ($C_{49}H_{66}N_{13}O_{10}$) double MGO cyclized product. LCMS: *m/z* 996.5051 (calcd $[M+H]^+$ = 996.5050), (HPLC analysis at 220 nm). Retention time in HPLC: 13.8 min

HPLC Trace of Entry 3 at 220 nm

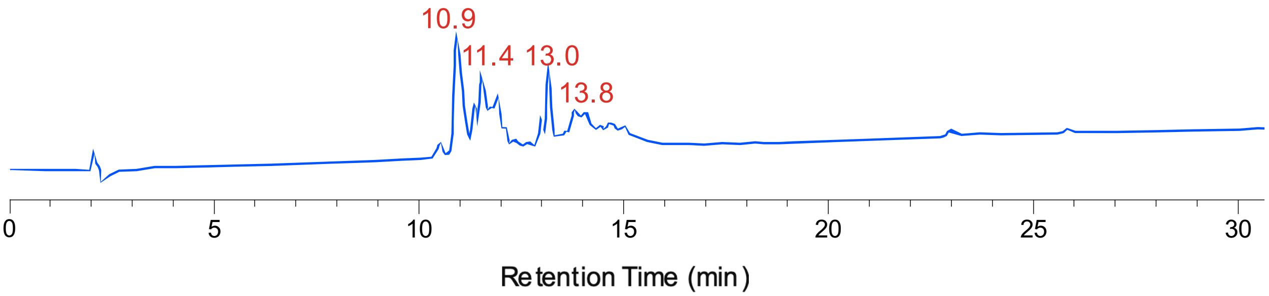

HRMS Trace of Peak at 10.9 min

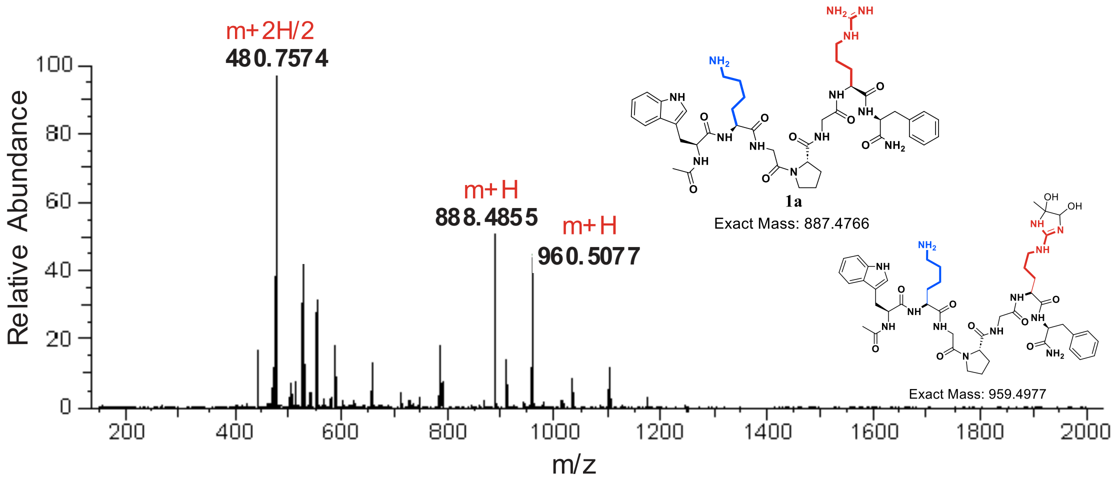

HRMS Trace of Peak at 11.4 min

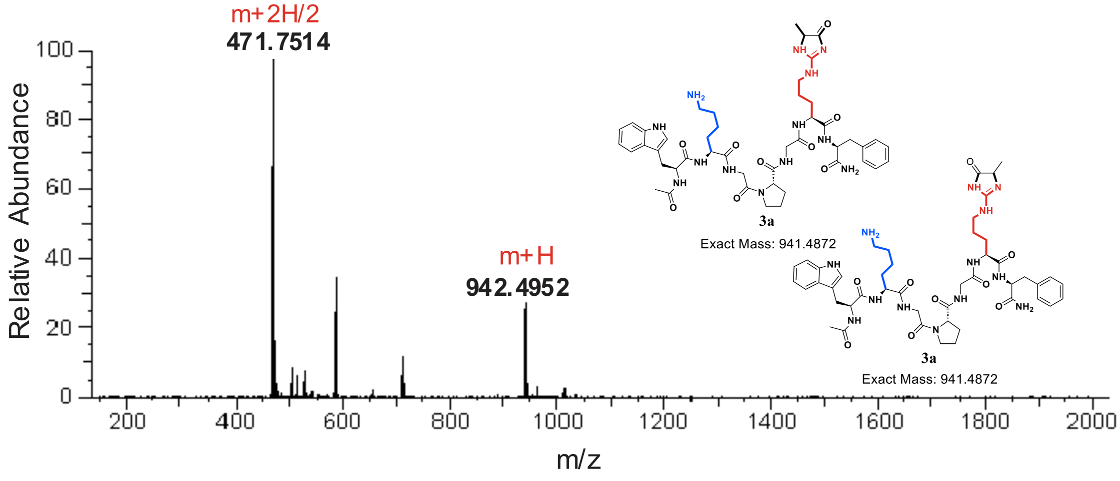

HRMS Trace of Peak at 13.0 min

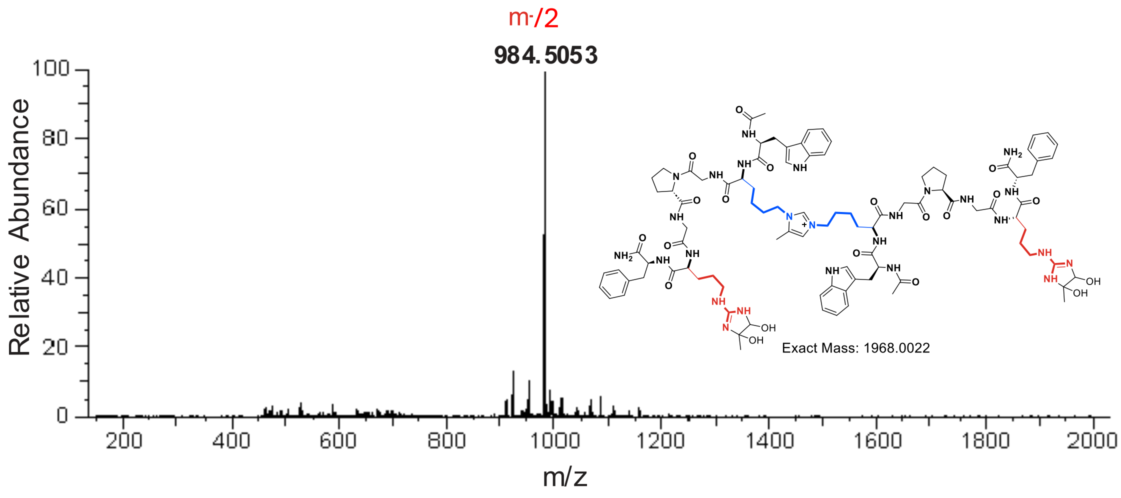

HRMS Trace of Peak at 13.8 min

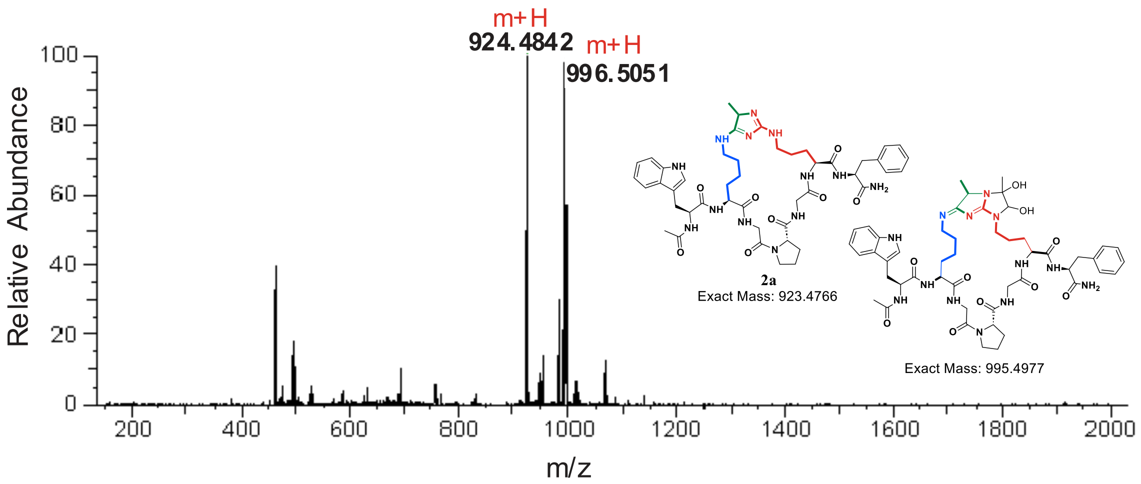

Supplemental Figure 1d. Evaluation of TFE

Ac-WKGPGRF-CONH2 ($C_{46}H_{63}N_{13}O_9$) arginine adduct 3a. LCMS: *m/z* 942.4958 (calcd $[M+H]^+$ = 942.4944), *m/z* 471.7517 (calcd $[M+2H/2]^+$ = 471.7514), (HPLC analysis at 220 nm). Retention time in HPLC: 11.2 min

Ac-WKGPGRF-CONH2 ($C_{46}H_{61}N_{13}O_8$) cyclized peptide product 2a. LCMS: *m/z* 924.4844 (13.9 min) and 924.4844 (14.0 min) (calcd $[M+H]^+$ = 924.4839), (HPLC analysis at 220 nm). Retention time in HPLC: 13.9 and 14.0 min. Two peaks in LC trace indicate the presence of two isomers.

HPLC Trace of Entry 4 at 220nm

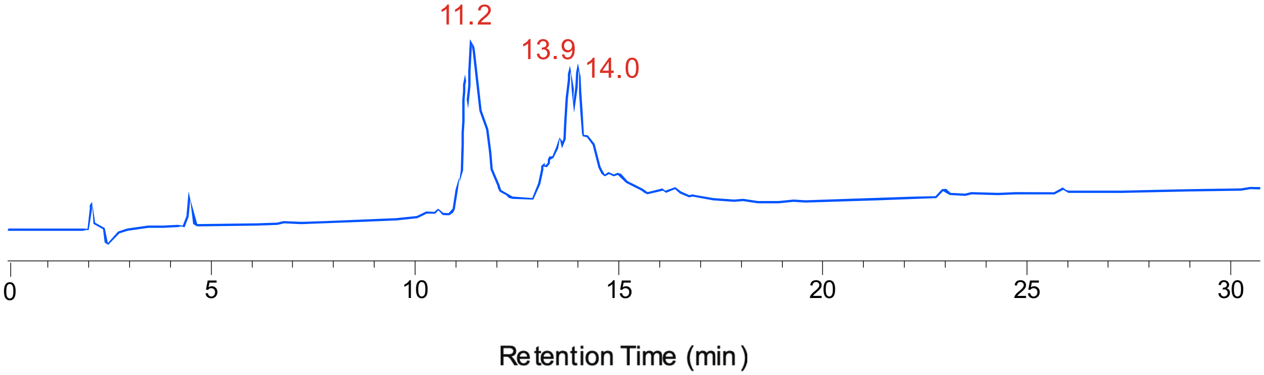

HRMS Trace of Peak at 11.2 min

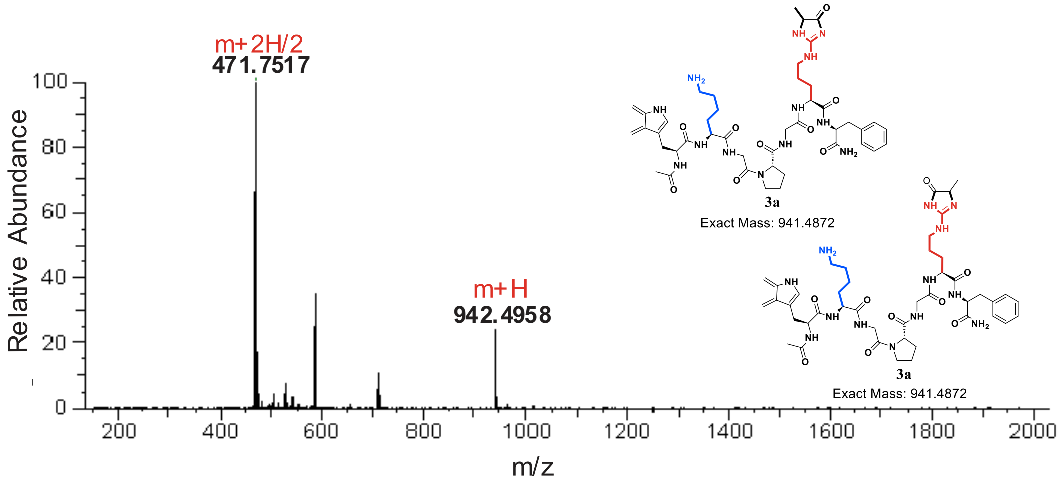

HRMS Trace of Peak at 13.9 and 14.0 min

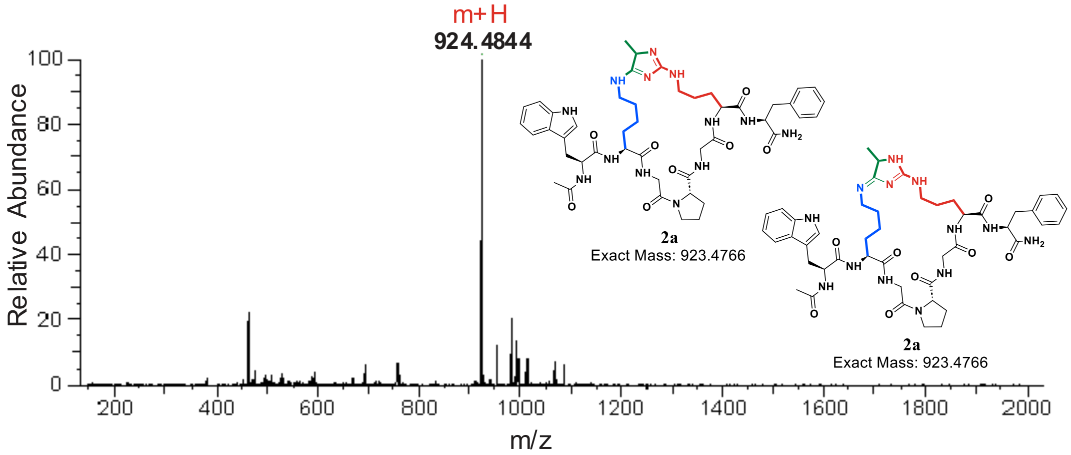

Supplemental Figure 1e. Evaluation of 1:1 TFE:H_2O

Ac-WKGPGRF-$CONH_2$ ($C_{46}H_{63}N_{13}O_9$) arginine adduct 3a. LCMS: *m/z* 942.4960 (calcd $[M+H]^+$ = 942.4944), *m/z* 471.7518 (calcd $[M+2H/2]^+$ = 471.7514), (HPLC analysis at 220 nm). Retention time in HPLC: 11.4 min

Ac-WKGPGRF-$CONH_2$ ($C_{96}H_{131}N_{26}O_{20}^+$) Lys-Lys intermolecular peptide product. LCMS: *m/z* 984.5060 (calcd $[M+H/2]^+$ = 984.5050), (HPLC analysis at 220 nm). Retention time in HPLC: 13.2 min

Ac-WKGPGRF-$CONH_2$ ($C_{46}H_{61}N_{13}O_8$) cyclized peptide product 2a. LCMS: *m/z* 924.4842 (calcd $[M+H]^+$ = 924.4839), (HPLC analysis at 220 nm). Retention time in HPLC: 13.9 min

Ac-WKGPGRF-$CONH_2$ ($C_{49}H_{66}N_{13}O_{10}$) double MGO cyclized product. LCMS: *m/z* 996.5051 (calcd $[M+H]^+$ = 996.5050), (HPLC analysis at 220 nm). Retention time in HPLC: 13.9 min

HPLC Trace of Entry 5 at 220nm

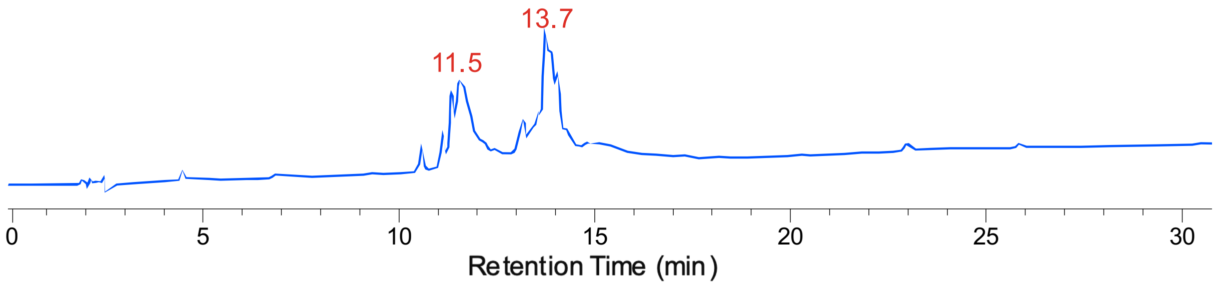

HRMS Trace of Peak at 11.5 min

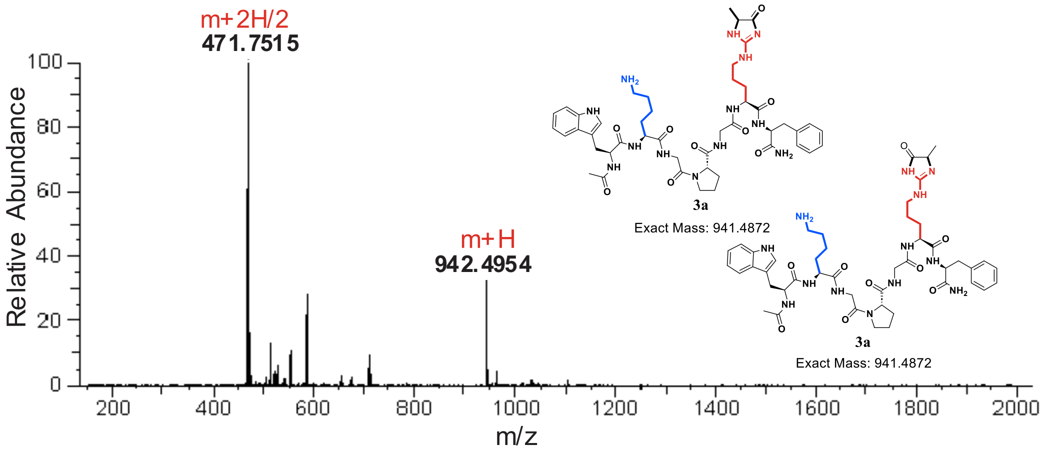

HRMS Trace of Peak at 13.7 min

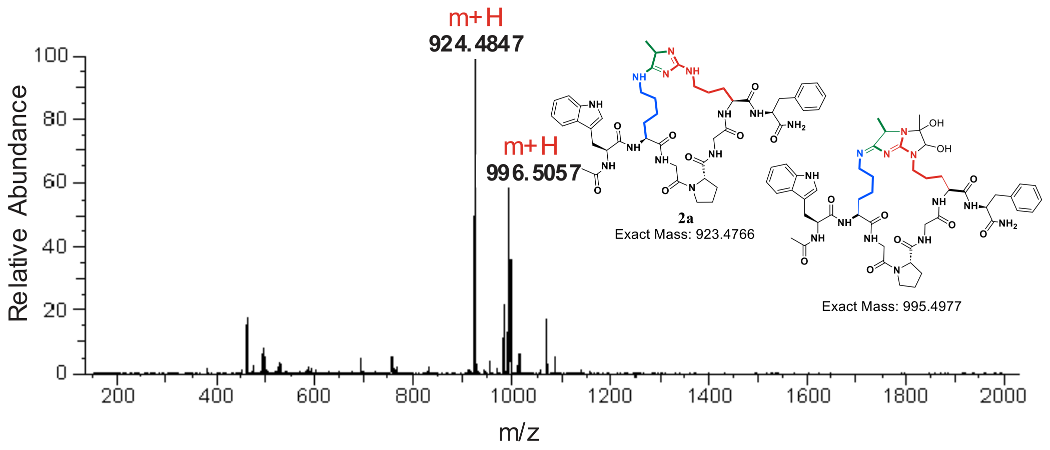

Supplemental Figure 1f. Evaluation of 3:1 TFE:H_2O

Ac-WKGPGRF-CONH$_2$ ($C_{43}H_{61}N_{13}O_8$) linear peptide 1a. LCMS: *m/z* 888.4848 (calcd $[M+H]^+$ = 888.4839), *m/z* (HPLC analysis at 220 nm). Retention time in HPLC: 11.0 min

Ac-WKGPGRF-CONH$_2$ ($C_{46}H_{65}N_{13}O_{10}$) arginine adduct. LCMS: *m/z* 960.5068 (calcd $[M+H]^+$ = 960.5050), *m/z* 480.7571 (calcd $[M+2H/2]^+$ = 480.7567) (HPLC analysis at 220 nm). Retention time in HPLC: 11.0 min

Ac-WKGPGRF-CONH$_2$ ($C_{46}H_{63}N_{13}O_9$) arginine adduct 3a. LCMS: *m/z* 942.4950 (calcd $[M+H]^+$ = 942.4944), *m/z* 471.7513 (calcd $[M+2H/2]^+$ = 471.7514), (HPLC analysis at 220 nm). Retention time in HPLC: 11.5 min

Ac-WKGPGRF-CONH$_2$ ($C_{96}H_{131}N_{26}O_{20}{}^+$) Lys-Lys intermolecular peptide product. LCMS: *m/z* 984.5055 (calcd $[M+H/2]^+$ = 984.5050), (HPLC analysis at 220 nm). Retention time in HPLC: 13.5 min

Ac-WKGPGRF-CONH$_2$ ($C_{46}H_{61}N_{13}O_8$) cyclized peptide product 2a. LCMS: *m/z* 924.4846 and 924.4844 (calcd $[M+H]^+$ = 924.4839), (HPLC analysis at 220 nm). Retention time in HPLC: 13.8 and 14.0 min. Two peaks in LC trace indicate the presence of two isomers

Ac-WKGPGRF-CONH$_2$ ($C_{49}H_{66}N_{13}O_{10}$) double MGO cyclized product. LCMS: *m/z* 996.5054 and 996.5051 (calcd $[M+H]^+$ = 996.5050), (HPLC analysis at 220 nm). Retention time in HPLC: 13.8 and 14.0 min. Two peaks in LC trace indicate the presence of two isomers

HPLC Trace of Entry 6 at 220 nm

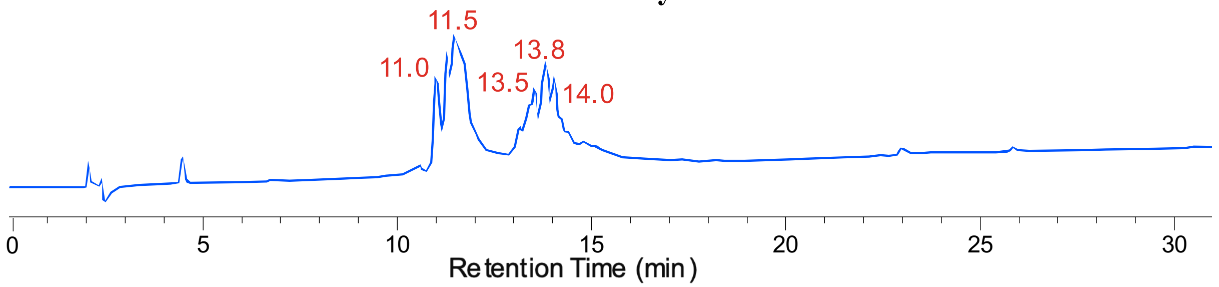

HRMS Trace of Peak at 11.0 min

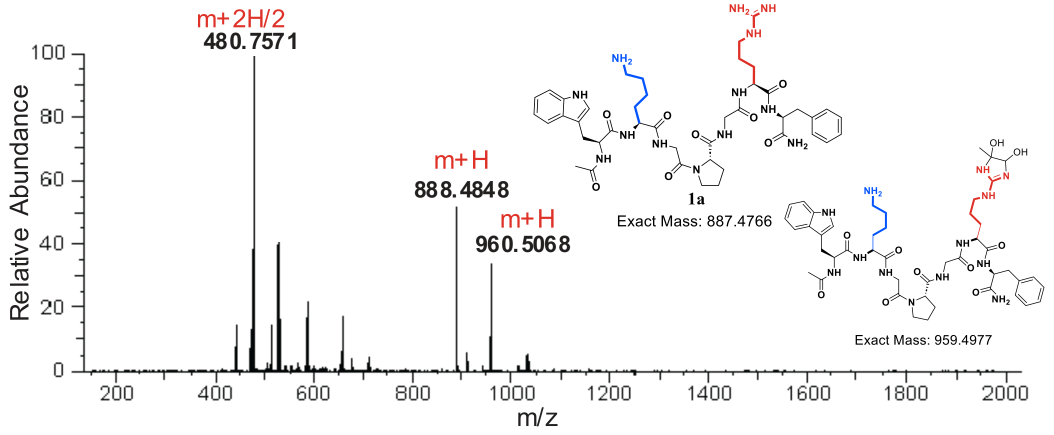

HRMS Trace of Peak at 11.5 min

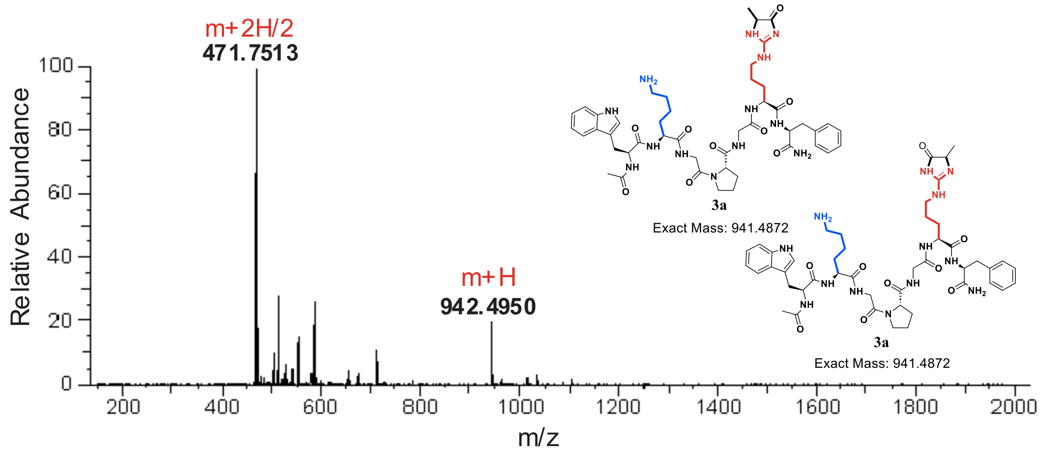

HRMS Trace of Peak at 13.5 min

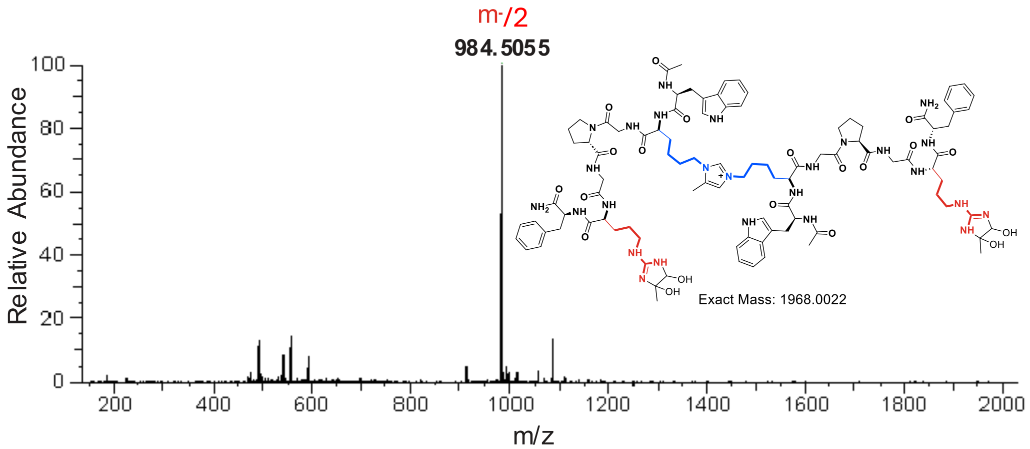

HRMS Trace of Peak at 13.8 min

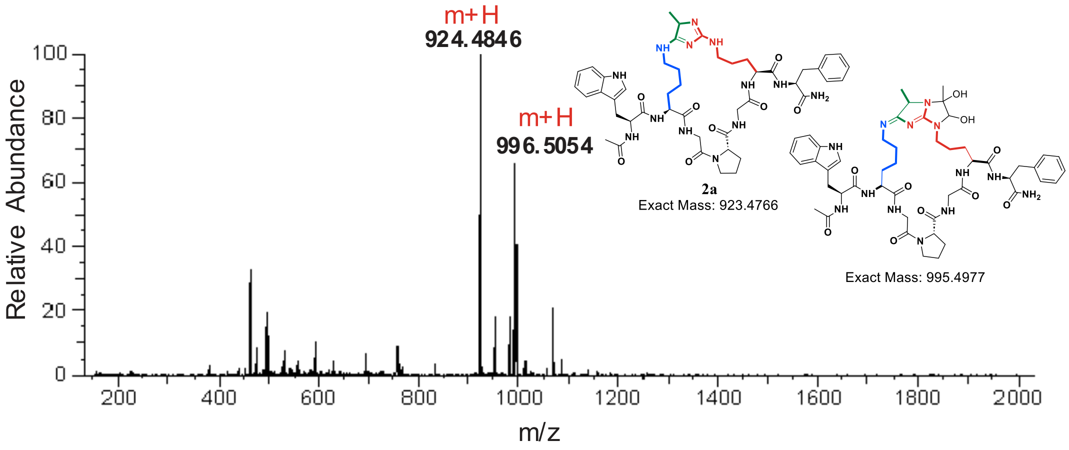

HRMS Trace of Peak at 14.0 min

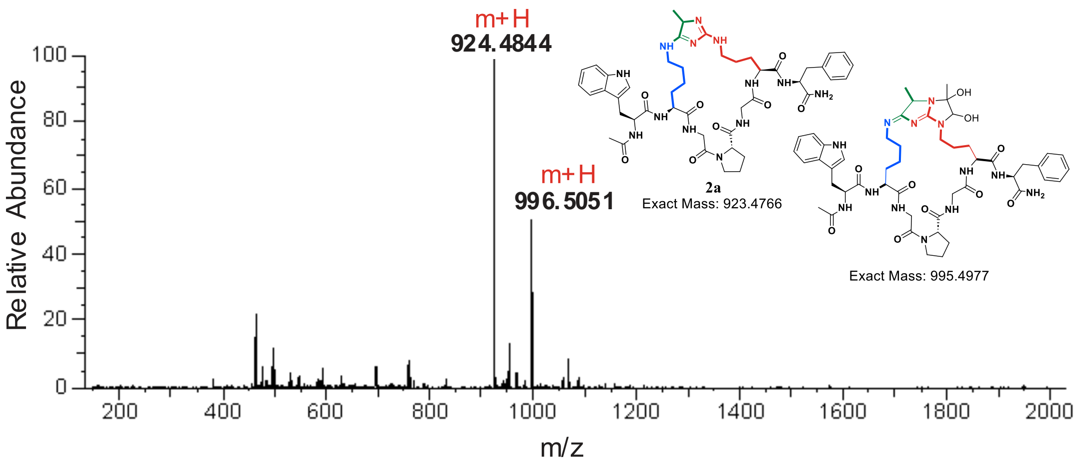

Supplemental Figure 1g. Evaluation of HFIP

Ac-WKGPGRF-CONH$_2$ ($C_{43}H_{61}N_{13}O_8$) linear peptide 1a. LCMS: *m/z* 888.4838 (calcd $[M+H]^+$ = 888.4839), *m/z* 444.7455 (calcd $[M+2H/2]^+$ = 444.7462) (HPLC analysis at 220 nm). Retention time in HPLC: 10.5 min

Ac-WKGPGRF-CONH$_2$ Lys-Lys intermolecular peptide product. LCMS: *m/z* 1823.9607 (calcd $[M+H]^+$ = 1823.9605), *m/z* 912.9859 (calcd $[M+2H/2]^+$ = 912.4839), *m/z* 608.9930 (calcd $[M+2H/3]^+$ = 608.6587) (HPLC analysis at 220 nm). Retention time in HPLC: 13.8 min

HPLC Trace of Entry 7 at 220 nm

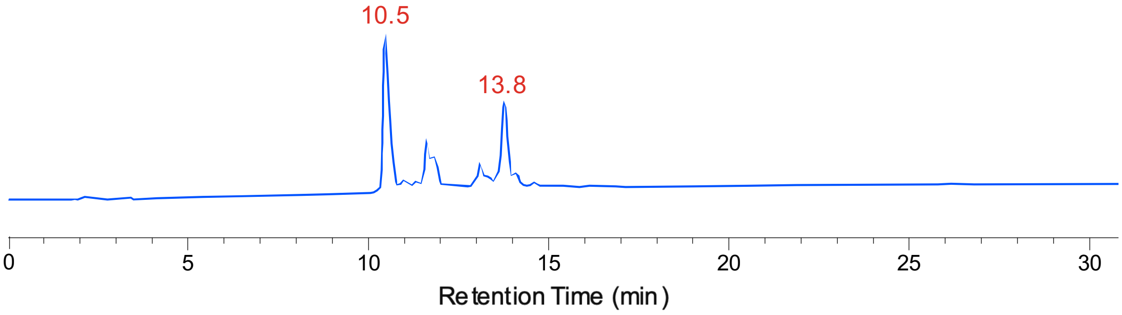

HRMS Trace of Peak at 10.5 min

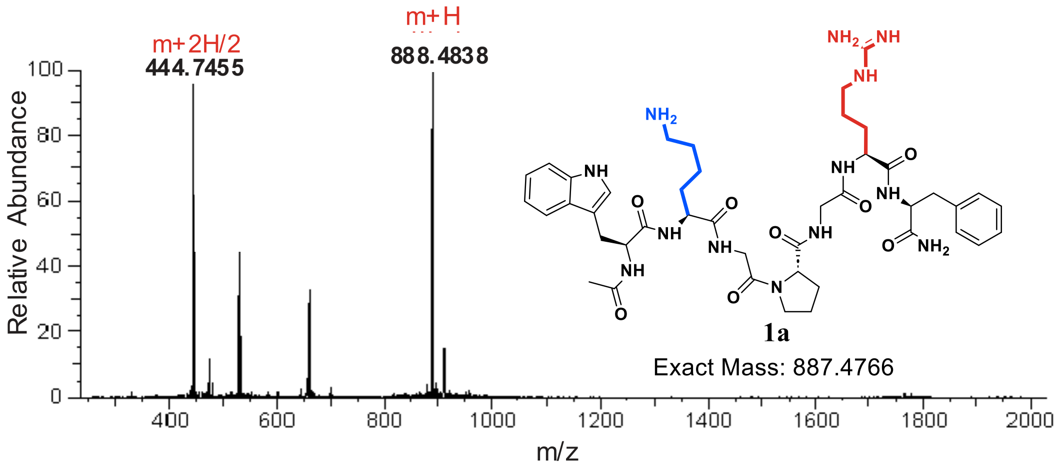

HRMS Trace of Peak at 13.8 min

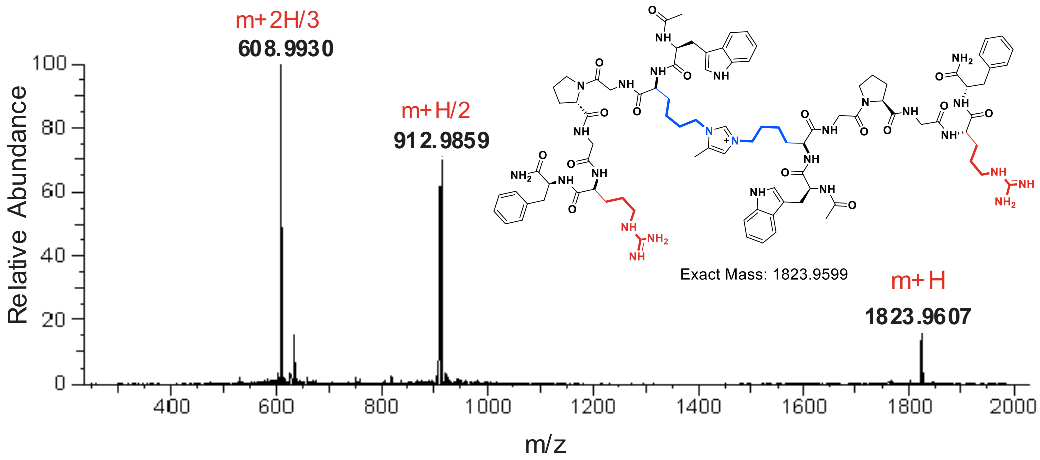

Supplementary Figure 2: Synthesis of N^5-benzyl-4-methyl-1*H*-imidazole-2,5-diamine

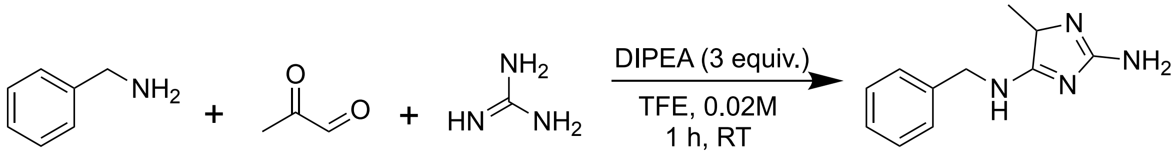

To 250 mg of benzylamine in 100 mL of TFE was added 424 µL of MGO (17.5 mmol), 1.22 mL of DIPEA (7.0 mmol) and 222 mg of guanidine hydrochloride (2.3 mmol) simultaneously and the reaction mixture was stirred at room temperature for 1 hour. Upon completion, TFE was removed in vacuo and reaction mixture was resuspended in DMSO and subsequently injected into the analytical HPLC for purification using **HPLC Method B**. Following purification, the product was lyophilized. (89%)

Cyclized product. LCMS: *m/z* 203.1293 (calcd $[M+H]^+$ = 203.1291)

^{1}H NMR (400 MHz, DMSO) δ 8.43 (s, 1H), 7.40 – 7.34 (m, 2H), 7.32 (dd, *J* = 6.8, 1.8 Hz, 2H), 7.31 – 7.27 (m, 1H), 4.69 (q, *J* = 6.8 Hz, 1H), 4.57 (d, *J* = 2.4 Hz, 2H), 1.39 (d, *J* = 6.8 Hz, 3H).

^{13}C NMR (101 MHz, DMSO) δ 182.51, 169.37, 129.02, 127.89, 127.80, 57.90, 46.42, 18.51.

^{1}H of N[5]-benzyl-4-methyl-1*H*-imidazole-2,5-diamine

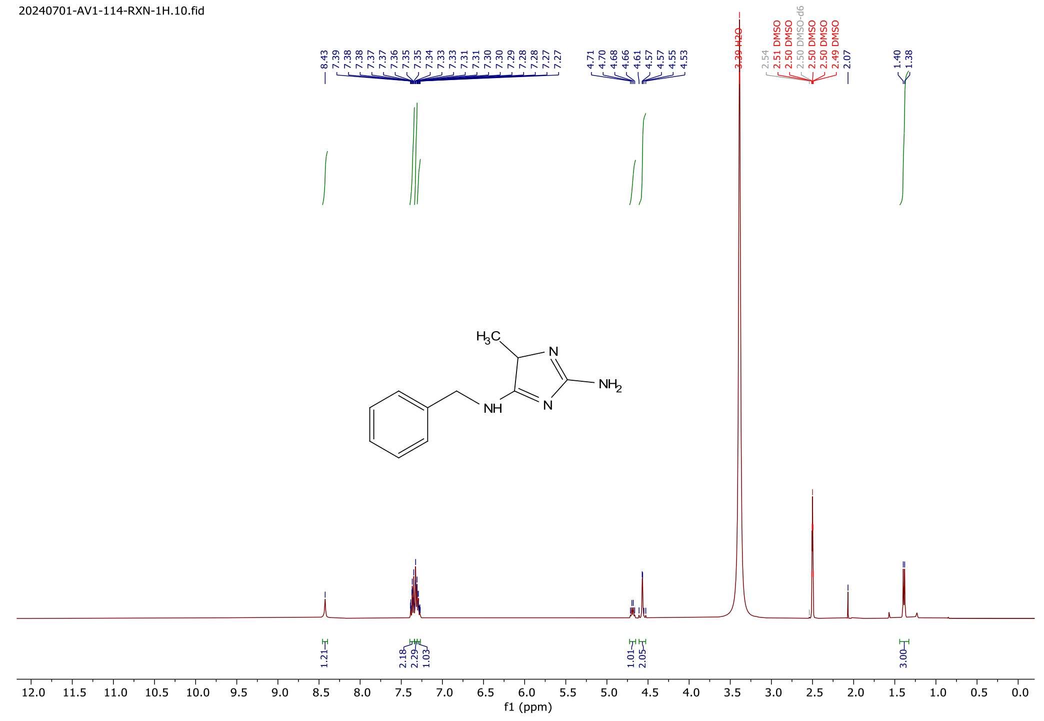

^{13}C of N[5]-benzyl-4-methyl-1*H*-imidazole-2,5-diamine

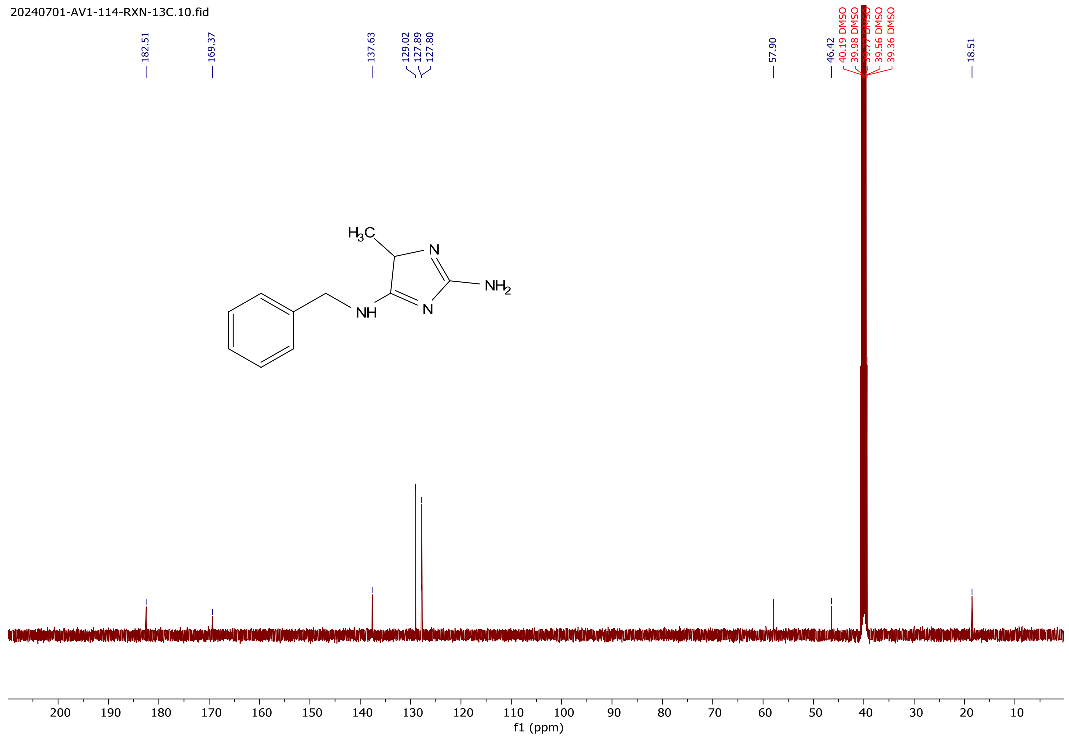

Supplementary Figure 3. Evaluation of Different Bases

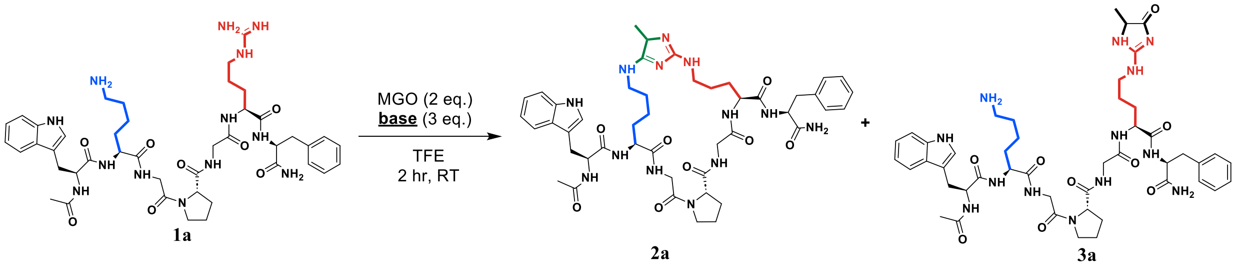

In a one-dram vial, linear peptide **1a** (1.0 mg, 0.001 mmol, 1.0 eq) was dissolved in 250 µL of TFE followed by the addition of base (3 eq) and left to stir at room temperature for 5 minutes. Next, methylglyoxal (2 eq) was introduced. The reaction was stirred at room temperature for 2 hours and subsequently injected into the HPLC for determining the % conversion of linear peptide **1a** to the cyclized peptide **2a** and its mass confirmed with LC-MS. HPLC analysis was carried out utilizing **HPLC Method A** at detection wavelength 220 nm.

Entry	Base	HPLC Conversion to 2a
1	DIPEA	68%
2	K_2CO_3	54%
3	$NaHCO_3$	62%
4	Na_2CO_3	45%
5	Et_3N	62%
6	None	N.R.

N.R. = No Reaction

Supplemental Figure 3a. Evaluation of DIPEA

Ac-WKGPGRF-CONH$_2$ ($C_{46}H_{63}N_{13}O_9$) arginine adduct 3a. LCMS: *m/z* 942.4953 (11.1 min) and 942.4944 (11.3 min) (calcd $[M+H]^+$ = 942.4944), *m/z* 471.7513 (11.1 min) and 471.7509 (11.3 min) (calcd $[M+2H/2]^+$ = 471.7514), (HPLC analysis at 220 nm). Retention time in HPLC: 11.1 and 11.3 min. Two peaks in LC trace indicate the presence of two isomers.

Ac-WKGPGRF-CONH$_2$ ($C_{46}H_{61}N_{13}O_8$) cyclized peptide product 2a. LCMS: *m/z* 924.4842 (13.5 min) and 924.4843 (13.7 min) (calcd $[M+H]^+$ = 924.4839) (HPLC analysis at 220 nm). Retention time in HPLC: 13.5 and 13.7 min. Two peaks in LC trace indicate the presence of two isomers.

HPLC Trace of Entry 1 at 220nm

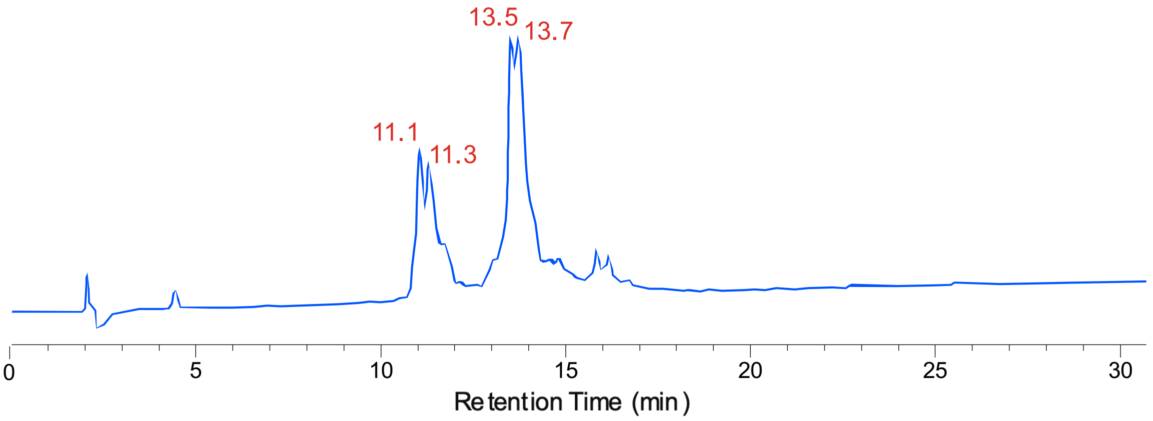

HRMS Trace of Peak at 11.1 min

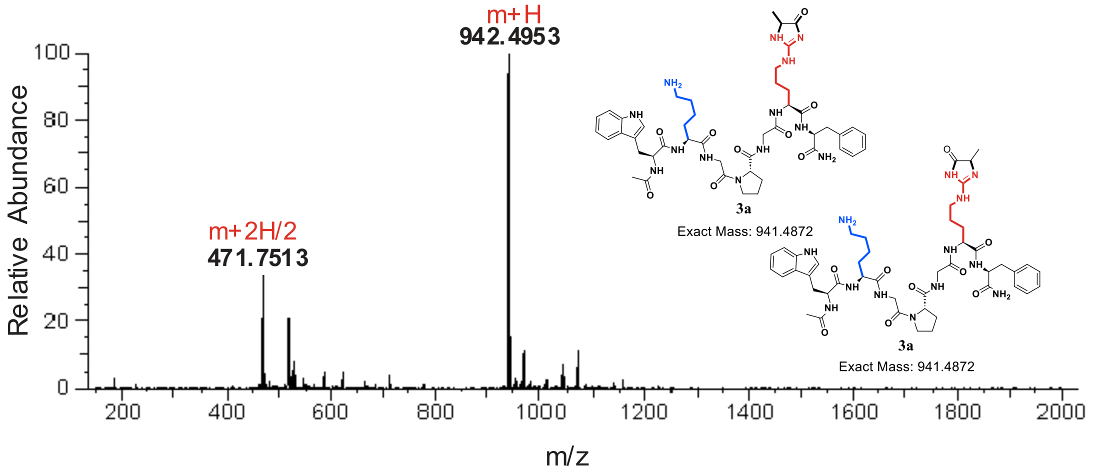

HRMS Trace of Peak at 11.3 min

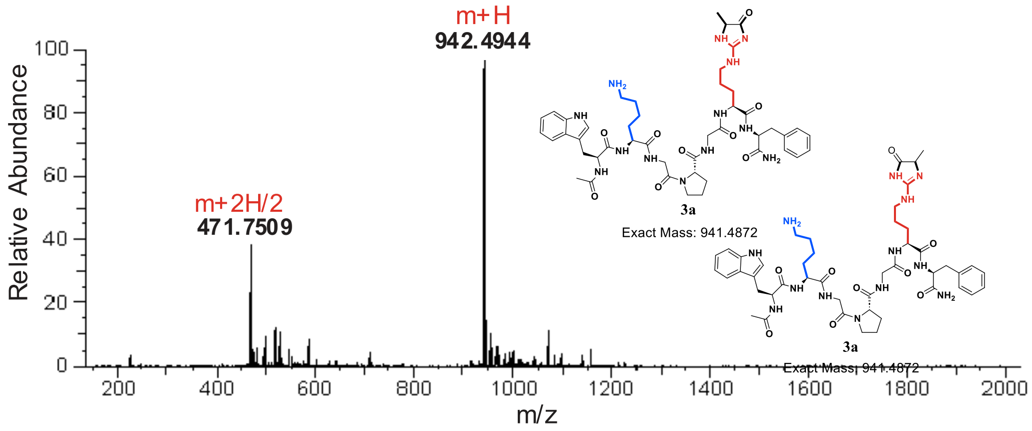

HRMS Trace of Peak at 13.5 and 13.7 min

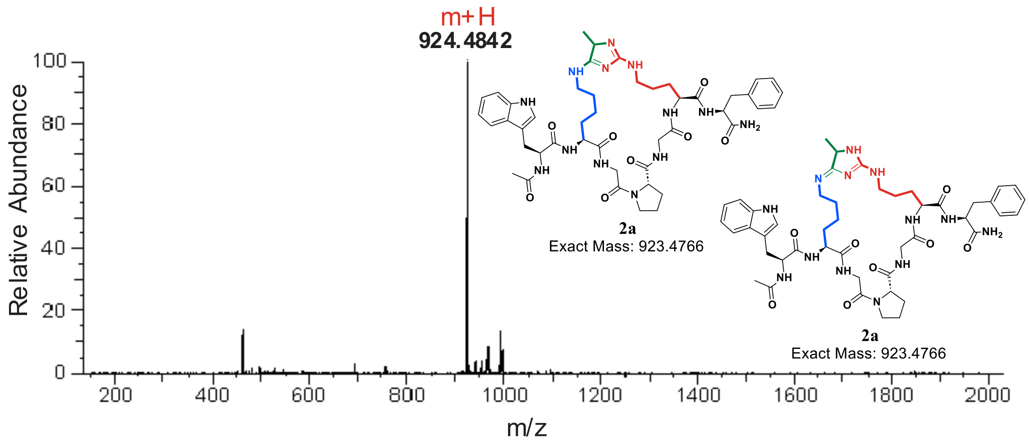

Supplemental Figure 3b. Evaluation of K_2CO_3

Ac-WKGPGRF-CONH$_2$ ($C_{46}H_{63}N_{13}O_9$) arginine adduct 3a. LCMS: *m/z* 942.4949 (11.0 min) and 942.4943 (11.3 min) (calcd $[M+H]^+$ = 942.4944), *m/z* 471.7511 (11.0 min) and 471.7509 (11.3 min) (calcd $[M+2H/2]^+$ = 471.7514), (HPLC analysis at 220 nm). Retention time in HPLC: 11.0 and 11.3 min. Two peaks in LC trace indicate the presence of two isomers.

Ac-WKGPGRF-CONH$_2$ ($C_{46}H_{61}N_{13}O_8$) cyclized peptide product 2a. LCMS: *m/z* 924.4851 (13.6 min) and 924.4848 (13.8 min) (calcd $[M+H]^+$ = 924.4839), (HPLC analysis at 220 nm). Retention time in HPLC: 13.6 and 13.8 min. Two peaks in LC trace indicate the presence of two isomers.

HPLC Trace of Entry 2 at 220 nm

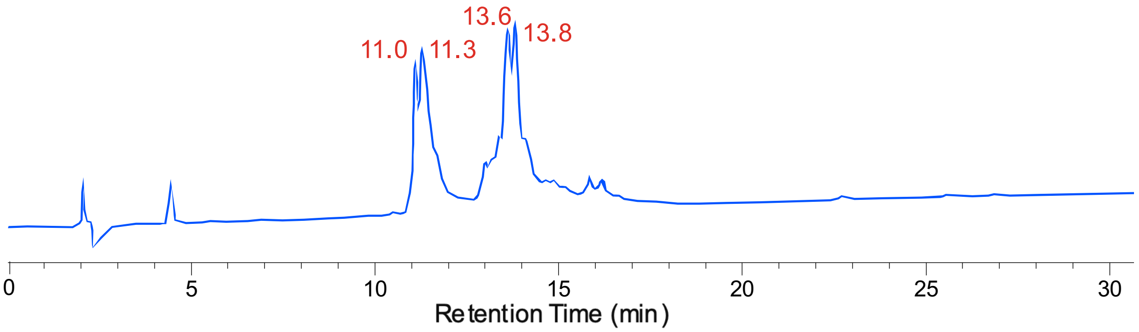

HRMS Trace of Peak at 11.0 min

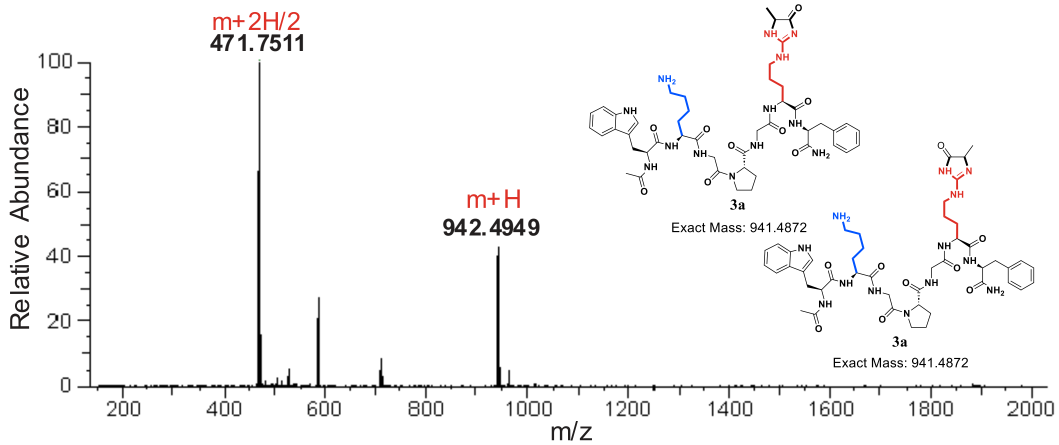

HRMS Trace of Peak at 11.3 min

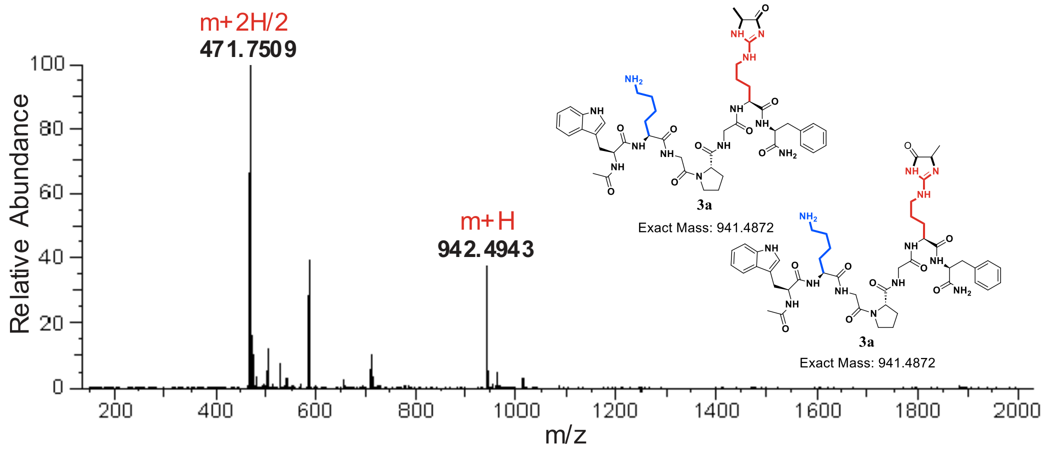

HRMS Trace of Peak at 13.6 and 13.8 min

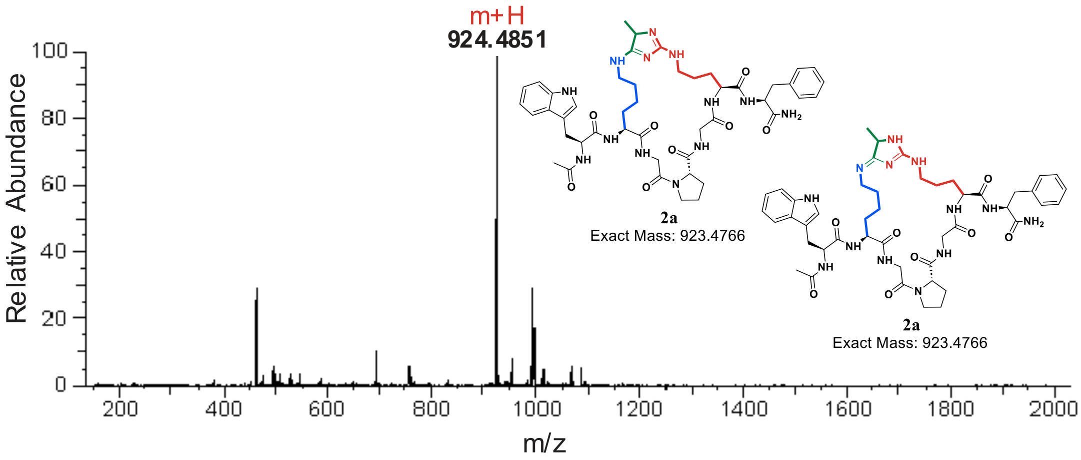

Supplemental Figure 3c. Evaluation of $NaHCO_3$

Ac-WKGPGRF-CONH$_2$ ($C_{43}H_{61}N_{13}O_8$) linear peptide 1a. LCMS: *m/z* 888.4838 (calcd $[M+H]^+$ = 888.4839), *m/z* 444.7457 (calcd $[M+2H/2]^+$ = 444.7462) (HPLC analysis at 220 nm). Retention time in HPLC: 10.8 min

Ac-WKGPGRF-CONH$_2$ ($C_{46}H_{65}N_{13}O_{10}$) arginine adduct. LCMS: *m/z* 960.5053 (calcd $[M+H]^+$ = 960.5050), *m/z* 480.7562 (calcd $[M+2H/2]^+$ = 480.7567) (HPLC analysis at 220 nm), (HPLC analysis at 220 nm). Retention time in HPLC: 10.8 min

Ac-WKGPGRF-CONH$_2$ ($C_{46}H_{63}N_{13}O_9$) arginine adduct 3a. LCMS: *m/z* 942.4942 (11.0 min) and 942.4958 (11.3 min) (calcd $[M+H]^+$ = 942.4944), *m/z* 471.7508 (11.0 min) and 471.7516 (11.3 min) (calcd $[M+2H/2]^+$ = 471.7514), (HPLC analysis at 220 nm). Retention time in HPLC: 11.0 and 11.3 min. Two peaks in LC trace indicate the presence of two isomers.

Ac-WKGPGRF-CONH$_2$ ($C_{46}H_{61}N_{13}O_8$) cyclized peptide product 2a. LCMS: *m/z* 924.4841 (13.5 min) and 924.4843 (13.8 min) (calcd $[M+H]^+$ = 924.4839), *m/z* 462.7457 and 462.7458 (calcd $[M+2H/2]^+$ =462.7462) (HPLC analysis at 220 nm). Retention time in HPLC: 13.5 and 13.8 min. Two peaks in LC trace indicate the presence of two isomers.

HPLC Trace of Entry 3 at 220 nm

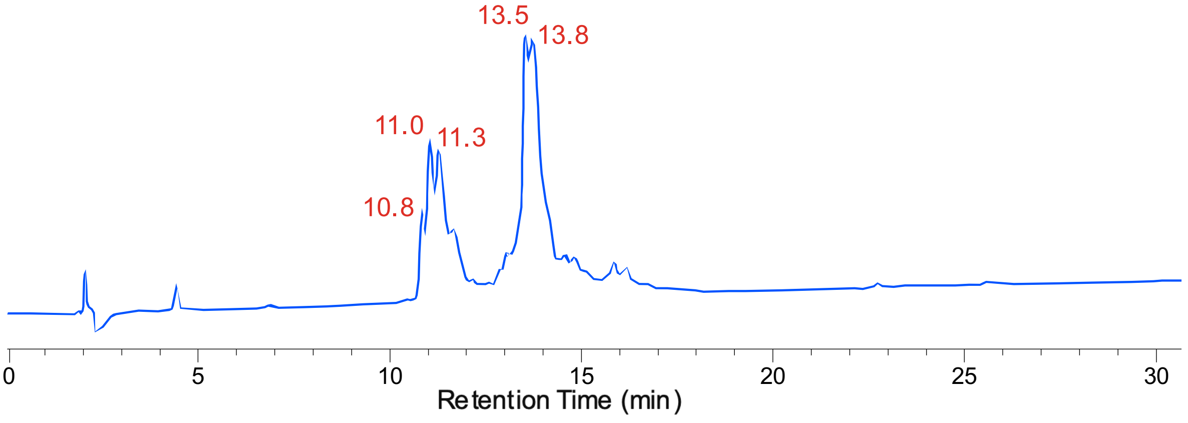

HRMS Trace of Peak at 10.8 min

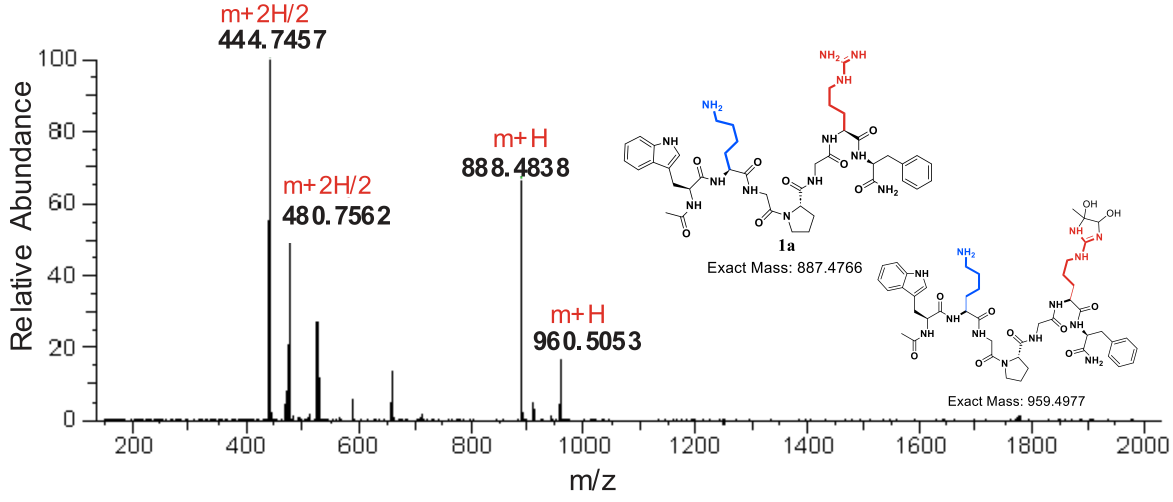

HRMS Trace of Peak at 11.0 min

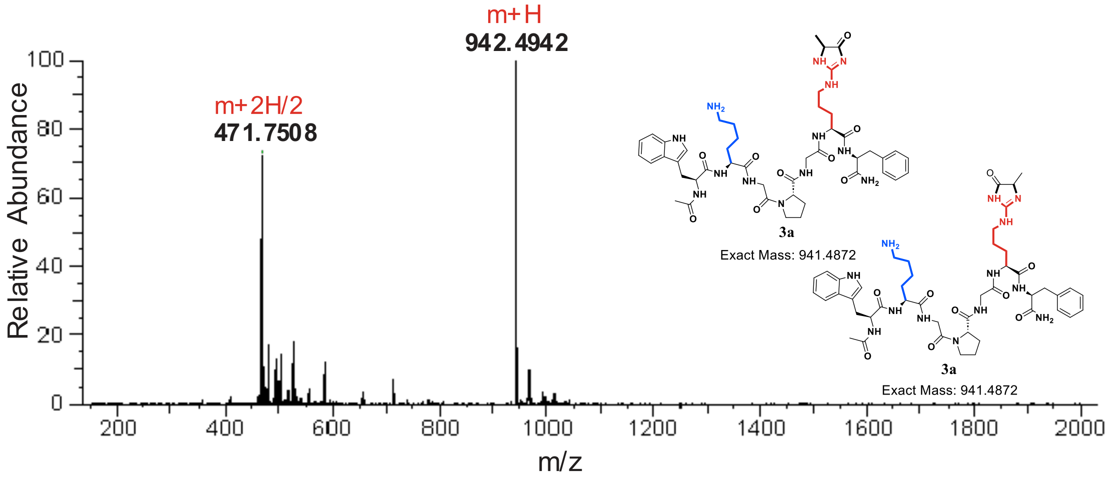

HRMS Trace of Peak at 11.3 min

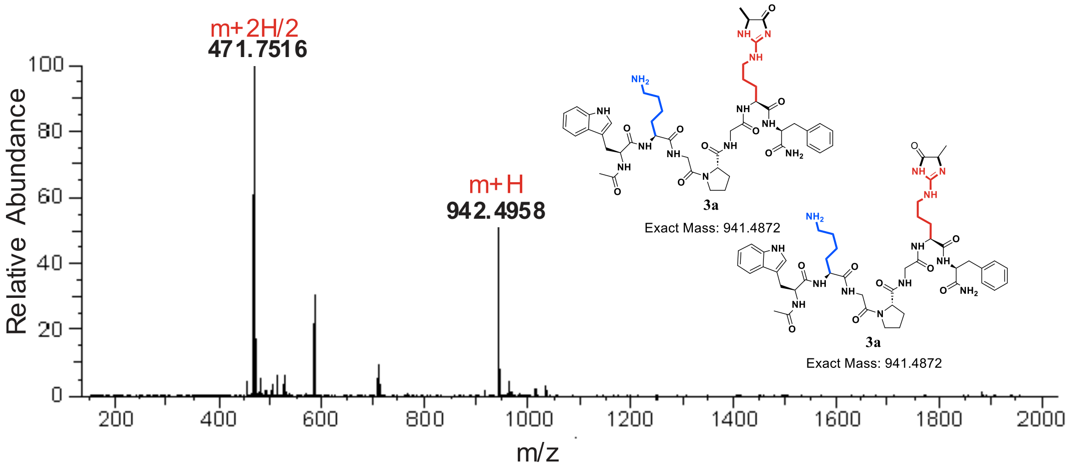

HRMS Trace of Peak at 13.5 and 13.8 min

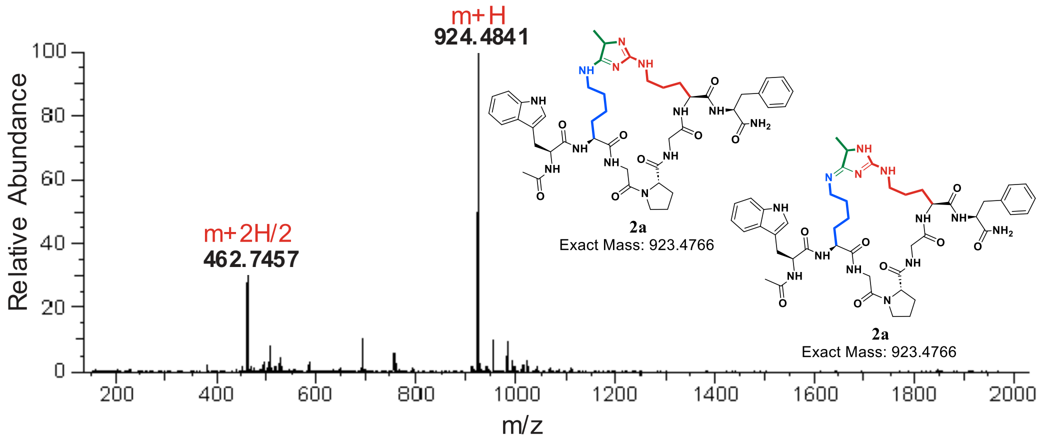

Supplemental Figure 3d. Evaluation of Na_2CO_3

Ac-WKGPGRF-CONH$_2$ ($C_{46}H_{63}N_{13}O_9$) arginine adduct 3a. LCMS: *m/z* 942.4941 (11.2 min) and 942.4938 (11.3 min) (calcd $[M+H]^+$ = 942.4944), *m/z* 471.7508 (11.2 min) and 471.7506 (11.3 min) (calcd $[M+2H/2]^+$ = 471.7514), (HPLC analysis at 220 nm). Retention time in HPLC: 11.2 and 11.3 min. Two peaks in LC trace indicate the presence of two isomers.

Ac-WKGPGRF-CONH$_2$ ($C_{46}H_{61}N_{13}O_8$) cyclized peptide product 2a. LCMS: *m/z* 924.4845 (13.8 min) and 924.4846 (14.0 min) (calcd $[M+H]^+$ = 924.4839) (HPLC analysis at 220 nm). Retention time in HPLC: 13.8 and 14.0 min. Two peaks in LC trace indicate the presence of two isomers.

HPLC Trace of Entry 4 at 220nm

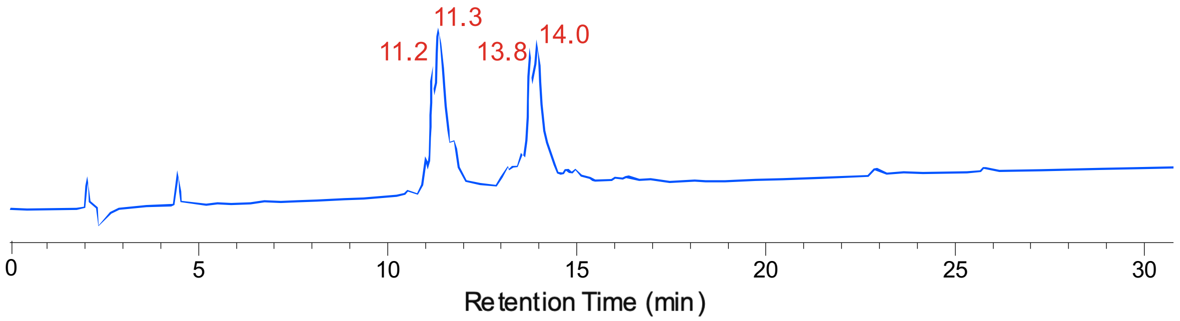

HRMS Trace of Peak at 11.2 min

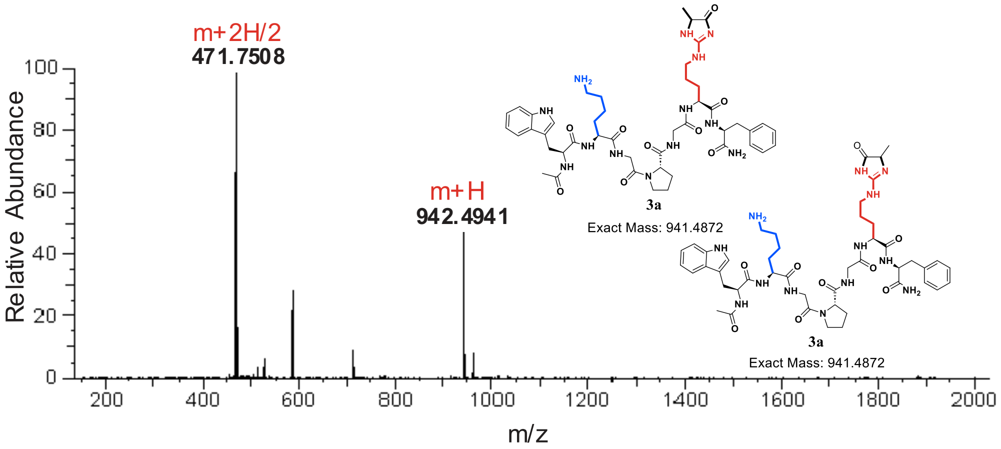

HRMS Trace of Peak at 11.3 min

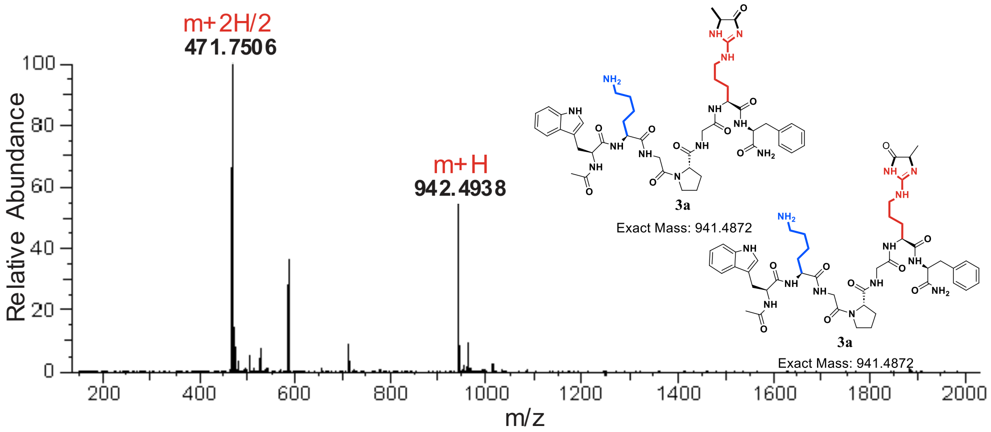

HRMS Trace of Peak at 13.8 and 14.0 min

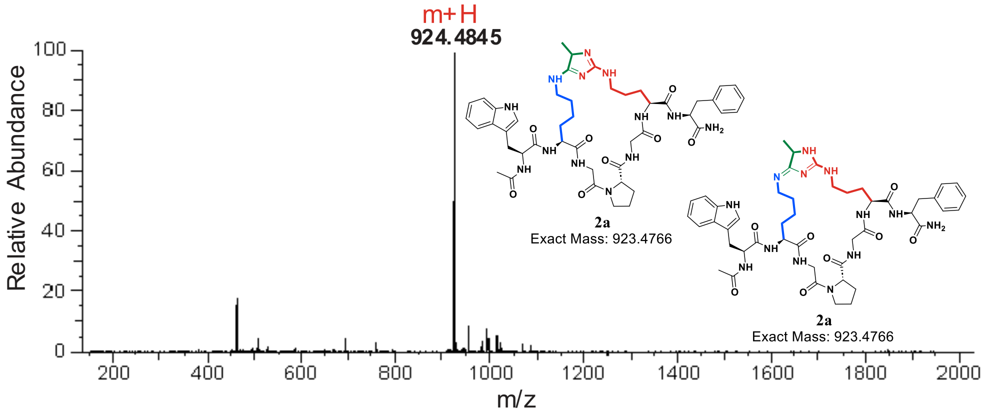

Supplemental Figure 3e. Evaluation of Et_3N

Ac-WKGPGRF-CONH$_2$ ($C_{46}H_{63}N_{13}O_9$) arginine adduct 3a. LCMS: *m/z* 942.4940 (11.0 min) and 942.4953 (11.3 min) (calcd $[M+H]^+$ = 942.4944), *m/z* 471.7508 (11.0 min) and 471.7514 (11.3 min) (calcd $[M+2H/2]^+$ = 471.7514), (HPLC analysis at 220 nm). Retention time in HPLC: 11.0 and 11.3 min. Two peaks in LC trace indicate the presence of two isomers

Ac-WKGPGRF-CONH$_2$ ($C_{46}H_{61}N_{13}O_8$) cyclized peptide product 2a. LCMS: *m/z* 924.4842 (13.6 min) and 924.4840 (13.8 min) (calcd $[M+H]^+$ = 924.4839), *m/z* 462.7458 (13.6 min) and 462.7457 (13.8 min) (calcd $[M+2H/2]^+$ =462.7462) (HPLC analysis at 220 nm). Retention time in HPLC: 13.6 and 13.8 min. Two peaks in LC trace indicate the presence of two isomers

HPLC Trace of Entry 5 at 220nm

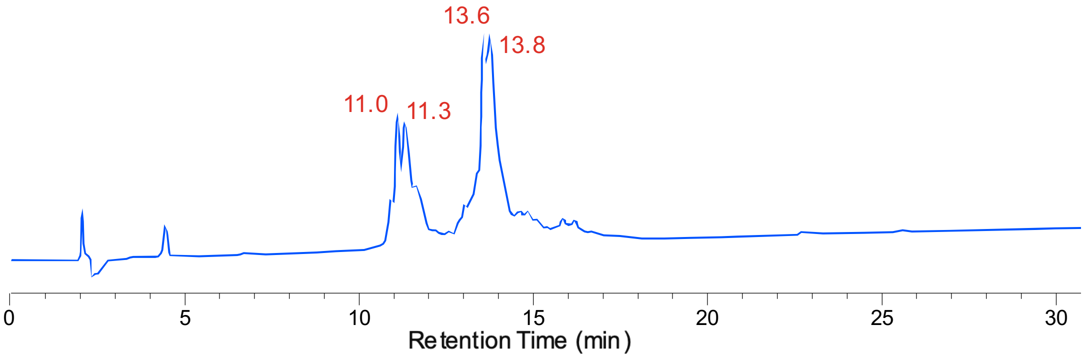

HRMS Trace of Peak at 11.0 min

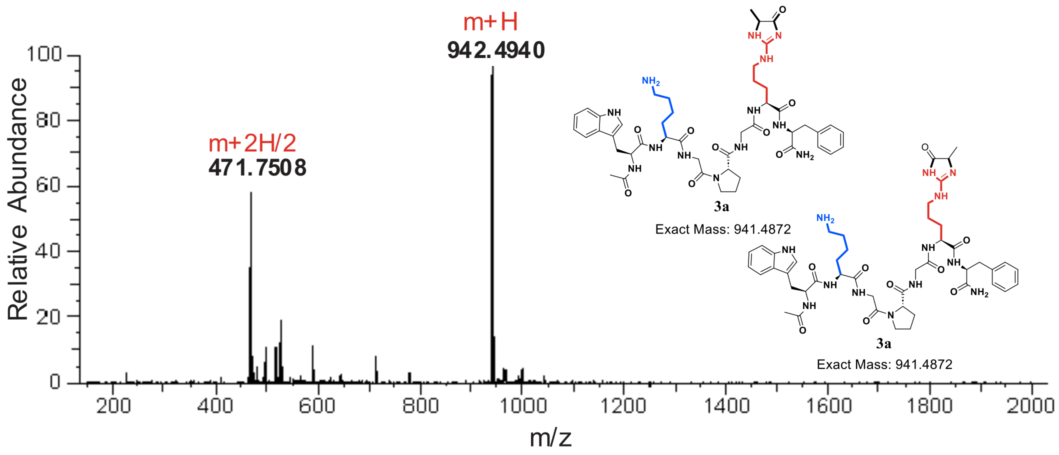

HRMS Trace of Peak at 11.3 min

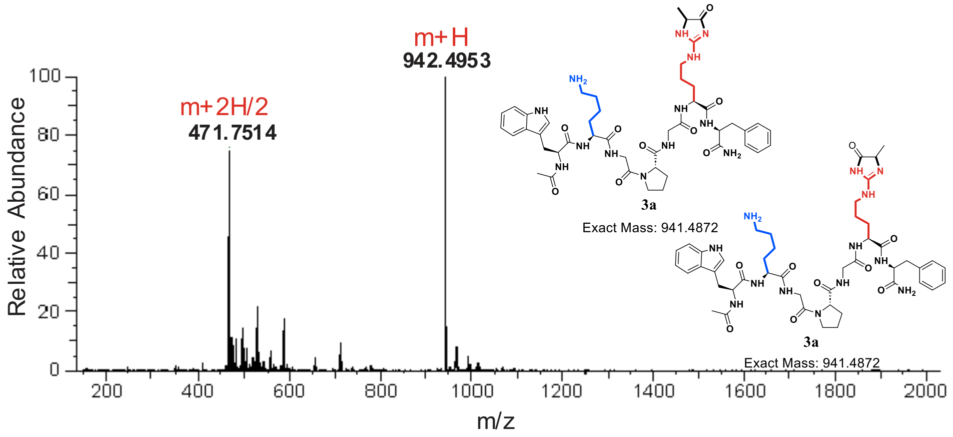

HRMS Trace of Peak at 13.6 and 13.8 min

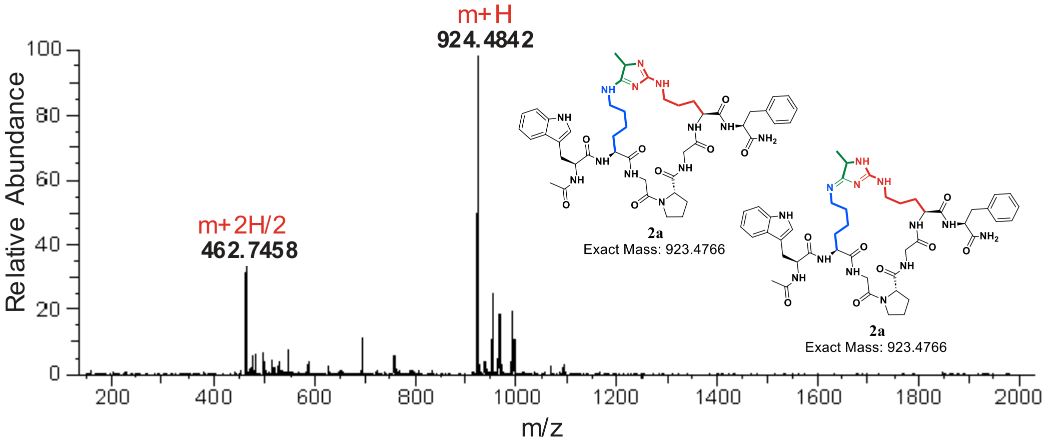

Supplemental Figure 3f. Evaluation of no base

Ac-WKGPGRF-CONH2 ($C_{43}H_{61}N_{13}O_8$) linear peptide 1a. LCMS: *m/z* 888.4838 (calcd $[M+H]^+$ = 888.4839), *m/z* 444.7456 (calcd $[M+2H/2]^+$ = 444.7462) (HPLC analysis at 220 nm). Retention time in HPLC: 10.3 min

HPLC Trace of Entry 6 at 220 nm

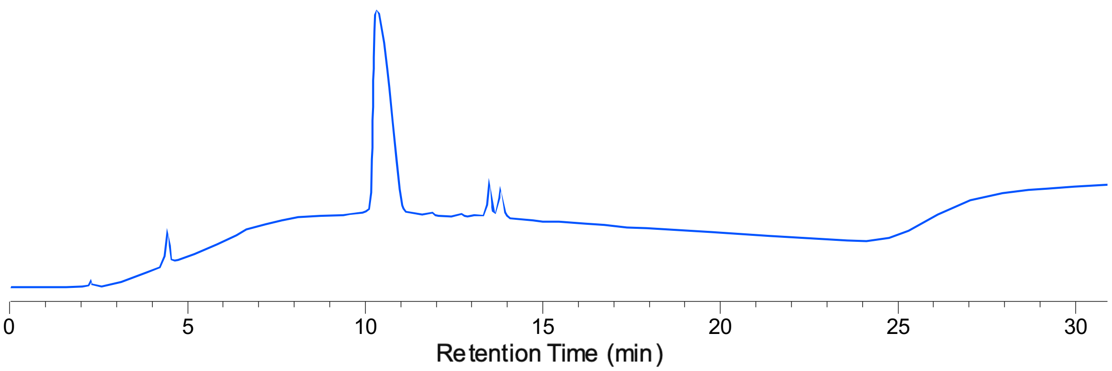

HRMS Trace of Peak at 10.3 min

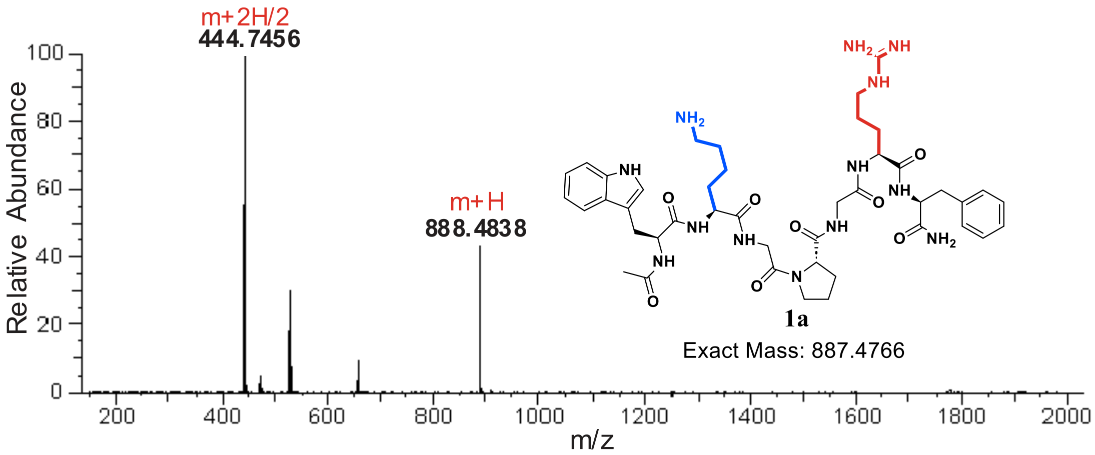

Supplementary Figure 4. Evaluation of Methylglyoxal Equivalences

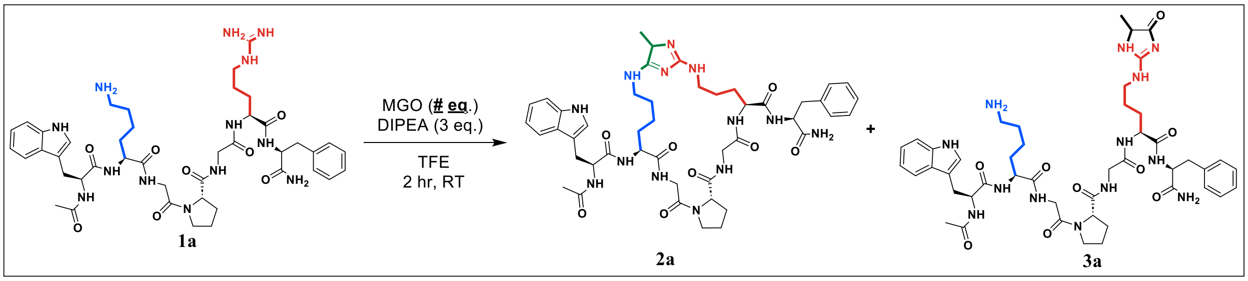

Entry	Equivalence	HPLC Conversion
**1**	**1.2**	**71%**
2	2	64%
3	3	52%
4	5	34%

General Procedure 1. Optimal Reaction Conditions for the Intramolecular Cyclization of Arginine and Lysine Linear peptide **1a** (1.0 mg, 1.0 eq) was dissolved in 250 µL of TFE and the solution was left to stir at room temperature for 5 minutes with DIPEA (3 eq). Methylglyoxal (1.2 eq) was added to the reaction mixture and was left to stir at room temperature for 2 hours. An extended reaction time was required for some peptides. For determining % conversion, an aliquot was taken from the reaction mixture and injected into the HPLC using **HPLC Method A** at detection wavelength 220 nm. The masses of the products were confirmed with LC-MS.

General Procedure 2. Reaction Conditions for the Intermolecular Reaction of Guanidine Hydrochloride and Lysine Peptide (1.0 mg, 1.0 eq) was dissolved in 250 µL of TFE. Methylglyoxal (10 eq) and DIPEA (10 eq) were added simultaneously, and the mixture was left to stir for 20 minutes at room temperature. Guanidine hydrochloride (10 eq) was introduced, and the

reaction mixture was stirred for 2 hours. For determining % conversion, an aliquot was taken from the reaction mixture and injected into the HPLC using **HPLC Method A** at detection wavelength 220 nm. The masses of the products were confirmed with LC-MS.

Supplementary Figure 5a. Cyclization of Optimization Peptide 1a

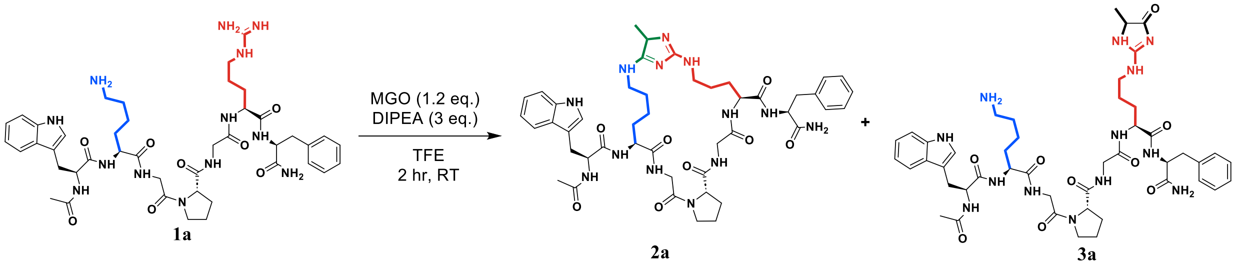

Linear peptide **1a** (1.0 mg, 0.001 mmol, 1.0 eq) was dissolved in 250 µL of TFE and the solution was left to stir at room temperature for 5 minutes with DIPEA (3 eq). Methylglyoxal (1.2 eq) was added to the reaction mixture and was left to stir at room temperature for 2 hours. Samples were taken from the reaction mixture and injected into the HPLC using **HPLC Method A** to determine % conversion. The masses of the products were confirmed with LC-MS and compiled below. The conversion of the cyclized product was determined to be (71%).

Ac-WKGPGRF-CONH$_2$ ($C_{43}H_{61}N_{13}O_8$) linear peptide 1a. LCMS: *m/z* 888.4839 (calcd $[M+H]^+$ = 888.4839) (HPLC analysis at 220 nm). Retention time in HPLC: 10.7 min

Ac-WKGPGRF-CONH$_2$ ($C_{46}H_{63}N_{13}O_9$) arginine adduct products 3a. LCMS: *m/z* 942.4950 (10.9 min) and 942.4946 (11.2 min) (calcd $[M+H]^+$ = 942.4944), *m/z* 471.7512 (10.9 min) and 471.7511 (11.2 min) (calcd $[M+2H/2]^+$ = 471.7514), (HPLC analysis at 220 nm). Retention time in HPLC: 10.9 and 11.2 min. (29%). Two peaks in LC trace indicate the presence of two isomers.

Ac-WKGPGRF-CONH$_2$ ($C_{46}H_{61}N_{13}O_8$) cyclized peptide products 2a. LCMS: *m/z* 924.4843 (13.3 min) and 924.4842 (13.5 min) (calcd $[M+H]^+$ = 924.4839, (HPLC analysis at 220 nm). Retention time in HPLC: 13.3 and 13.5 min. (71%). Two peaks in LC trace indicate the presence of two isomers.

HPLC Trace of Optimization Peptide Ac-WKGPGRF-CONH$_2$ at 220nm

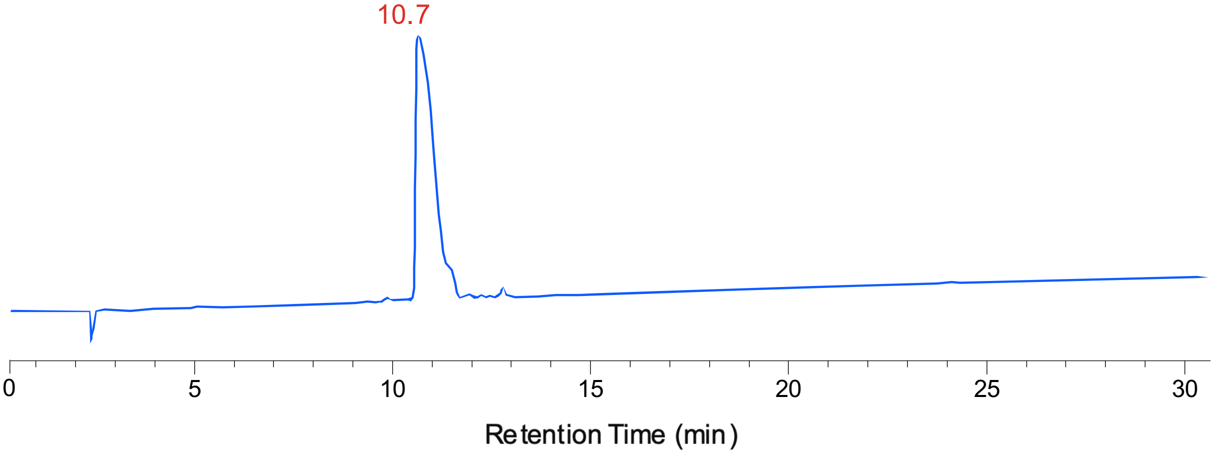

HRMS Trace of Peak at 10.7 min

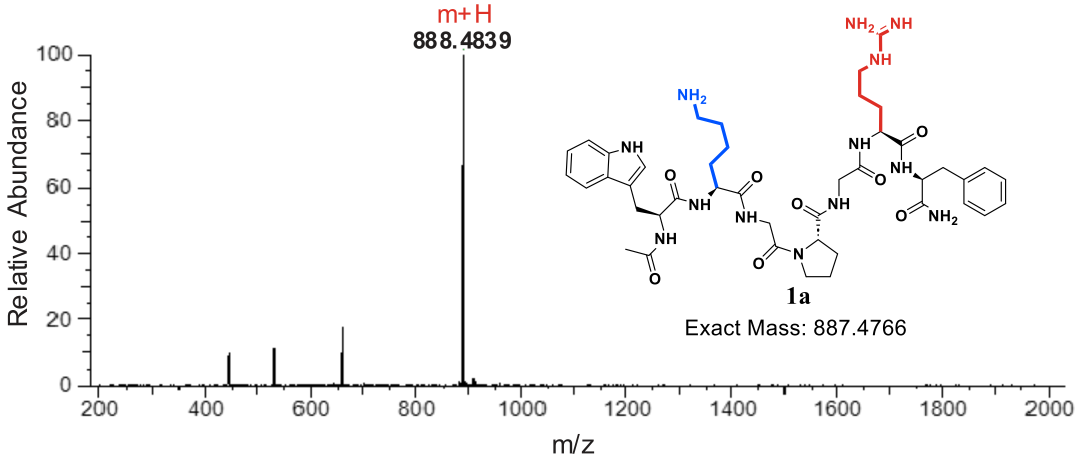

HPLC Trace of Ac-WKGPGRF-CONH₂ Reaction Mixture at 220nm

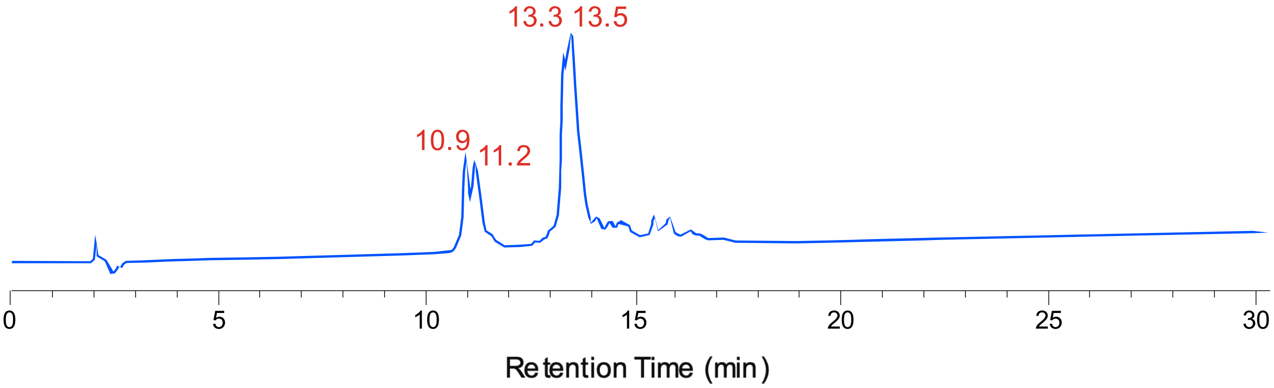

HRMS Trace of Peak at 10.9 min

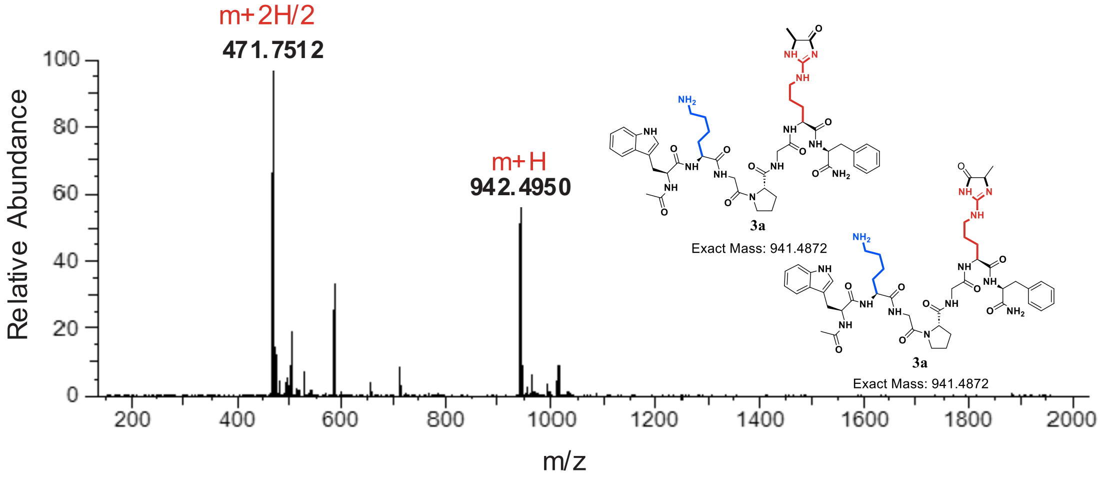

HRMS Trace of Peak at 11.2 min

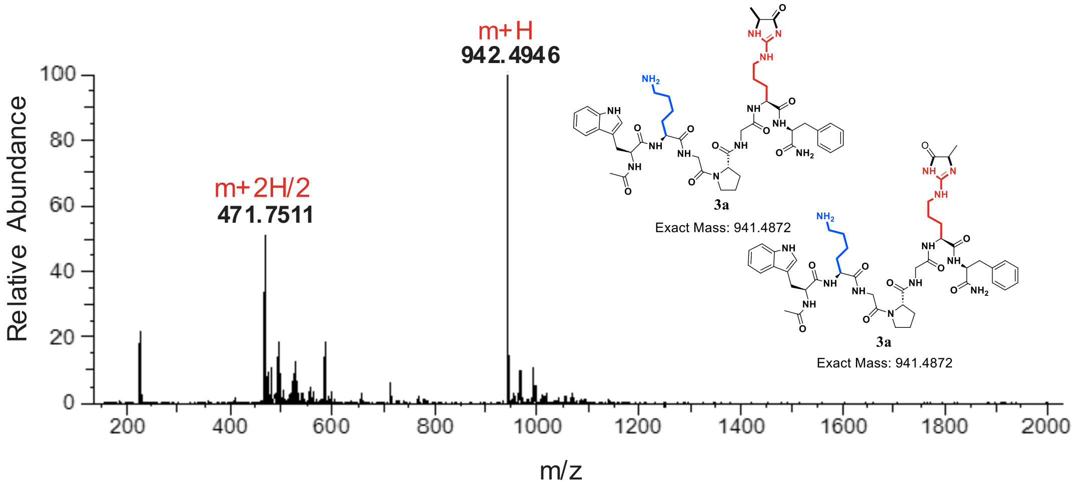

HRMS Trace of Peak at 13.3 and 13.5 min

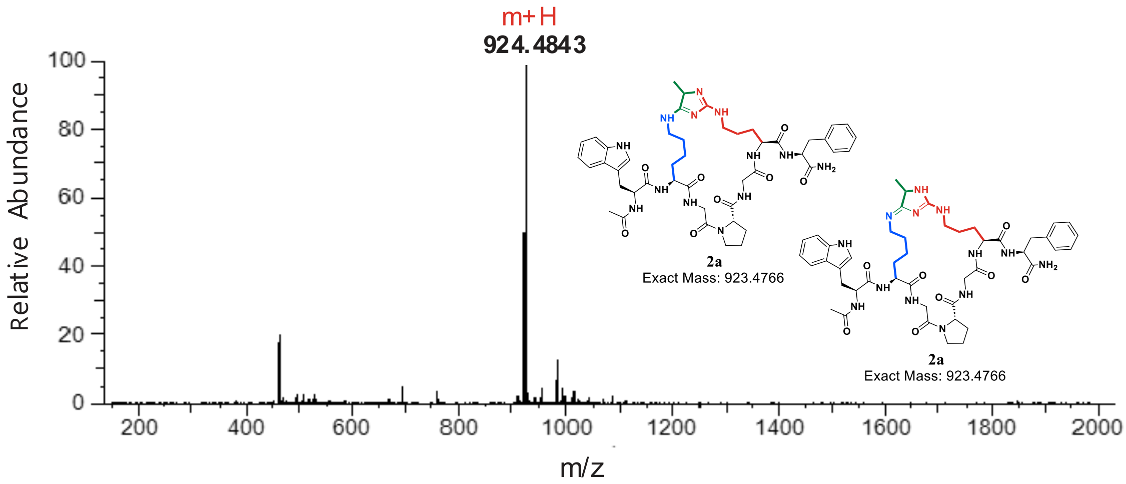

Supplementary Figure 5b. Cyclization of Peptide 1b

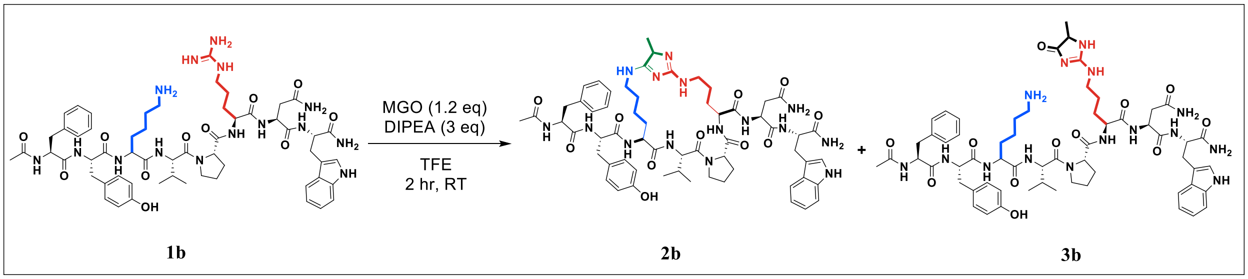

Linear peptide **1b** (1.0 mg, 0.001 mmol, 1.0 eq) was dissolved in 250 µL of TFE and the solution was left to stir at room temperature for 5 minutes with DIPEA (3 eq). Methylglyoxal (1.2 eq) was added to the reaction mixture and was left to stir at room temperature for 2 hours. Samples were taken from the reaction mixture and injected into the HPLC using **HPLC Method A** to determine

% conversion. The masses of the products were confirmed with LC-MS and compiled below. The conversion of the cyclized product was determined to be (81%).

Ac-FYKVPRNW-CONH2 ($C_{57}H_{79}N_{15}O_{11}$) linear peptide 1b. LCMS: *m/z* 1150.7427 (calcd $[M+H]^+$ = 1150.6156), *m/z* 576.1076 (calcd $[M+2H/2]^+$ = 575.8120), (HPLC analysis at 220 nm). Retention time in HPLC: 12.1 min

Ac-FYKVPRNW-CONH2 ($C_{60}H_{81}N_{15}O_{12}$) arginine adduct products 3b. LCMS: 1204.7484 (calcd $[M+H]^+$ = 1204.6264), *m/z* 603.0944 (calcd $[M+2H/2]^+$ = 602.8173) (HPLC analysis at 220 nm). Retention time in HPLC: 12.8 min. (19%)

Ac-FYKVPRNW-CONH2 ($C_{60}H_{79}N_{15}O_{11}$) cyclized peptide products 2b. LCMS: *m/z* 1186.7449 (14.5 min) and 1186.8057 (14.6 min) (calcd $[M+H]^+$ = 1186.6156), (HPLC analysis at 220 nm). Retention time in HPLC: 14.5 and 14.6 min. (81%). Two peaks in LC trace indicate the presence of two isomers.

HPLC Trace of Ac-FYKVPRNW-CONH2 Starting Peptide at 220 nm

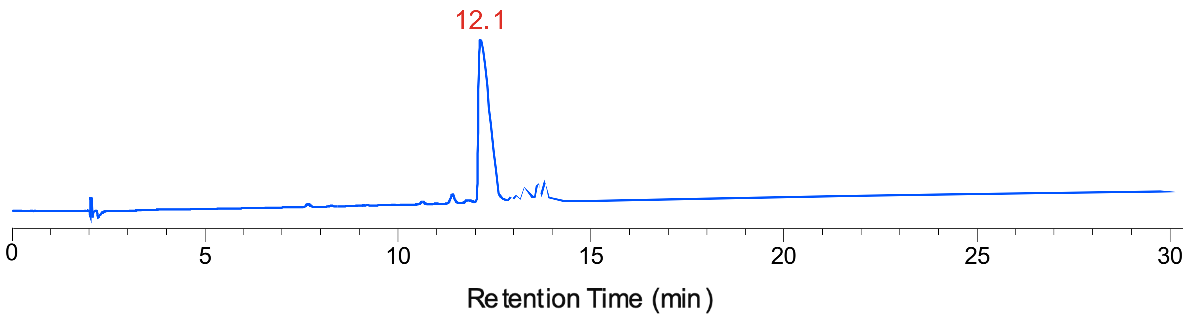

HRMS Trace of Peak at 12.1 min

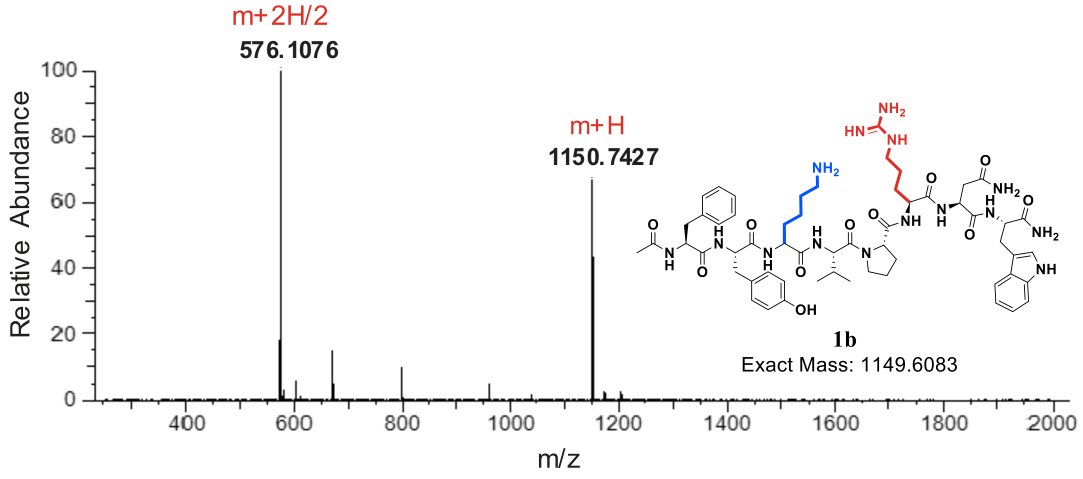

HPLC Trace of Ac-FYKVPRNW-$CONH_2$ Reaction Mixture at 220 nm

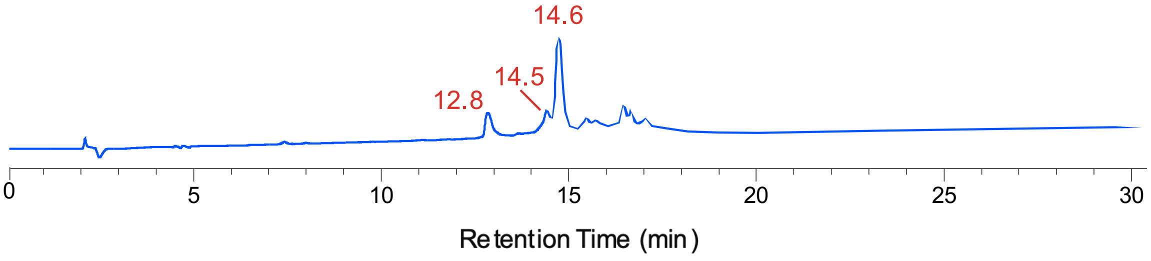

HRMS Trace of Peak at 12.8 min

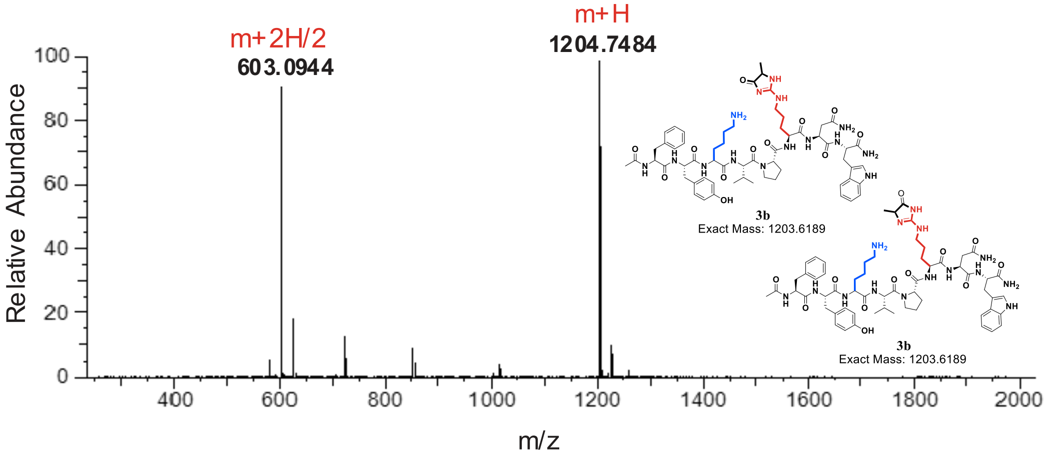

HRMS Trace of Peak at 14.5 and 14.6 min

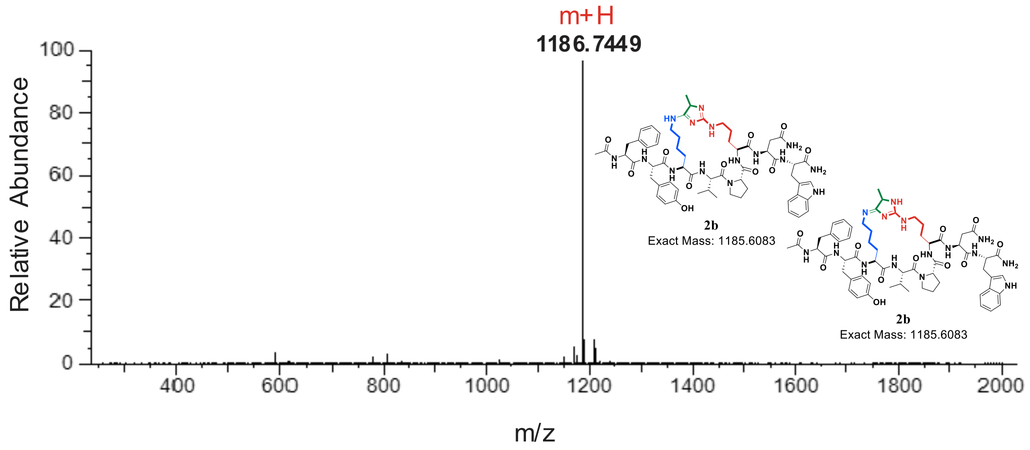

Supplementary Figure 5c. Cyclization of Peptide 1c

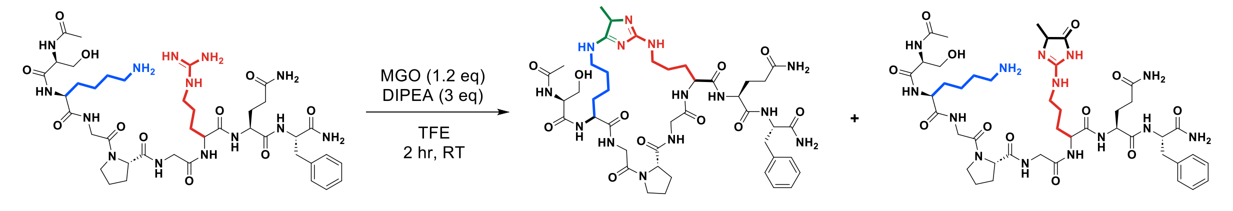

Linear peptide **1c** (1.0 mg, 0.001 mmol, 1.0 eq) was dissolved in 250 µL of TFE and the solution was left to stir at room temperature for 5 minutes with DIPEA (3 eq). Methylglyoxal (1.2 eq) was added to the reaction mixture and was left to stir at room temperature for 2 hours. Samples were taken from the reaction mixture and injected into the HPLC using **HPLC Method A** to determine % conversion. The masses of the products were confirmed with LC-MS and compiled below. The conversion of the cyclized product was determined to be (86%).

Ac-SKGPGRQF-CONH$_2$ ($C_{40}H_{64}N_{14}O_{11}$) linear peptide 1c. LCMS: *m/z* 917.6050 (calcd $[M+H]^+$ = 917.4952), *m/z* 459.5579 (calcd $[M+2H/2]^+$ = 459.2518), (HPLC analysis at 220 nm). Retention time in HPLC: 6.8 min

Ac-SKGPGRQF-CONH$_2$ ($C_{43}H_{66}N_{14}O_{12}$) arginine adduct products 3c. LCMS: 971.6404 (calcd $[M+H]^+$ = 971.5057), *m/z* 486.5918 (calcd $[M+2H/2]^+$ = 486.2571) (HPLC analysis at 220 nm). Retention time in HPLC: 7.3 min. (14%)

Ac-SKGPGRQF-CONH$_2$ ($C_{43}H_{64}N_{14}O_{11}$) cyclized peptide products 2c. LCMS: *m/z* 953.6361 (calcd $[M+H]^+$ = 953.4952), (HPLC analysis at 220 nm). Retention time in HPLC: 9.8 min. (86%)

HPLC Trace of Ac-SKGPGRQF-CONH$_2$ Starting Peptide at 220 nm

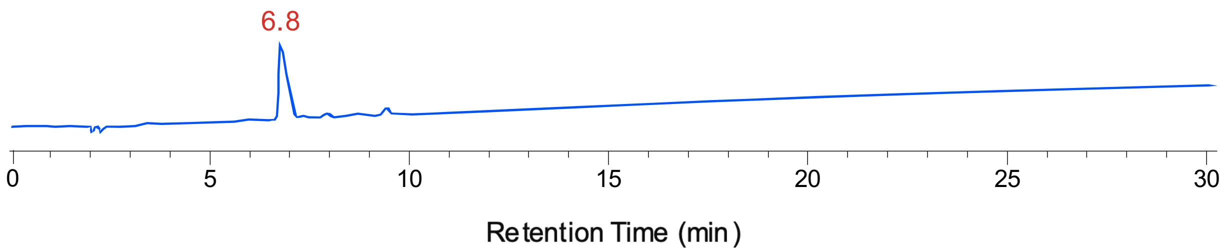

HRMS Trace of Peak at 6.8 min

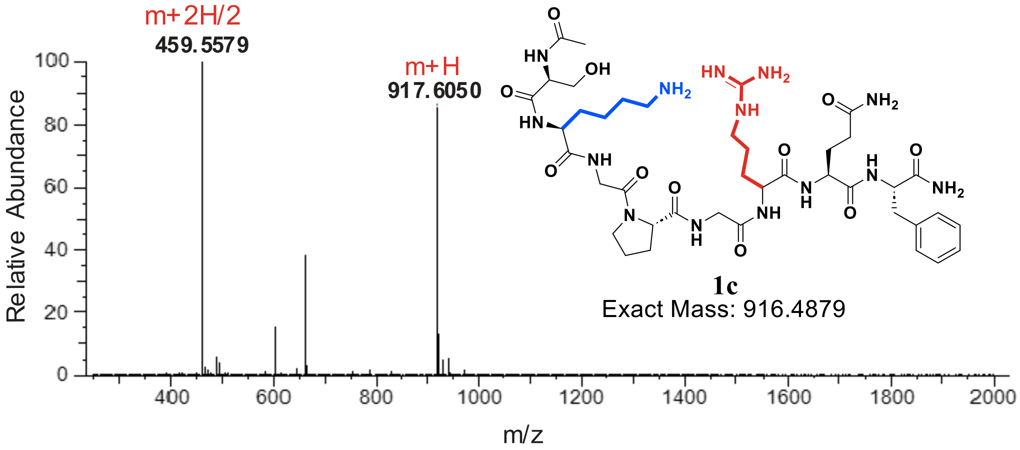

HPLC Trace of Ac-SKGPGRQF-CONH₂ Reaction Mixture at 220 nm

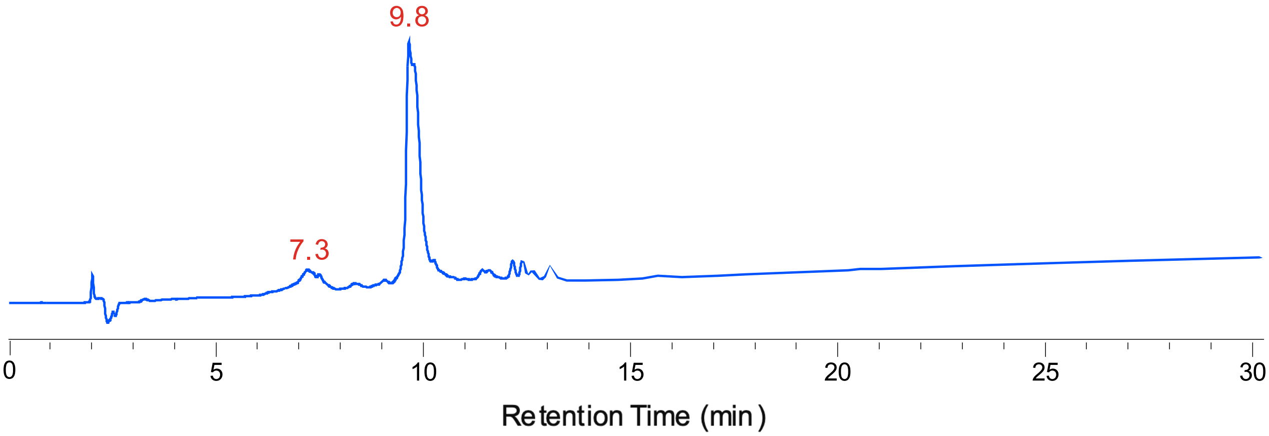

HRMS Trace of Peak at 7.3 min

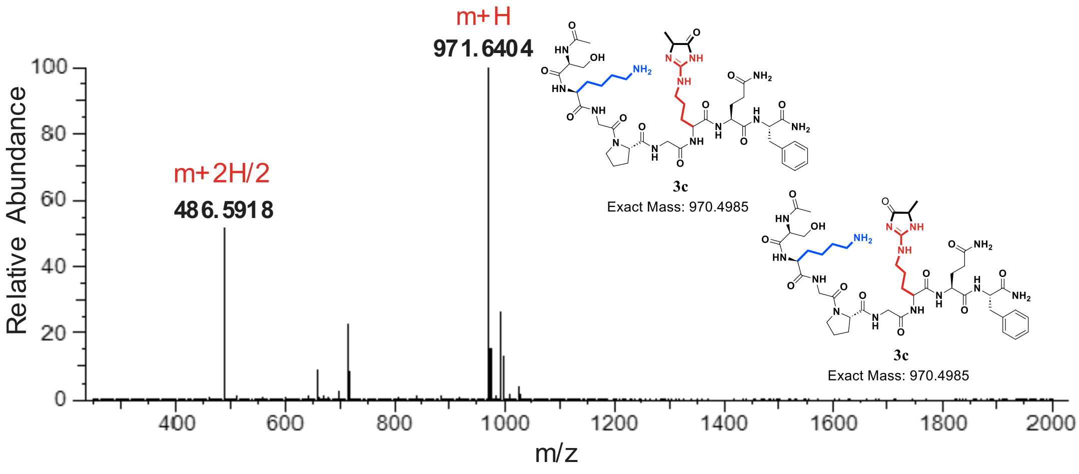

HRMS Trace of Peak at 9.8 min

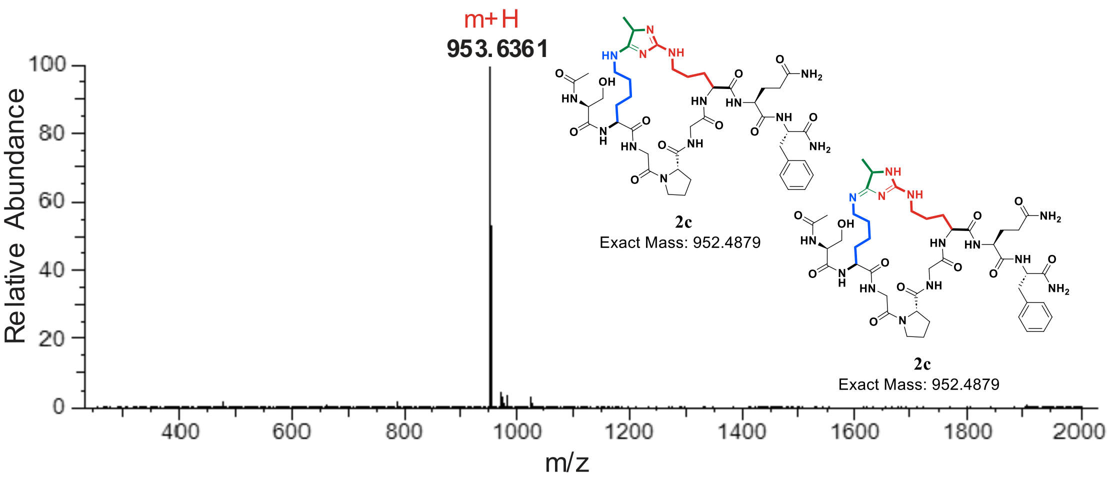

Supplementary Figure 5d. Cyclization of Peptide 1d

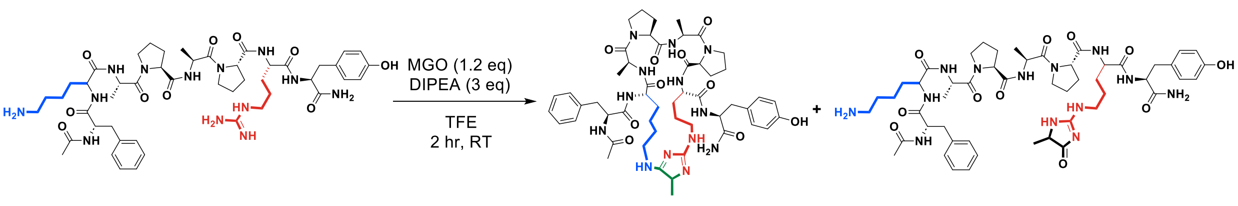

Linear peptide **1d** (1.0 mg, 0.001 mmol, 1.0 eq) was dissolved in 250 µL of TFE and the solution was left to stir at room temperature for 5 minutes with DIPEA (3 eq). Methylglyoxal (1.2 eq) was added to the reaction mixture and was left to stir at room temperature for 2 hours. Samples were taken from the reaction mixture and injected into the HPLC using **HPLC Method A** to determine % conversion. The masses of the products were confirmed with LC-MS and compiled below. The conversion of the cyclized product was determined to be (52%).

Ac-FKAPAPRY-CONH$_2$ ($C_{48}H_{71}N_{13}O_{10}$) linear peptide 1d. LCMS: *m/z* 990.6182 (calcd $[M+H]^+$ = 990.5520), *m/z* 496.0125 (calcd $[M+2H/2]^+$ = 495.7802), (HPLC analysis at 220 nm). Retention time in HPLC: 9.2 min

Ac-FKAPAPRY-CONH$_2$ ($C_{51}H_{73}N_{13}O_{11}$) arginine adduct products 3d. LCMS: 1044.6750 (9.5 min) and 1044.6284 (9.7 min) (calcd $[M+H]^+$ = 1044.5625), *m/z* 523.0707 (9.5 min) (calcd $[M+2H/2]^+$ = 522.7855) (HPLC analysis at 220 nm). Retention time in HPLC: 9.5 and 9.7 min. (48%). Two peaks in LC trace indicate the presence of two isomers.

Ac-FKAPAPRY-CONH$_2$ ($C_{51}H_{71}N_{13}O_{10}$) cyclized peptide products 2d. LCMS: *m/z* 1026.6738 (calcd $[M+H]^+$ = 1026.5520), (HPLC analysis at 220 nm). Retention time in HPLC: 12.2 min. (52%)

HPLC Trace of Ac-FKAPAPRY-CONH$_2$ Starting Peptide at 220 nm

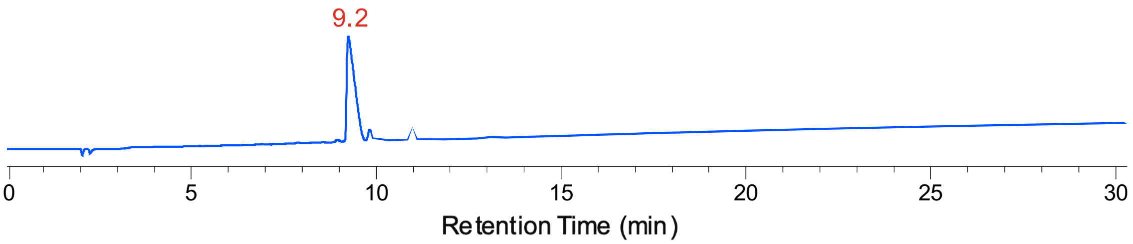

HRMS Trace of Peak at 9.2 min

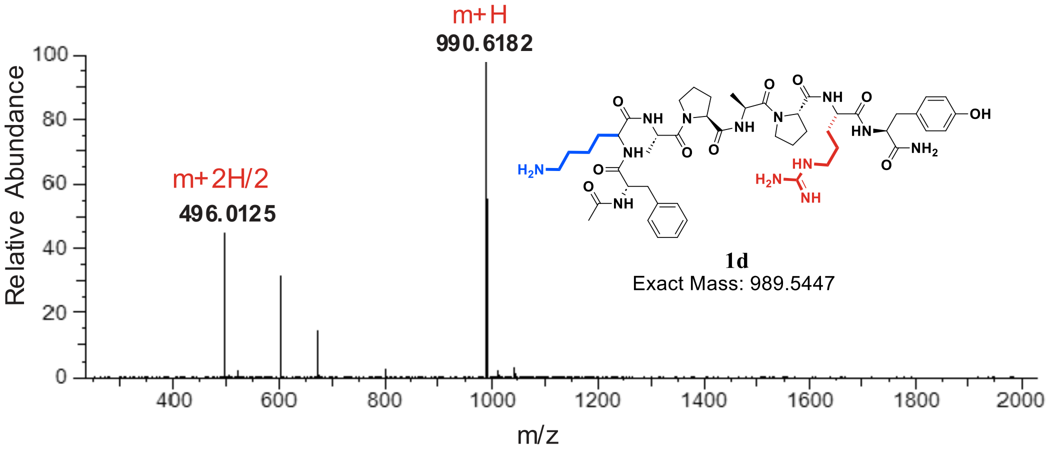

HPLC Trace of Ac-FKAPAPRY-CONH$_2$ Reaction Mixture at 220 nm

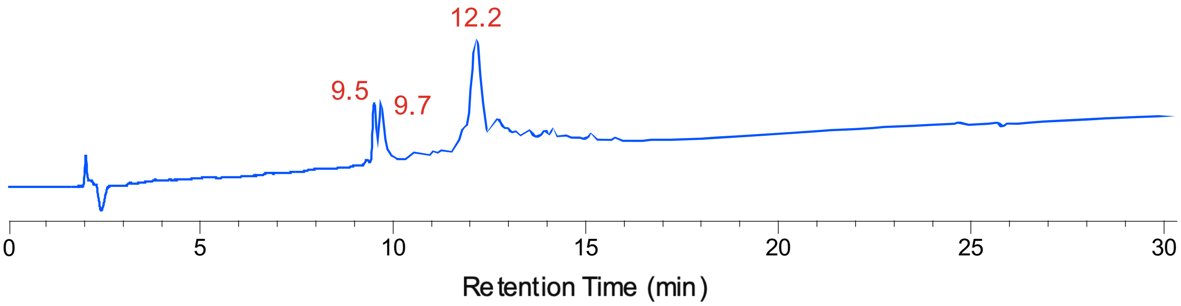

HRMS Trace of Peak at 9.5 min

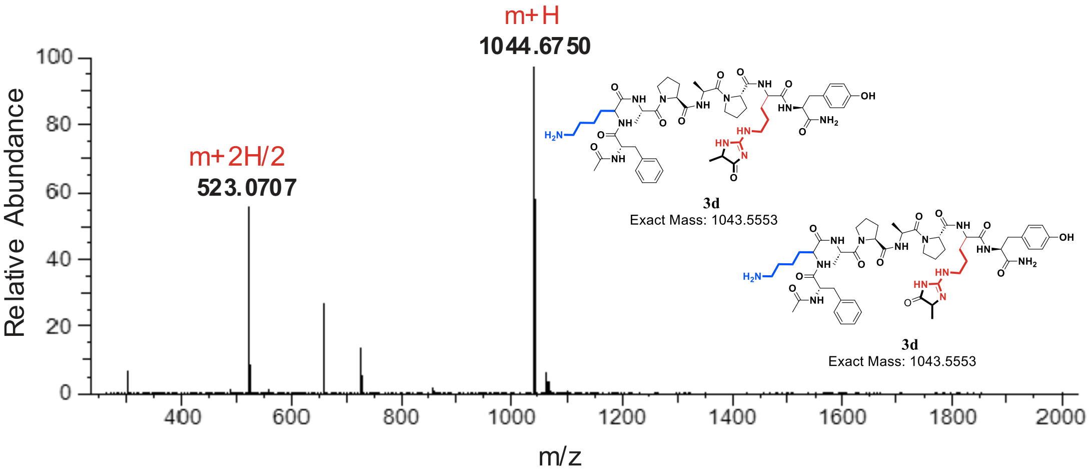

HRMS Trace of Peak at 9.7 min

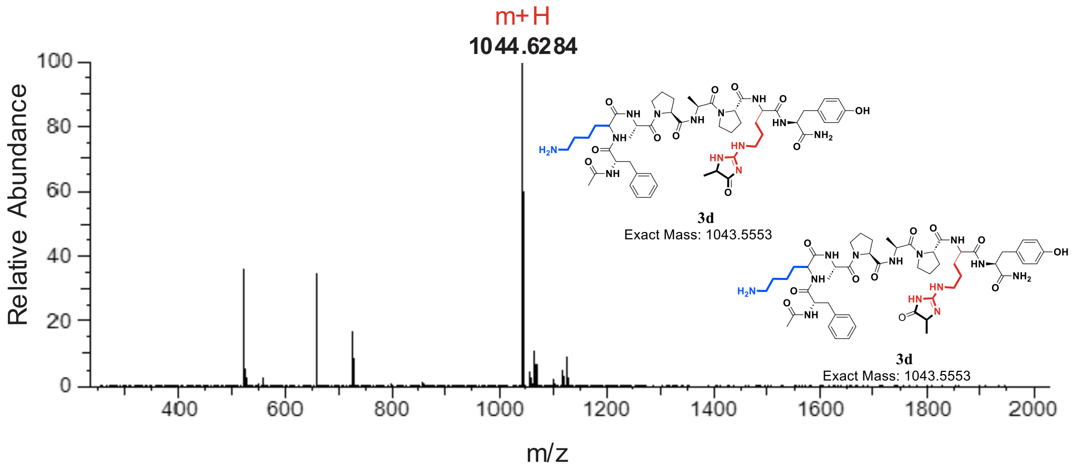

HRMS Trace of Peak at 12.2 min

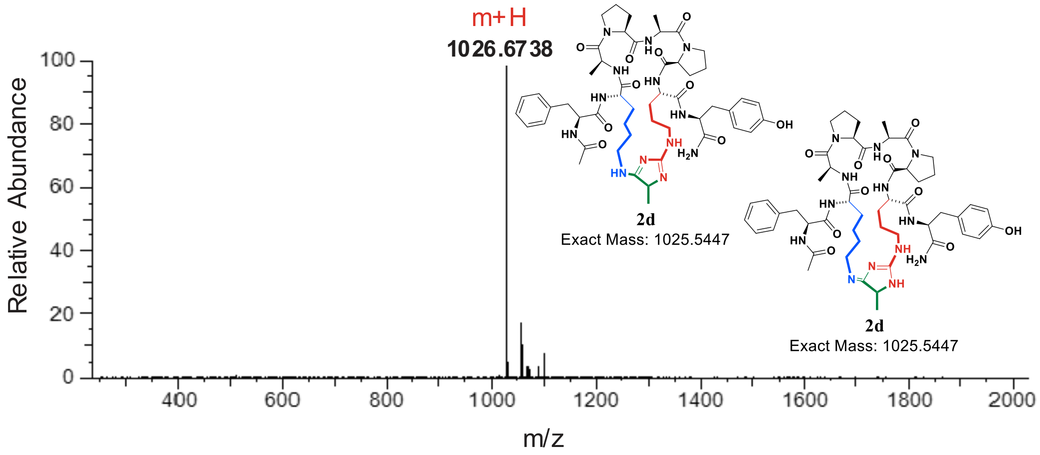

Supplementary Figure 5e. Cyclization of Peptide 1e

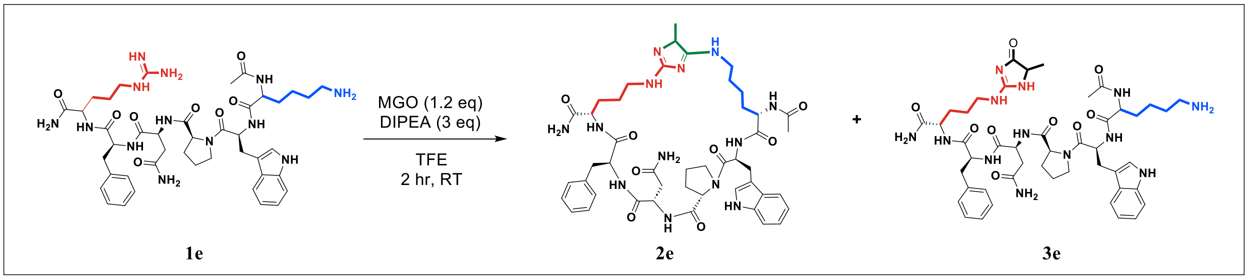

Linear peptide **1e** (1.0 mg, 0.001 mmol, 1.0 eq) was dissolved in 250 µL of TFE and the solution was left to stir at room temperature for 5 minutes with DIPEA (3 eq). Methylglyoxal (1.2 eq) was added to the reaction mixture and was left to stir at room temperature for 3-4 hours. Samples were taken from the reaction mixture and injected into the HPLC using **HPLC Method A** to determine % conversion. The masses of the products were confirmed with LC-MS and compiled below. The conversion of the cyclized product was determined to be (67%).

Ac-KWPNFR-CONH$_2$ ($C_{43}H_{61}N_{13}O_8$) linear peptide 1e. LCMS: *m/z* 888.4865 (calcd $[M+H]^+$ = 888.4839), *m/z* 444.7467 (calcd $[M+2H/2]^+$ = 444.7456), (HPLC analysis at 220 nm). Retention time in HPLC: 10.6 min

Ac-KWPNFR-CONH$_2$ ($C_{46}H_{63}N_{13}O_9$) arginine adduct products 3e. LCMS: *m/z* 942.4946 (11.1 min) and 942.4944 (11.3 min) (calcd $[M+H]^+$ = 942.4944), (HPLC analysis at 220 nm). Retention time in HPLC: 11.1 and 11.3 min. (33%). Two peaks in LC trace indicate the presence of two isomers.

Ac-KWPNFR-CONH$_2$ ($C_{46}H_{61}N_{13}O_8$) cyclized peptide products 2e. LCMS: *m/z* 924.4838 (13.1 min) and 924.4833 (13.6 min) (calcd $[M+H]^+$ = 924.4839), 462.7454 (13.6 min) (calcd $[M+2H/2]^+$ = 462.7462) (HPLC analysis at 220 nm). Retention time in HPLC: 13.1 and 13.6 min. (67%). Two peaks in LC trace indicate the presence of two isomers.

HPLC Trace of Ac-KWPNFR-CONH$_2$ Starting Peptide at 220 nm

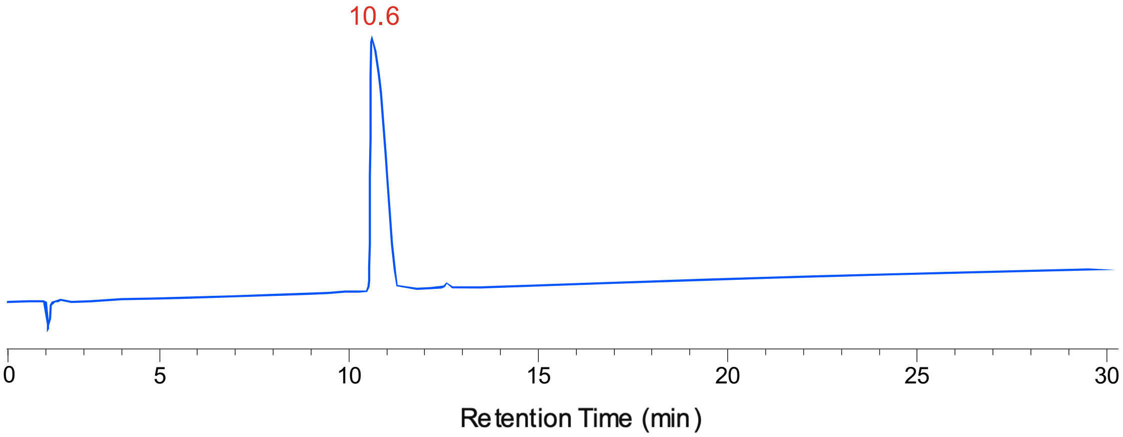

HRMS Trace of Peak at 10.6 min

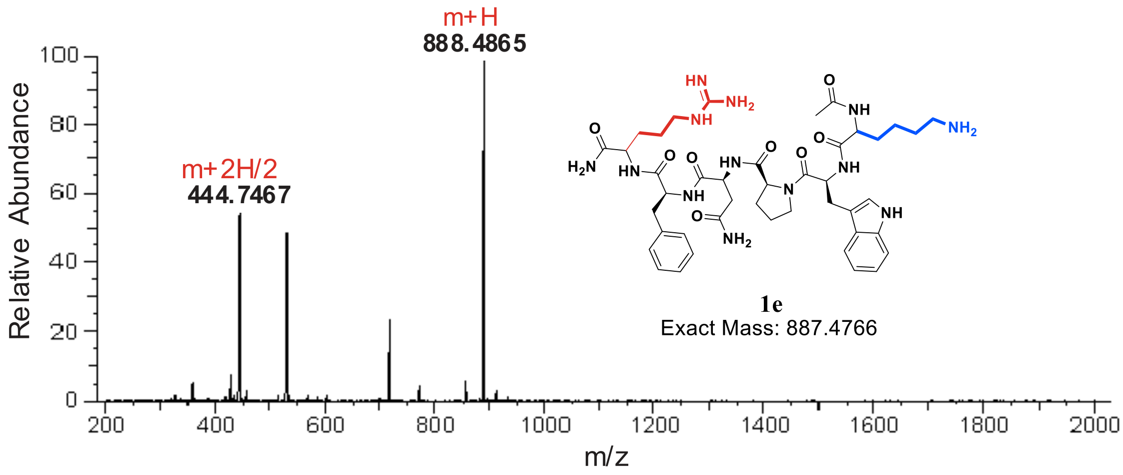

HPLC Trace of Ac-KWPNFR-CONH₂ Reaction Mixture at 220 nm

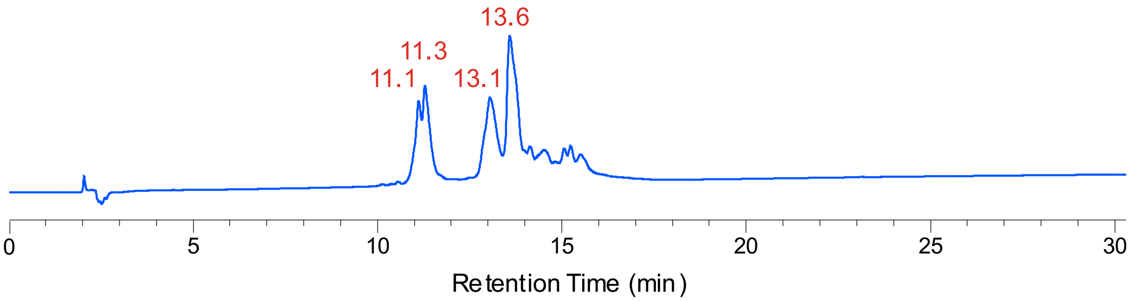

HRMS Trace of Peak at 11.1 min

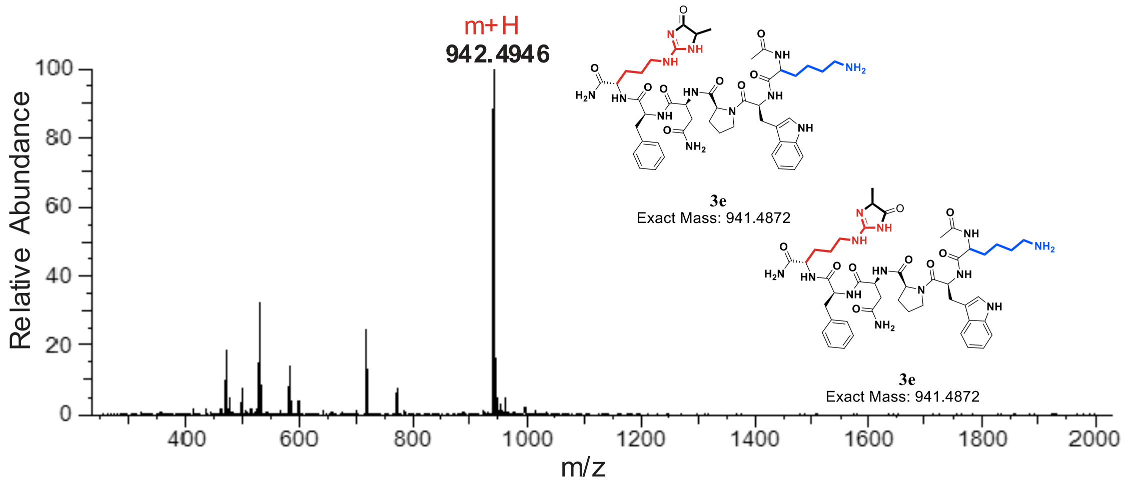

HRMS Trace of Peak at 11.3 min

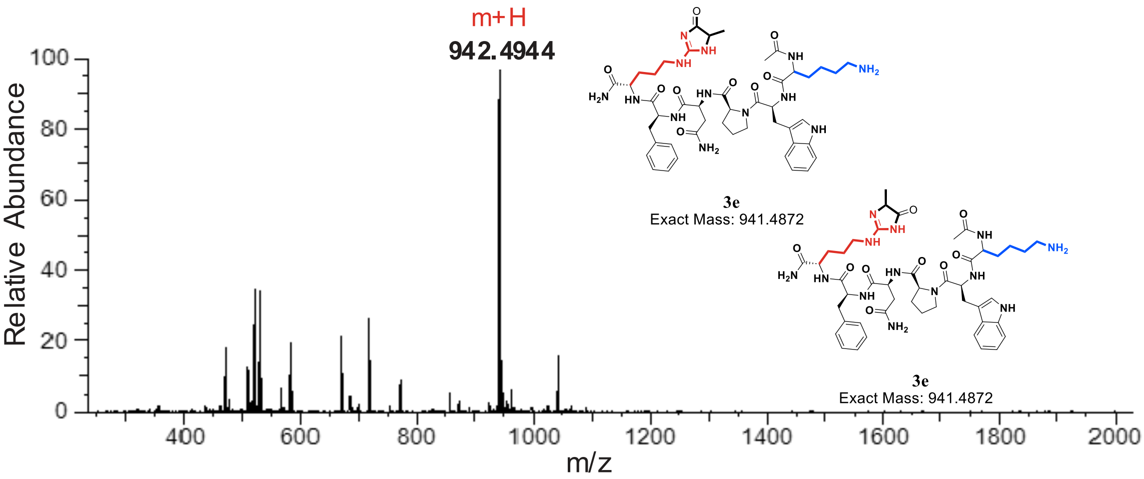

HRMS Trace of Peak at 13.1 min

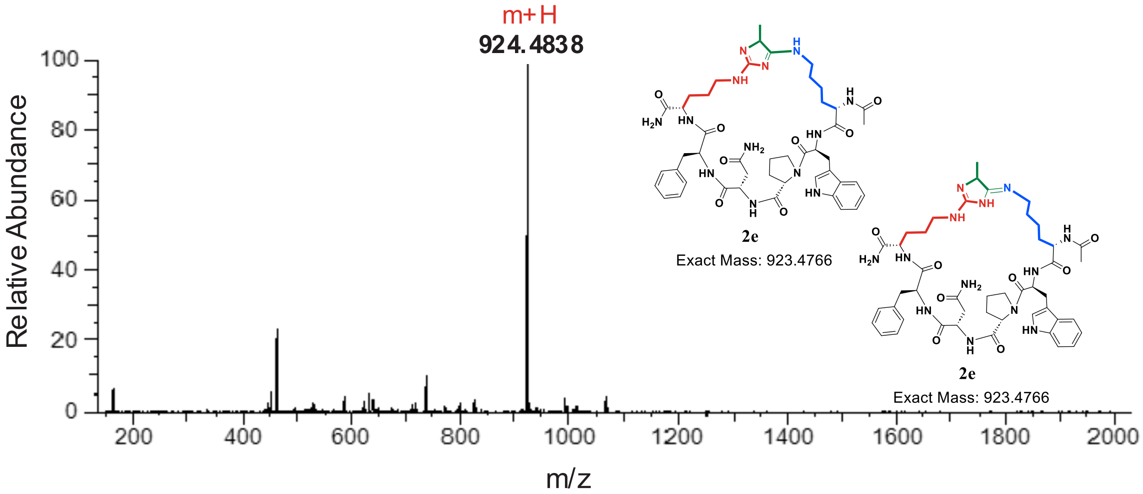

Supplementary Figure 5f. Cyclization of Peptide 1f

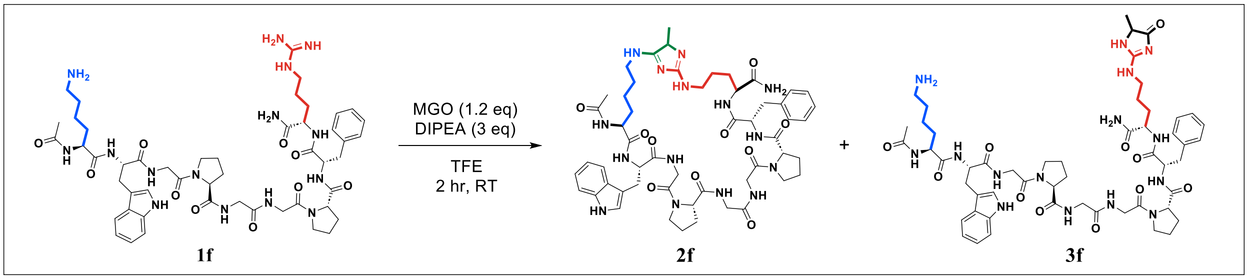

Linear peptide **1f** (1.0 mg, 0.001 mmol, 1.0 eq) was dissolved in 250 µL of TFE and the solution was left to stir at room temperature for 5 minutes with DIPEA (3 eq). Methylglyoxal (1.2 eq) was added to the reaction mixture and was left to stir at room temperature for 4-5 hours. Samples were taken from the reaction mixture and injected into the HPLC using **HPLC Method A** to determine

% conversion. The masses of the products were confirmed with LC-MS and compiled below. The conversion of the cyclized product was determined to be (47%).

Ac-KWGPGGPFR-CONH$_2$ ($C_{50}H_{71}N_{15}O_{10}$) linear peptide 1f. LCMS: *m/z* 1042.6290 (calcd $[M+H]^+$ = 1042.5581), *m/z* 522.0375 (calcd $[M+2H/2]^+$ = 521.7833), (HPLC analysis at 220 nm). Retention time in HPLC: 10.4 min

Ac-KWGPGGPFR-CONH$_2$ ($C_{53}H_{73}N_{15}O_{11}$) arginine adduct products 3f. LCMS: 1096.6120 (calcd $[M+H]^+$ = 1096.5687), *m/z* 549.0059 (calcd $[M+2H/2]^+$ = 548.7885) (HPLC analysis at 220 nm). Retention time in HPLC: 11.1 min. (53%)

Ac-KWGPGGPFR-CONH$_2$ ($C_{53}H_{71}N_{15}O_{10}$) cyclized peptide products 2f. LCMS: *m/z* 1078.6648 (calcd $[M+H]^+$ = 1078.5581), (HPLC analysis at 220 nm). Retention time in HPLC: 13.6 min. (47%)

HPLC Trace of Ac-KWGPGGPFR-CONH$_2$ Starting Peptide at 220 nm

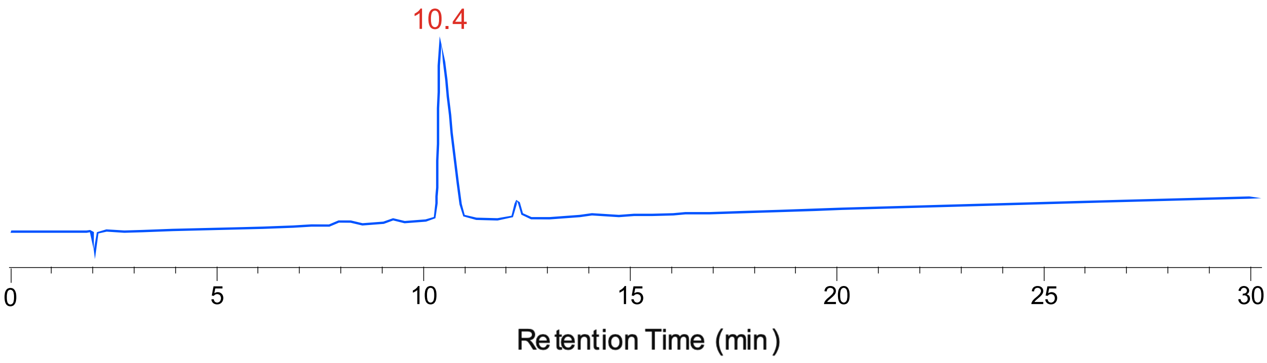

HRMS Trace of Peak at 10.4 min

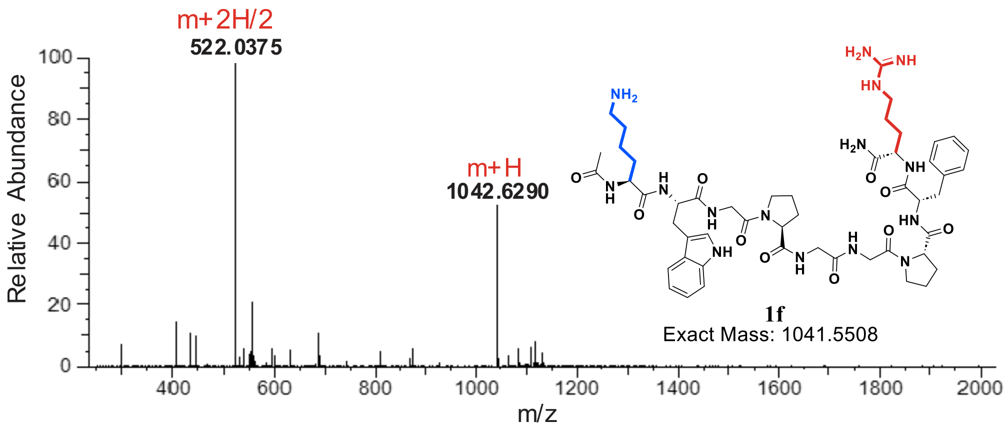

HPLC Trace of Ac-KWGPGGPFR-CONH$_2$ Reaction Mixture at 220 nm

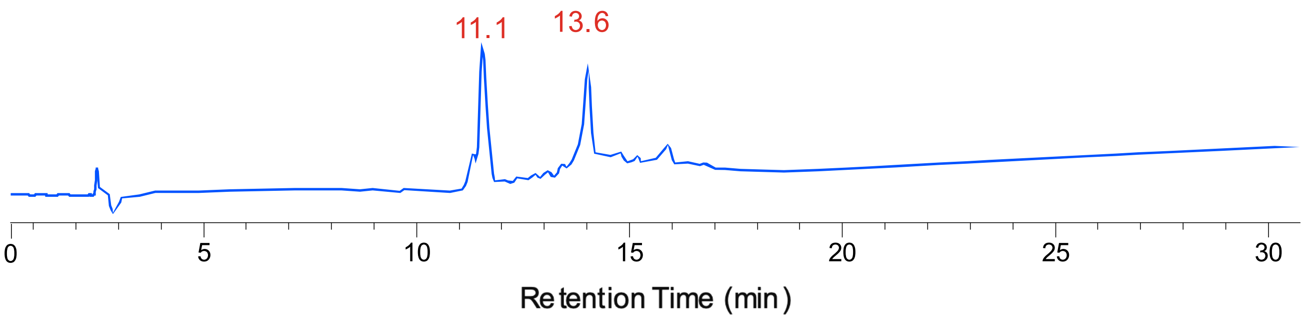

HRMS Trace of Peak at 11.1 min

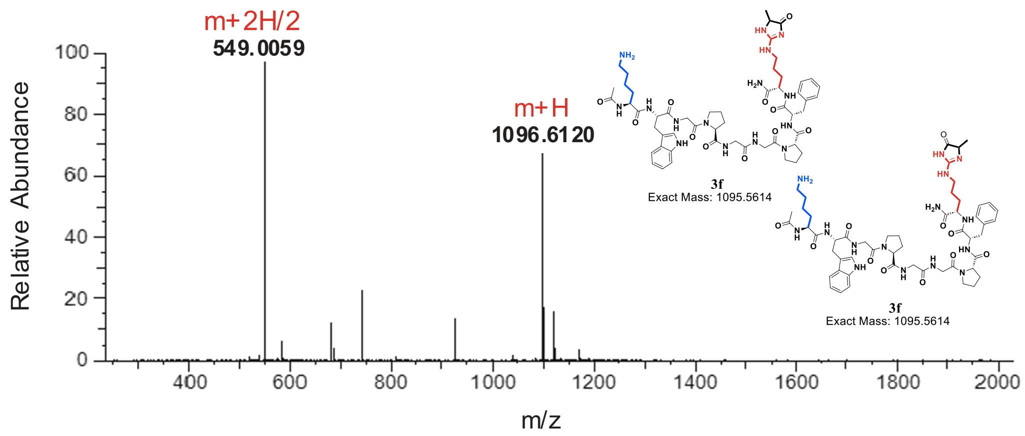

HRMS Trace of Peak at 13.6 min

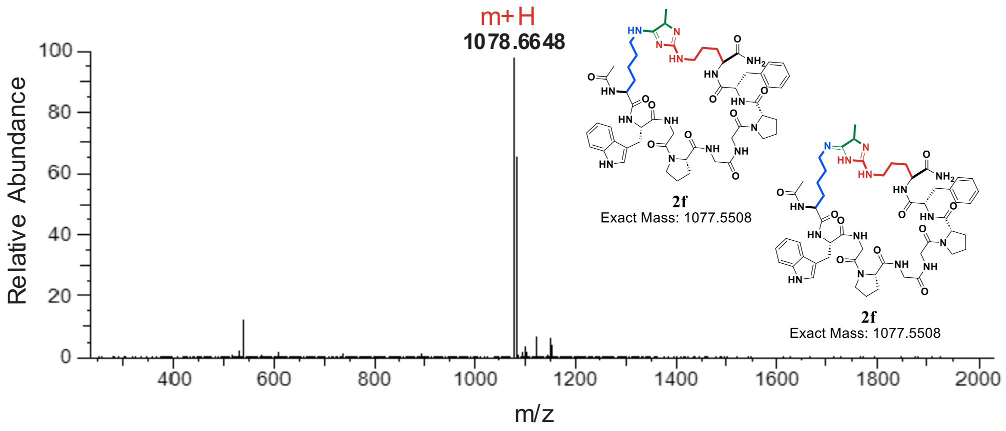

Supplementary Figure 5g. Cyclization of Peptide 1g

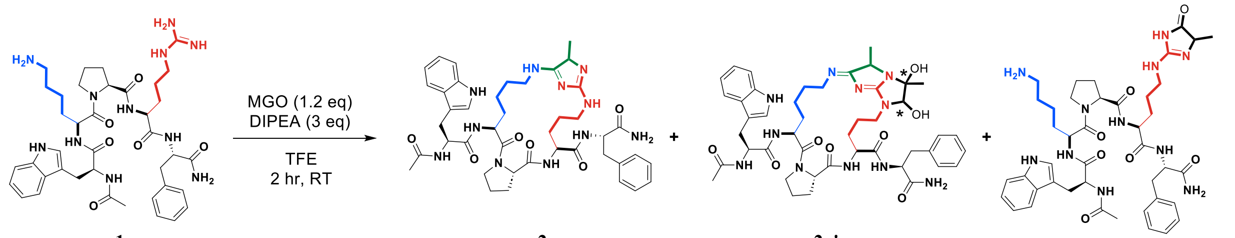

1g **2g** **2g'** **3g**

Linear peptide **1g** (1.0 mg, 0.001 mmol, 1.0 eq) was dissolved in 250 µL of TFE and the solution was left to stir at room temperature for 5 minutes with DIPEA (3 eq). Methylglyoxal (1.2 eq) was added to the reaction mixture and was left to stir at room temperature for 2 hours. Samples were taken from the reaction mixture and injected into the HPLC using **HPLC Method A** to determine % conversion. The masses of the products were confirmed with LC-MS and compiled below. The conversion of the cyclized products was determined to be (75%).

Ac-WKPRF-CONH$_2$ ($C_{39}H_{55}N_{11}O_6$) linear peptide 1g. LCMS: *m/z* 774.6023 (calcd $[M+H]^+$ = 774.4410), *m/z* 388.1309 (calcd $[M+2H/2]^+$ = 387.7247), (HPLC analysis at 220 nm). Retention time in HPLC: 10.2 min

Reaction Mixture:

Ac-WKPRF-CONH$_2$ ($C_{39}H_{55}N_{11}O_6$) linear peptide 1g. LCMS: *m/z* 774.5636 (calcd $[M+H]^+$ = 774.4410), *m/z* 388.0278 (calcd $[M+2H/2]^+$ = 387.7247), (HPLC analysis at 220 nm). Retention time in HPLC: 10.9 min (13%)

Ac-WKPRF-CONH$_2$ ($C_{42}H_{57}N_{11}O_7$) arginine adduct products 3g. LCMS: 828.6152 *m/z* (11.0 min) and 828.5870 (11.3 min) (calcd $[M+H]^+$ = 828.4515), *m/z* 415.1734 (calcd $[M+2H/2]^+$ = 414.7300) (HPLC analysis at 220 nm). Retention time in HPLC: 11.0 and 11.3 min (12%). Two peaks in LC trace indicate the presence of two isomers.

Ac-WKPRF-CONH$_2$ ($C_{42}H_{55}N_{11}O_6$) cyclized peptide products 2g. LCMS: *m/z* 810.5718 (13.1 min) and 810.5549 (13.2 min) (calcd $[M+H]^+$ = 810.4410), (HPLC analysis at 220 nm). Retention time in HPLC: 13.1 and 13.2 min (36%). Two peaks in LC trace indicate the presence of two isomers.

Ac-WKPRF-CONH$_2$ ($C_{45}H_{59}N_{11}O_8$) double MGO addition cyclized products 2g'. LCMS: *m/z* 882.5400 (13.5 min) and 882.5667 (13.7 min) (calcd $[M+H]^+$ = 882.4621), (HPLC analysis at 220 nm). Retention time in HPLC: 13.5 and 13.7 min (39%). Two peaks in LC trace indicate the presence of two isomers.

HPLC Trace of Ac-WKPRF-CONH2 Reaction Mixture at 220 nm

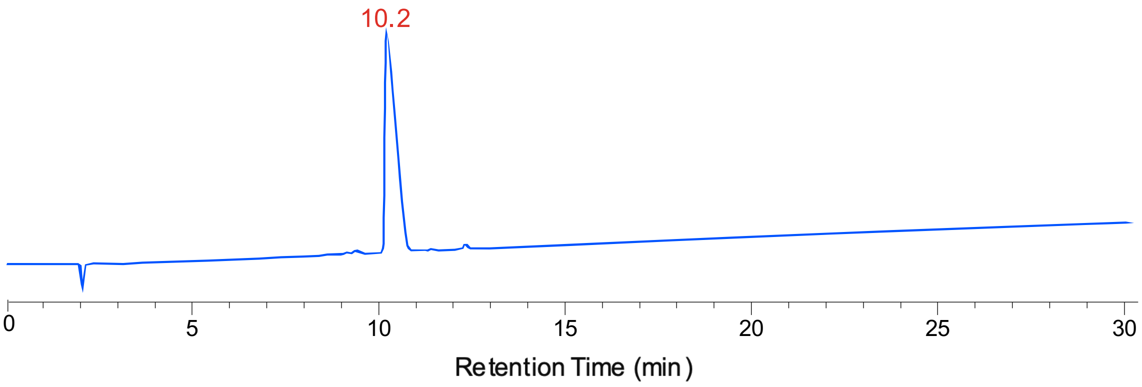

HRMS Trace of Peak at 10.2 min

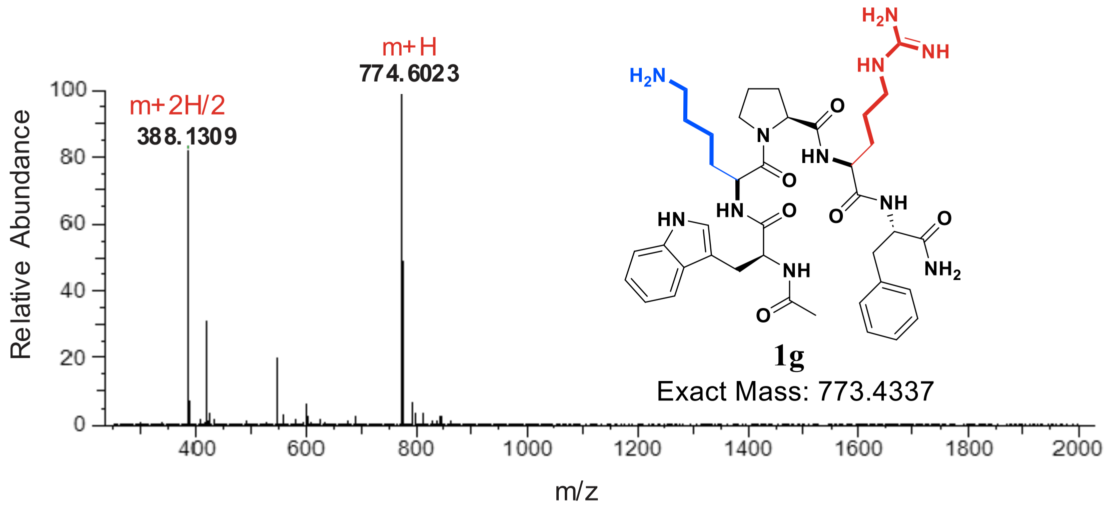

HPLC Trace of Ac-WKPRF-CONH2 Reaction Mixture at 220 nm

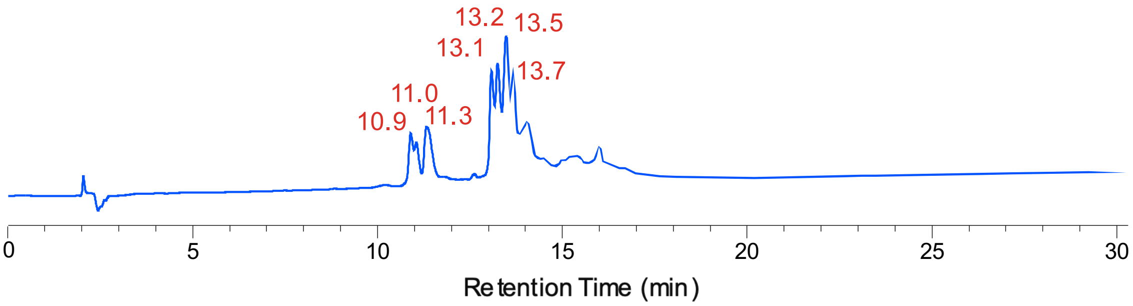

HRMS Trace of Peak at 10.9 min

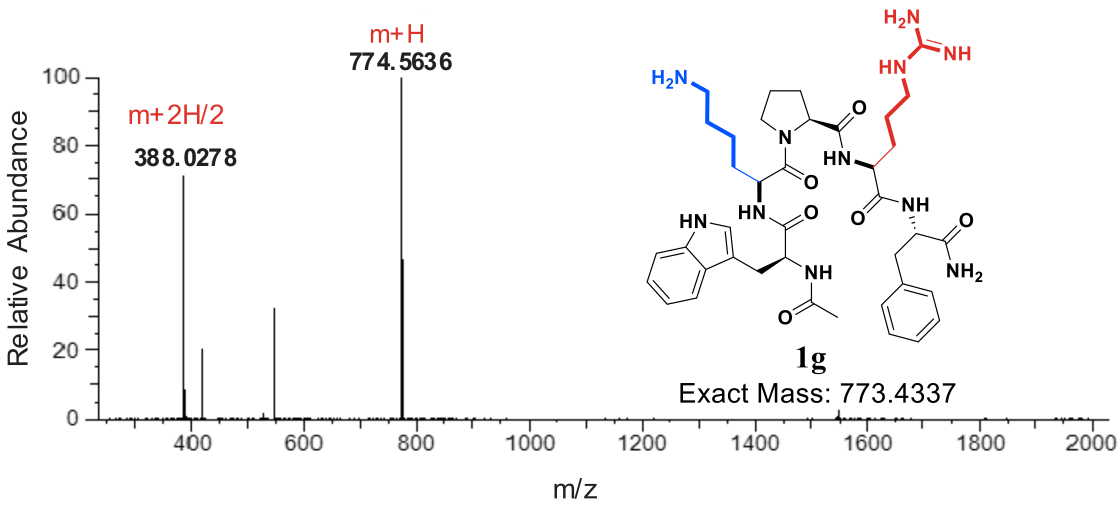

HRMS Trace of Peak at 11.0 min

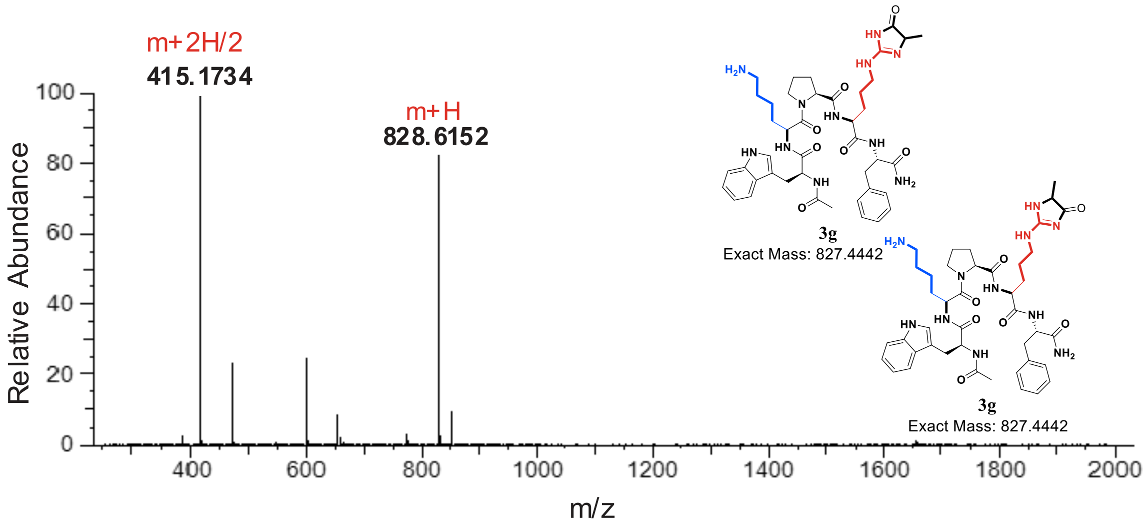

HRMS Trace of Peak at 11.3 min

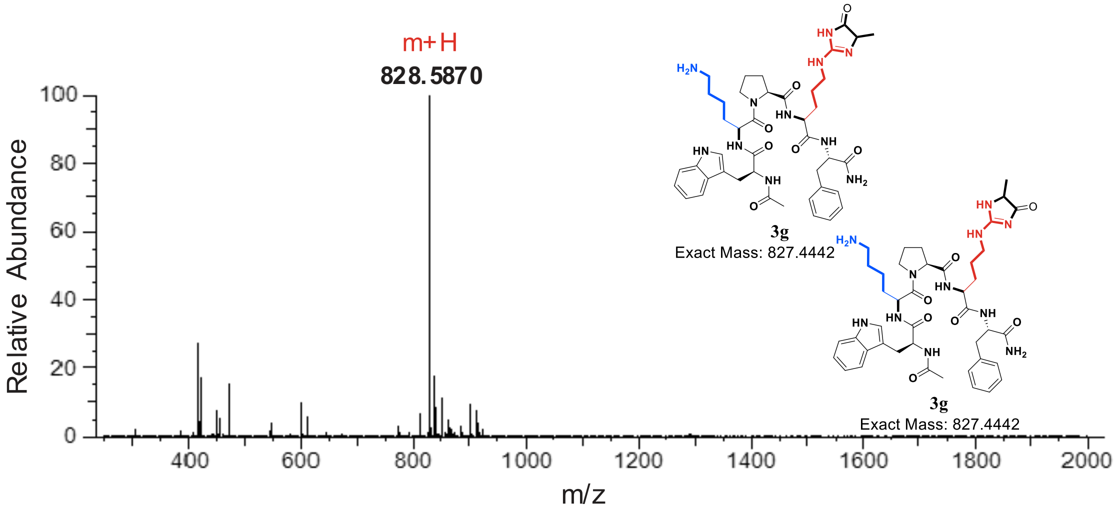

HRMS Trace of Peak 13.1 and 13.2 min

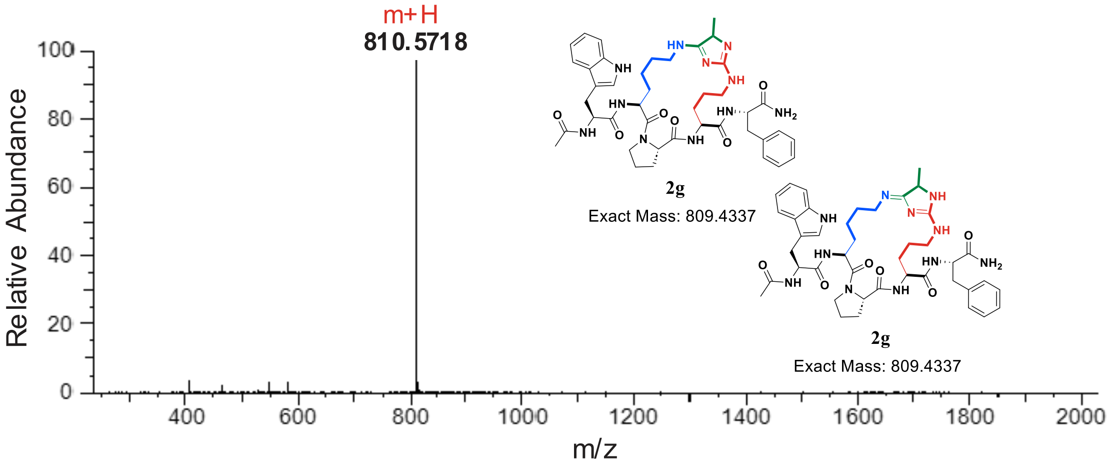

HRMS Trace of Peak at 13.5 and 13.7 min

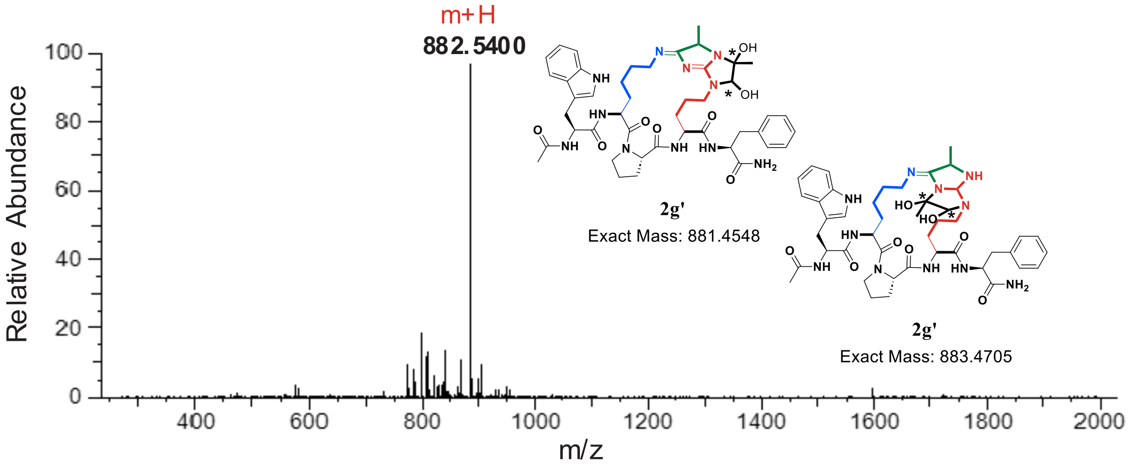

Supplementary Figure 5h. Cyclization of Peptide 1h

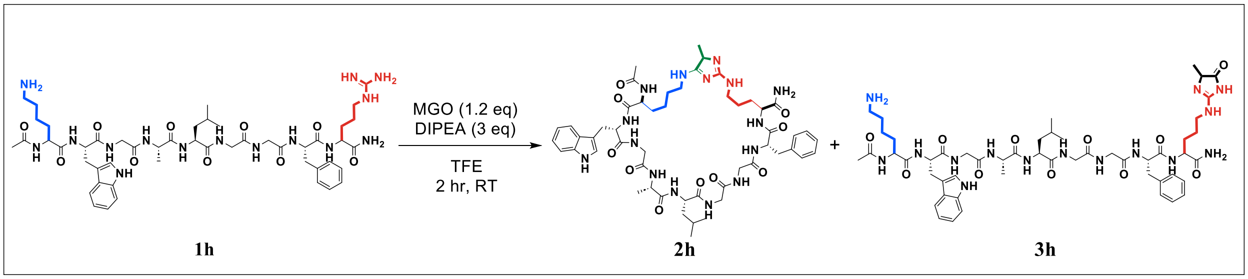

Linear peptide **1h** (1.0 mg, 0.001 mmol, 1.0 eq) was dissolved in 250 μL of TFE and the solution was left to stir at room temperature for 5 minutes with DIPEA (3 eq). Methylglyoxal (1.2 eq) was added to the reaction mixture and was left to stir at room temperature for 4-5 hours. Samples were taken from the reaction mixture and injected into the HPLC using **HPLC Method A** to determine

% conversion. The masses of the products were confirmed with LC-MS and compiled below. The conversion of the cyclized product was determined to be (48%).

Ac-KWGALGGFR-CONH$_2$ ($C_{49}H_{73}N_{15}O_{10}$) linear peptide 1h. LCMS: *m/z* 1032.6710 (calcd $[M+H]^+$ = 1032.5738), *m/z* 517.0794 (calcd $[M+2H/2]^+$ = 516.7911), (HPLC analysis at 220 nm). Retention time in HPLC: 11.7 min

Reaction Mixture:

Ac- KWGALGGFR -CONH$_2$ ($C_{49}H_{73}N_{15}O_{10}$) linear peptide 1h. LCMS: *m/z* 1032.6536 (calcd $[M+H]^+$ = 1032.5738), *m/z* 517.1092 (calcd $[M+2H/2]^+$ = 516.7911) (HPLC analysis at 220 nm). Retention time in HPLC: 11.9 min (24%)

Ac- KWGALGGFR -CONH$_2$ ($C_{52}H_{75}N_{15}O_{11}$) arginine adduct products 3h. LCMS: 1086.6710 (12.1 min) and 1086.6765 (12.2 min) (calcd $[M+H]^+$ = 1086.5843), *m/z* 544.0891 (12.1 min) and 544.0742 (12.2 min) (calcd $[M+2H/2]^+$ = 543.7964) (HPLC analysis at 220 nm). Retention time in HPLC: 12.1 and 12.2 min (28%). Two peaks in LC trace indicate the presence of two isomers.

Ac-KWGALGGFR-CONH$_2$ ($C_{52}H_{73}N_{15}O_{10}$) cyclized peptide products 2h. LCMS: *m/z* 1068.8608 (14.5 min) and 1068.6511 (14.7 min) (calcd $[M+H]^+$ = 1068.5738), (HPLC analysis at 220 nm). Retention time in HPLC: 14.5 and 14.7 min. (48%). Two peaks in LC trace indicate the presence of two isomers.

HPLC Trace of Ac-KWGALGGFR-CONH$_2$ Starting Peptide at 220 nm

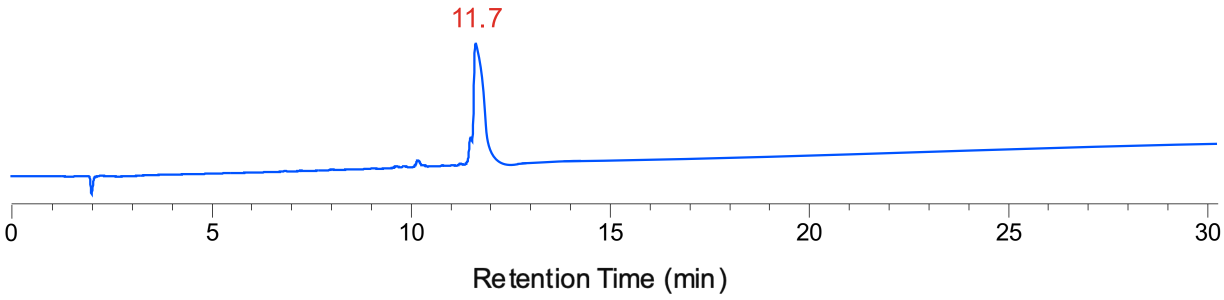

HRMS Trace of Peak at 11.7 min

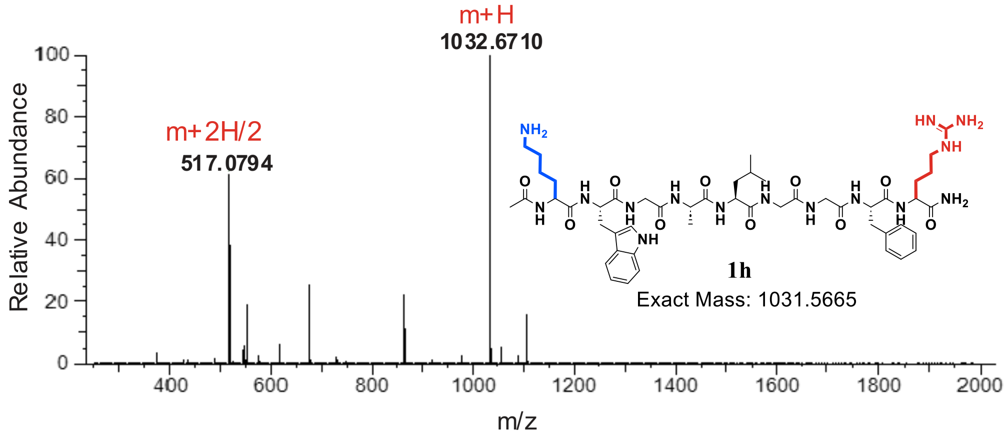

HPLC Trace of Ac-KWGALGGFR-CONH₂ Reaction Mixture at 220 nm

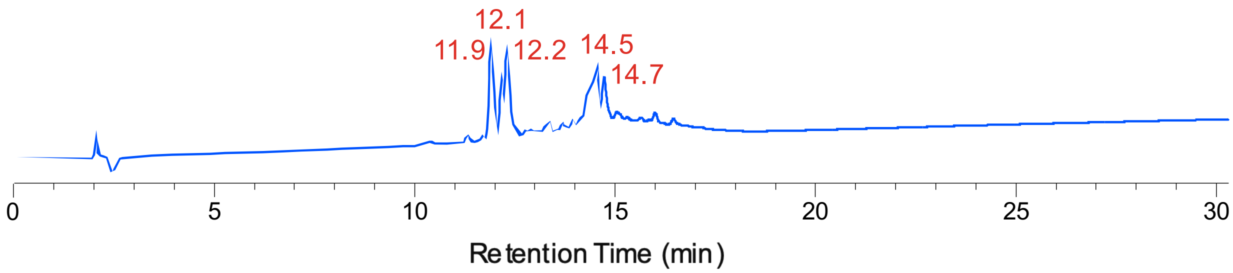

HRMS Trace of Peak at 11.9 min

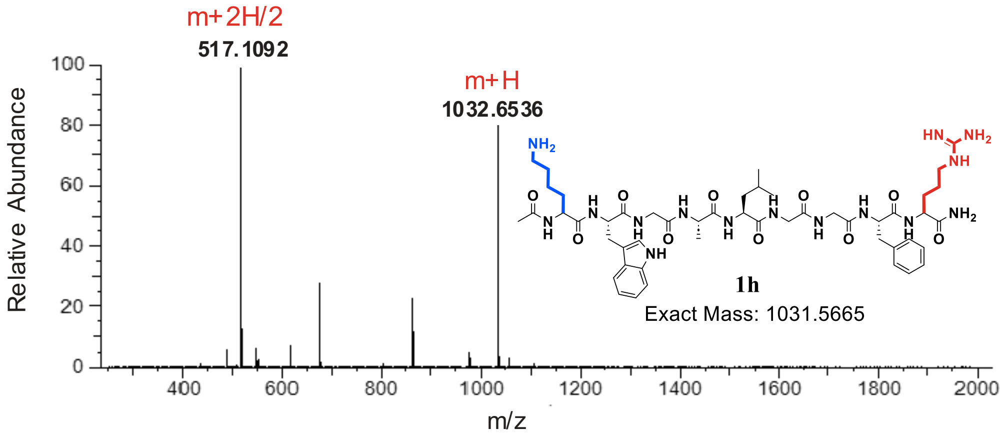

HRMS Trace of Peak at 12.1 min

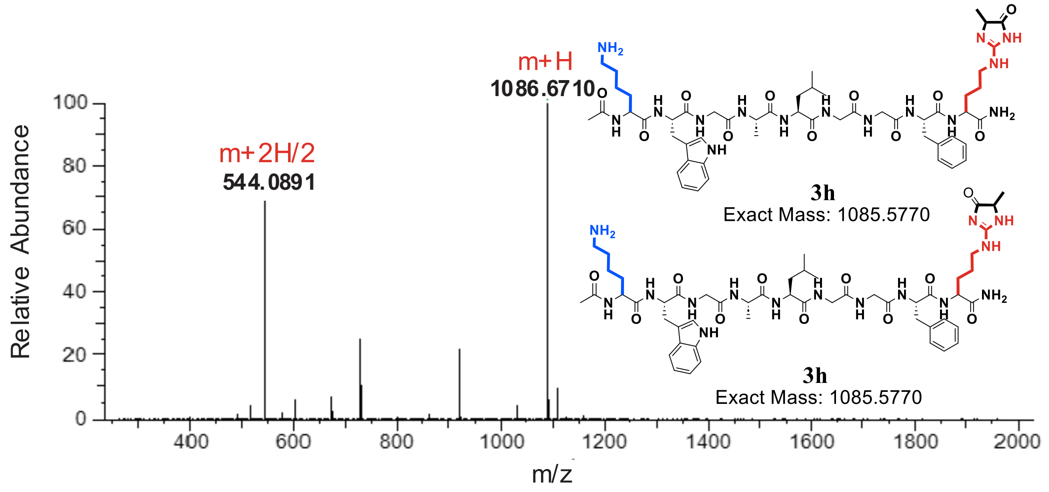

HRMS Trace of Peak at 12.2 min

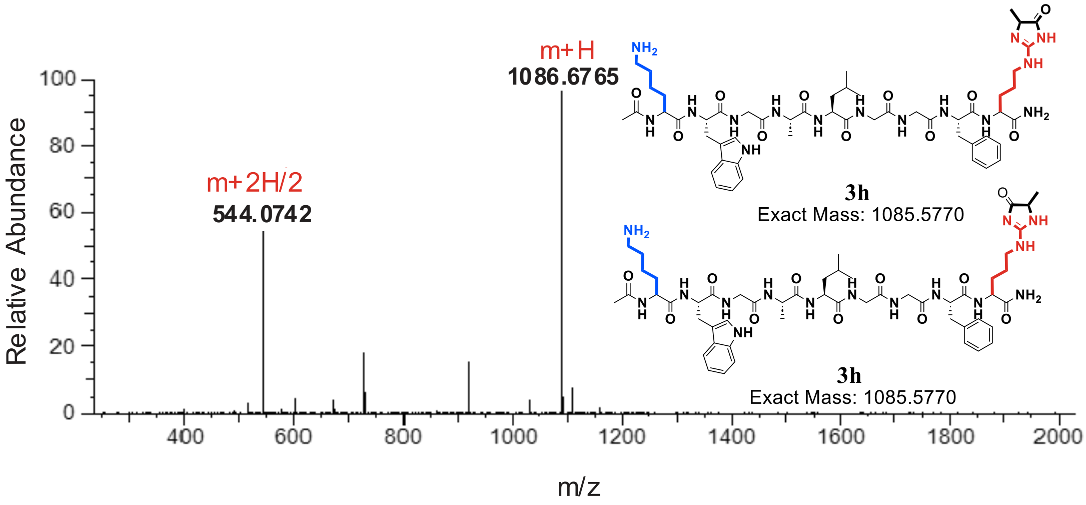

HRMS Trace of Peak at 14.5 and 14.7 min

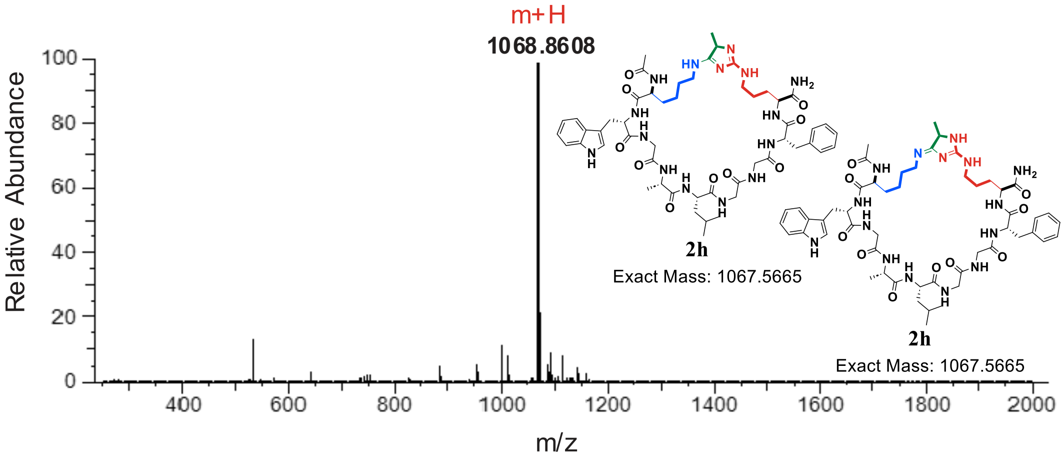

Supplementary Figure 5i. Cyclization of Peptide 1i

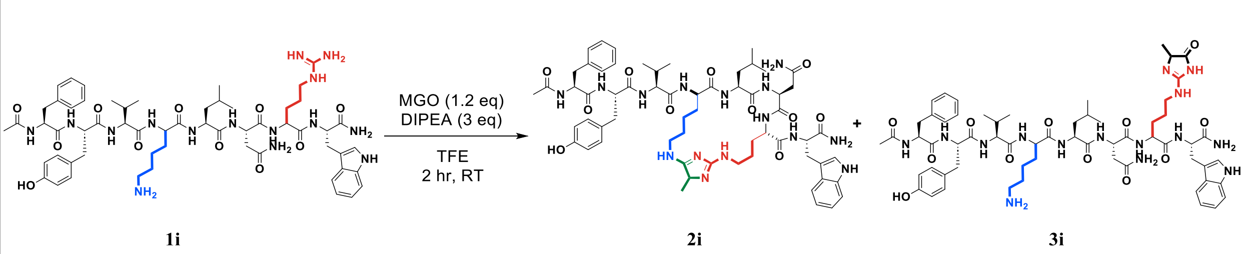

Linear peptide **1i** (1.0 mg, 0.001 mmol, 1.0 eq) was dissolved in 250 µL of TFE and the solution was left to stir at room temperature for 5 minutes with DIPEA (3 eq). Methylglyoxal (1.2 eq) was added to the reaction mixture and was left to stir at room temperature for 8 hours. Samples were taken from the reaction mixture and injected into the HPLC using **HPLC Method A** to determine % conversion. The masses of the products were confirmed with LC-MS and compiled below. The conversion of the cyclized product was determined to be (73%).

Ac-FYVKLNRW-CONH$_2$ ($C_{58}H_{83}N_{15}O_{11}$) linear peptide 1i. LCMS: *m/z* 1166.6471 (calcd $[M+H]^+$ = 1166.6469), *m/z* 583.8273 (calcd $[M+2/2]^+$ = 583.8277) (HPLC analysis at 220 nm). Retention time in HPLC: 13.2 min

Ac-FYVKLNRW-CONH$_2$ ($C_{61}H_{85}N_{15}O_{12}$) arginine adduct products 3i. LCMS: 1220.8184 (13.7 min) and 1220.7490 (13.8 min) (calcd $[M+H]^+$ = 1220.6575), *m/z* 611.1871 (13.7 min) and 611.1370 (13.8 min) (calcd $[M+2H/2]^+$ = 610.8330), *m/z* 1242.7522 (13.7 min) and 1242.7317 (13.8 min) (calcd $[M+Na]^+$ = 1242.6394) (HPLC analysis at 220 nm). Retention time in HPLC: 13.7 and 13.8 min. (27%). Two peaks in LC trace indicate the presence of two isomers.

Ac-FYVKLNRW-CONH$_2$ ($C_{61}H_{83}N_{15}O_{11}$) cyclized peptide products 2i. LCMS: *m/z* 1202.8324 (17.4 min) and 1202.9124 (17.6 min) (calcd $[M+H]^+$ = 1202.6469), (HPLC analysis at 220 nm). Retention time in HPLC: 17.4 min and 17.6 min. (73%). Two peaks in LC trace indicate the presence of two isomers.

HPLC of Ac-FYVKLNRW-CONH$_2$ Starting Peptide at 220 nm

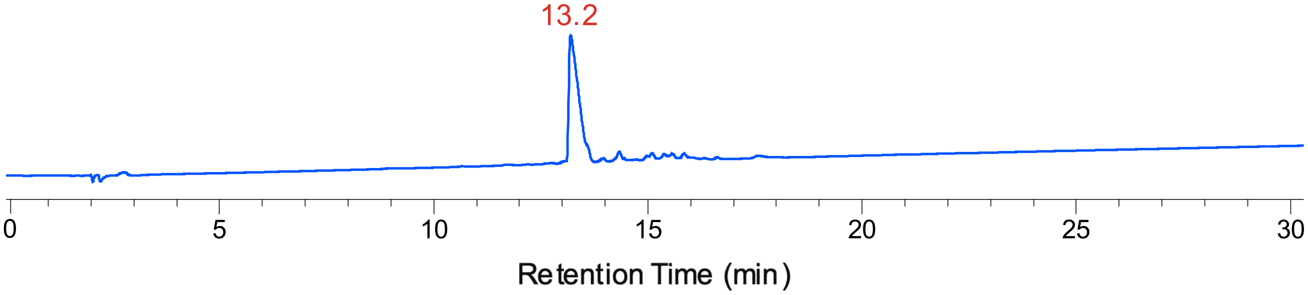

HRMS Trace of Peak at 13.2 min

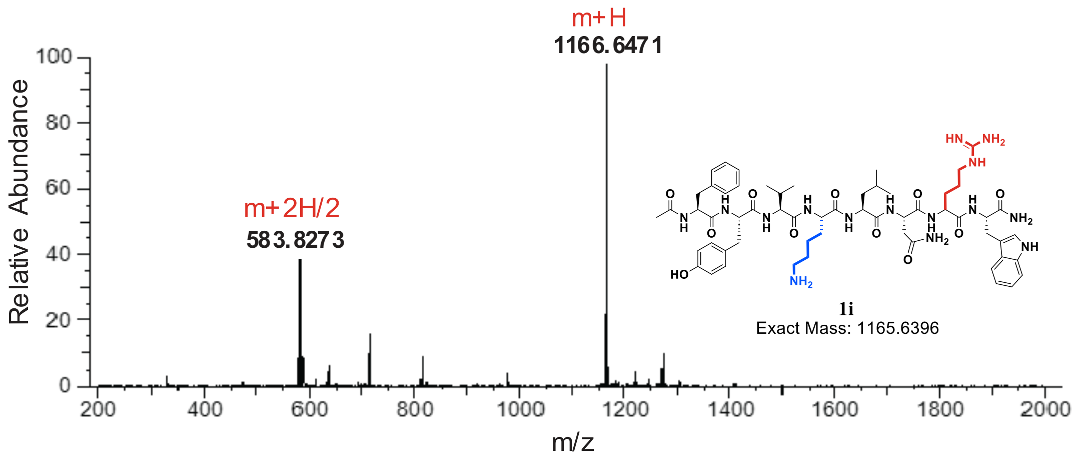

HPLC Trace of Ac-FYVKLNRW-CONH₂ Reaction Mixture at 220 nm

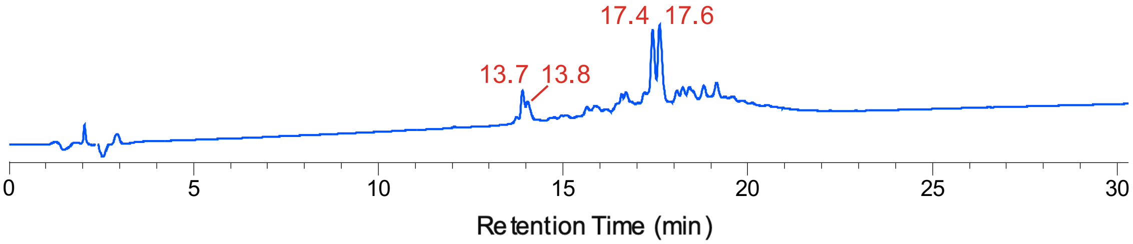

HRMS Trace of Peak at 13.7 min

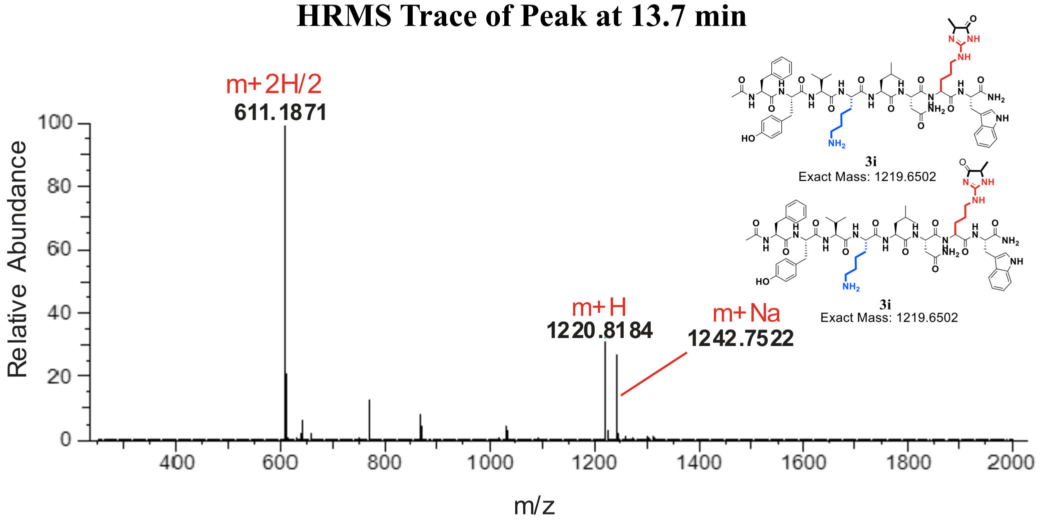

HRMS Trace of Peak at 13.8 min

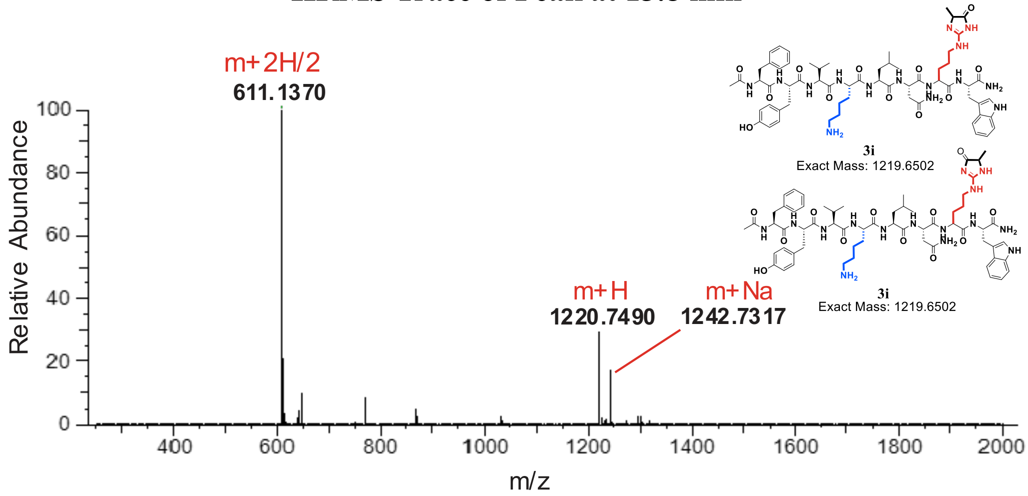

HRMS Trace of Peak at 17.4 and 17.6 min

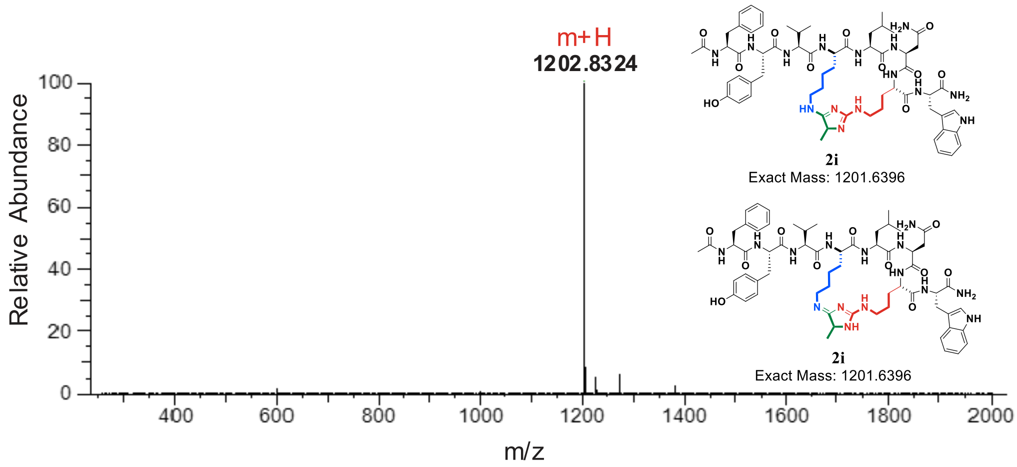

Supplementary Figure 5j.Cyclization of Peptide 1j

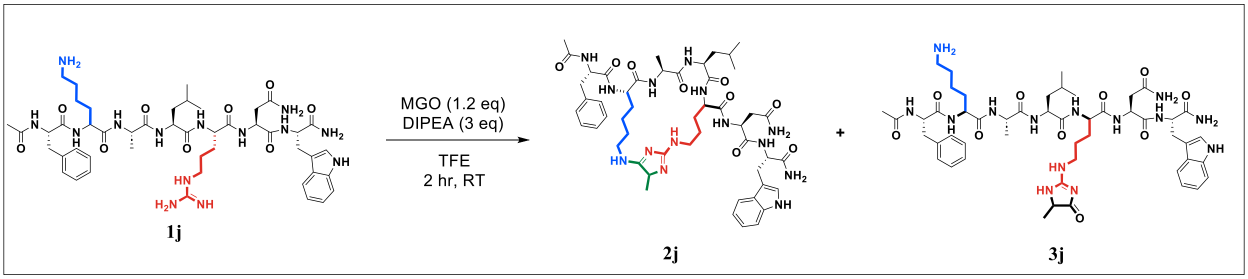

Linear peptide **1j** (1.0 mg, 0.001 mmol, 1.0 eq) was dissolved in 250 µL of TFE and the solution was left to stir at room temperature for 5 minutes with DIPEA (3 eq). Methylglyoxal (1.2 eq) was added to the reaction mixture and was left to stir at room temperature for 8 hours. Samples were taken from the reaction mixture and injected into the HPLC using **HPLC Method A** to determine % conversion. The masses of the products were confirmed with LC-MS and compiled below. The conversion of the cyclized product was determined to be (65%).

Ac-FKALRNW-CONH2 ($C_{47}H_{70}N_{14}O_9$) linear peptide 1j. LCMS: *m/z* 975.5538 (calcd $[M+H]^+$ = 975.5523), (HPLC analysis at 220 nm). Retention time in HPLC: 12.3 min

Ac-FKALRNW-CONH2 ($C_{50}H_{72}N_{14}O_{10}$) arginine adduct products 3j. LCMS: 1029.6107 (12.7 min) and 1029.7301 (13.0 min) (calcd $[M+H]^+$ = 1029.5629), *m/z* 515.7960 (13.0 min) (calcd $[M+2H/2]^+$ = 515.2856) (HPLC analysis at 220 nm). Retention time in HPLC: 12.7 and 13.0 min. (35%). Two peaks in LC trace indicate the presence of two isomers.

Ac-FKALRNW-CONH2 ($C_{50}H_{70}N_{14}O_9$) cyclized peptide products 2j. LCMS: *m/z* 1011.6789 (16.5 min) and 1011.7299 (16.6 min) (calcd $[M+H]^+$ = 1011.5523), (HPLC analysis at 220 nm). Retention time in HPLC: 16.5 and 16.6 min. (65%). Two peaks in LC trace indicate the presence of two isomers.

HPLC Trace of Ac-FKALRNW-CONH2 Starting Peptide at 220 nm

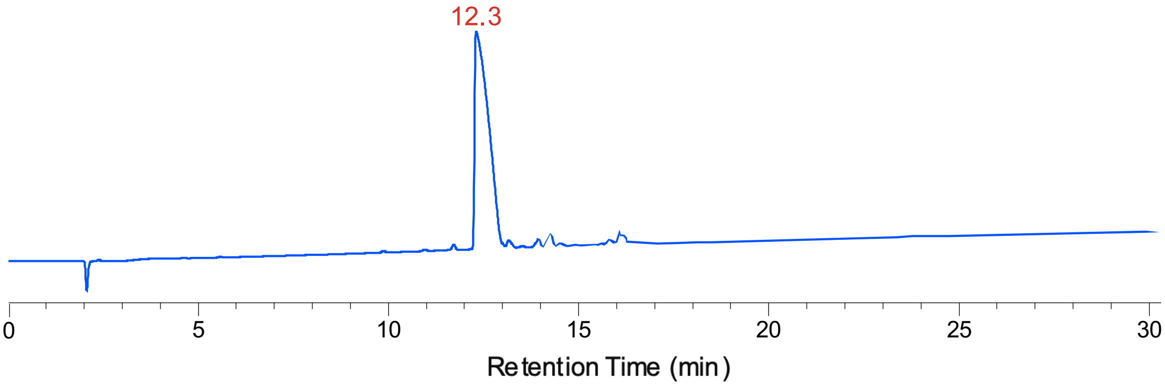

HRMS Trace of Peak at 12.3 min

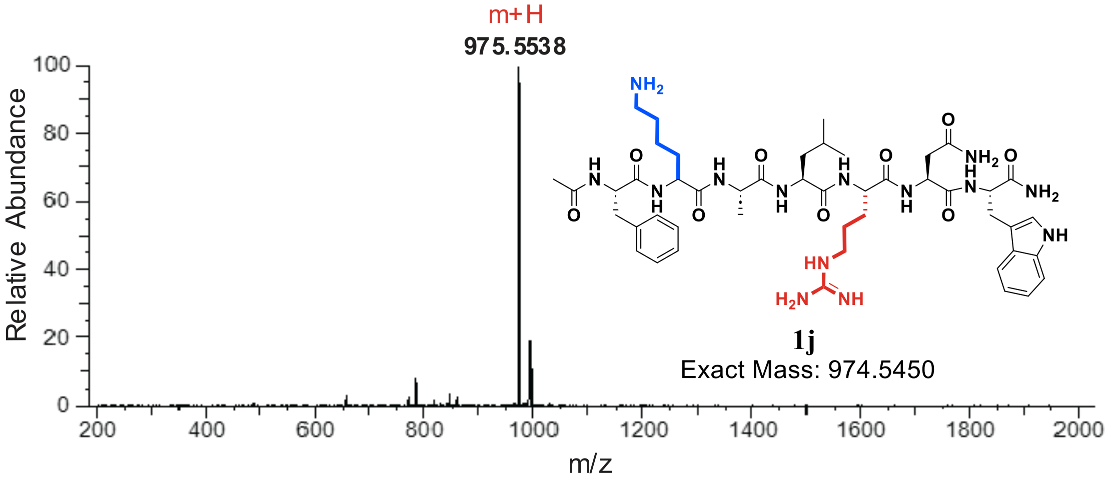

HPLC Trace of Ac-FKALNRW-CONH$_2$ Reaction Mixture at 220 nm

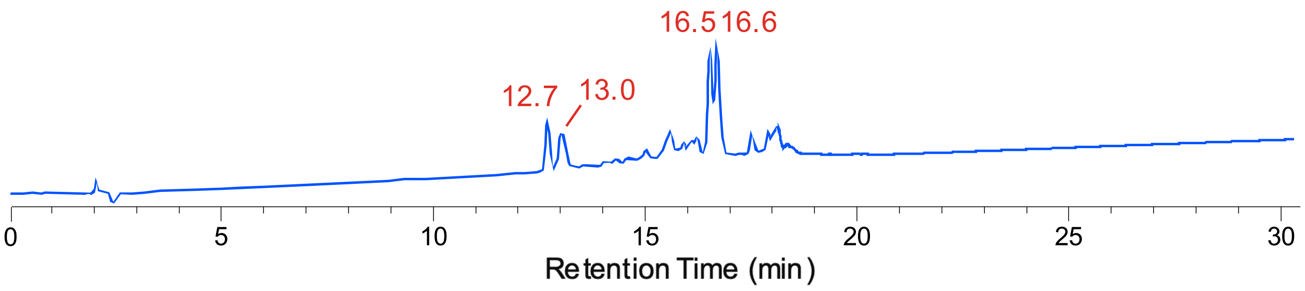

HRMS Trace of Peak at 12.7 min

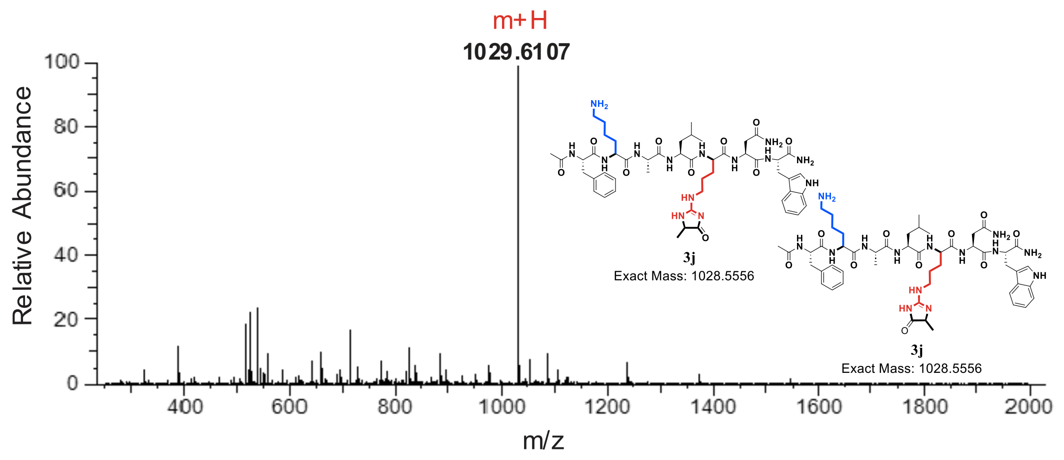

HRMS Trace of Peak at 13.0 min

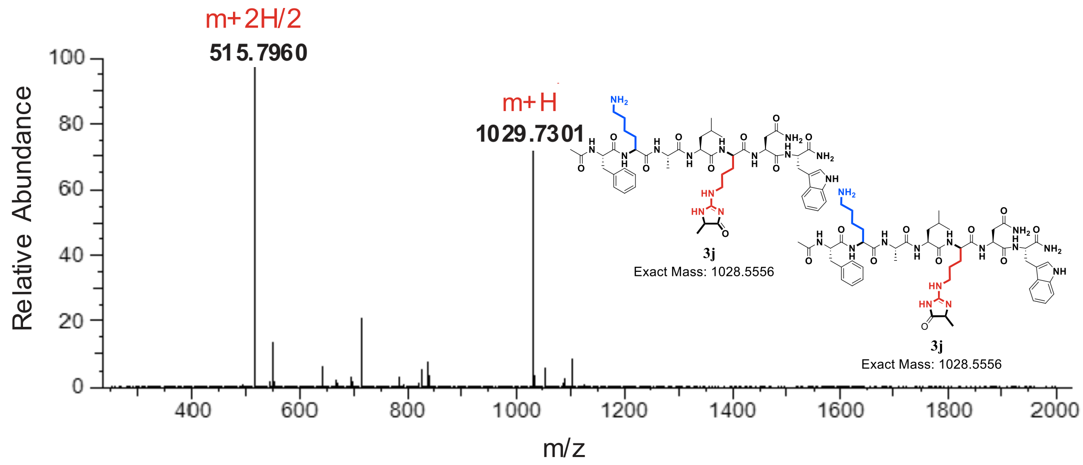

HRMS Trace of Peak at 16.5 and 16.6 min

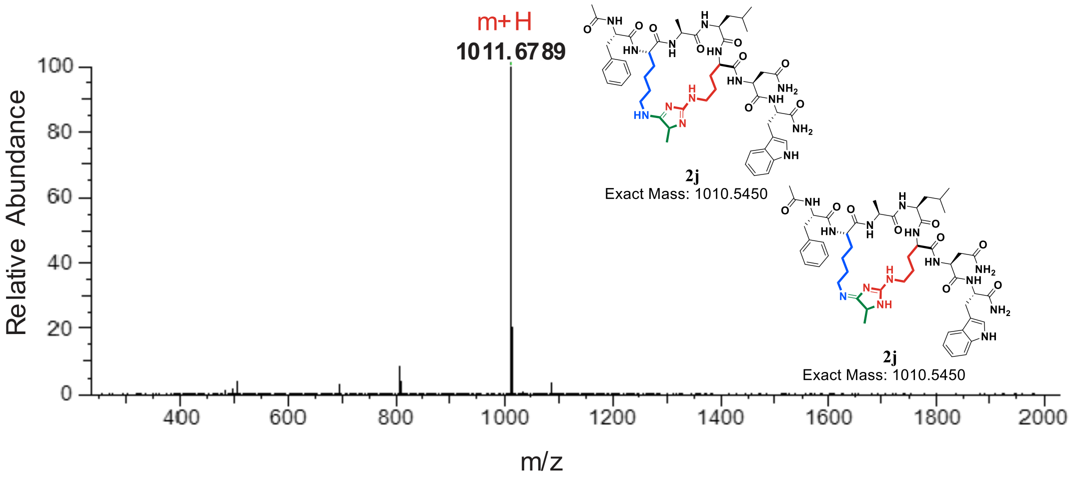

Supplementary Figure 5k. Cyclization of Peptide 1k

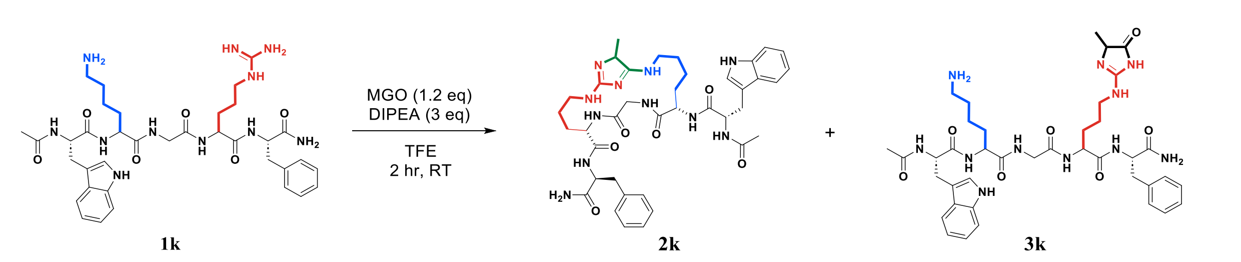

Linear peptide **1k** (1.0 mg, 0.001 mmol, 1.0 eq) was dissolved in 250 µL of TFE and the solution was left to stir at room temperature for 5 minutes with DIPEA (3 eq). Methylglyoxal (1.2 eq) was added to the reaction mixture and was left to stir at room temperature for 2-3 hours. Samples were taken from the reaction mixture and injected into the HPLC using **HPLC Method A** to determine % conversion. The masses of the products were confirmed with LC-MS and compiled below. The conversion of the cyclized product was determined to be (45%).

Ac-WKGRF-CONH₂ ($C_{36}H_{51}N_{11}O_6$) linear peptide 1k. LCMS: *m/z* 734.4100 (calcd $[M+H]^+$ = 734.4097), *m/z* 367.7086 (calcd $[M+2H/2]^+$ = 367.7090), (HPLC analysis at 220 nm). Retention time in HPLC: 10.0 min

Reaction Mixture:

Ac-WKGRF-$CONH_2$ ($C_{36}H_{51}N_{11}O_6$) linear peptide 1k. LCMS: *m/z* 734.4099 (calcd $[M+H]^+$ = 734.4097), (HPLC analysis at 220 nm). Retention time in HPLC: 10.1 min. (9%)

Ac-WKGRF-$CONH_2$ ($C_{39}H_{53}N_{11}O_7$) arginine adduct products 3k. LCMS: 788.4188 *m/z* (10.3 min) and 788.4202 (10.6 min) (calcd $[M+H]^+$ = 788.4202), *m/z* 394.7130 (10.3 min) (calcd $[M+2H/2]^+$ = 394.7143) (HPLC analysis at 220 nm). Retention time in HPLC: 10.3 and 10.6 min. (46%). Two peaks in LC trace indicate the presence of two isomers.

Ac-WKGRF-$CONH_2$ ($C_{39}H_{51}N_{11}O_6$) cyclized peptide products 2k. LCMS: *m/z* 770.4099 (12.5 min) and 770.4095 (12.7 min) (calcd $[M+H]^+$ = 770.4097), (HPLC analysis at 220 nm). Retention

time in HPLC: 12.5 and 12.7 min. (45%). Two peaks in LC trace indicate the presence of two isomers.

HPLC Trace of Ac-WKGRF-CONH₂ Starting Peptide at 220 nm

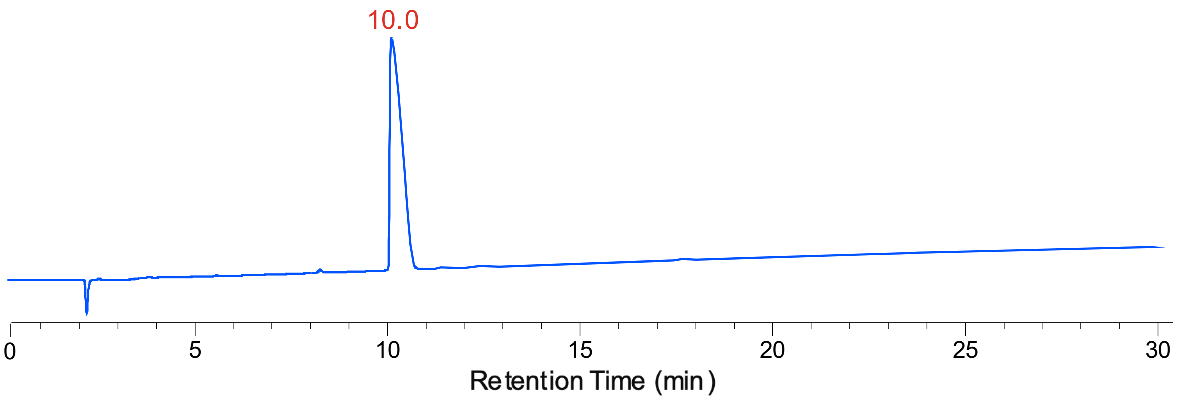

HRMS Trace of Peak at 10.0 min

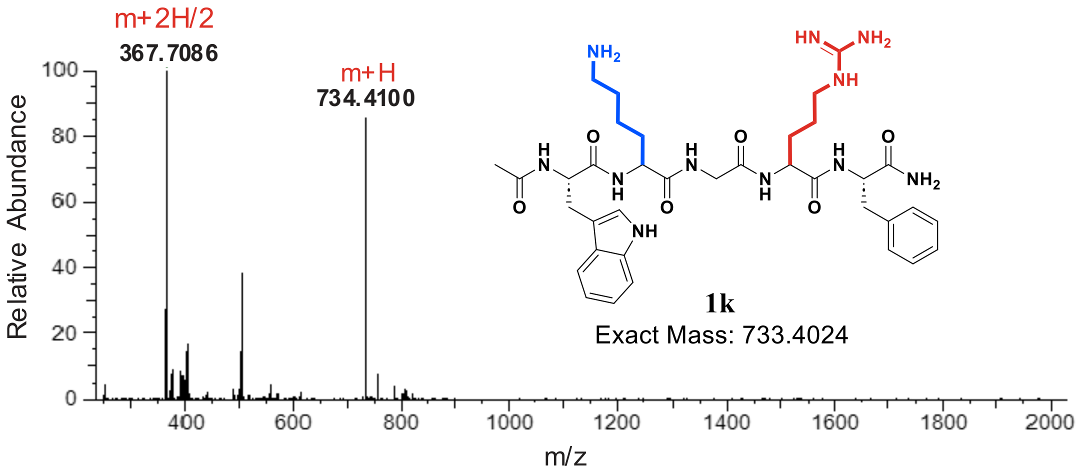

HPLC Trace of Ac-WKGRF-CONH₂ Reaction Mixture at 220nm

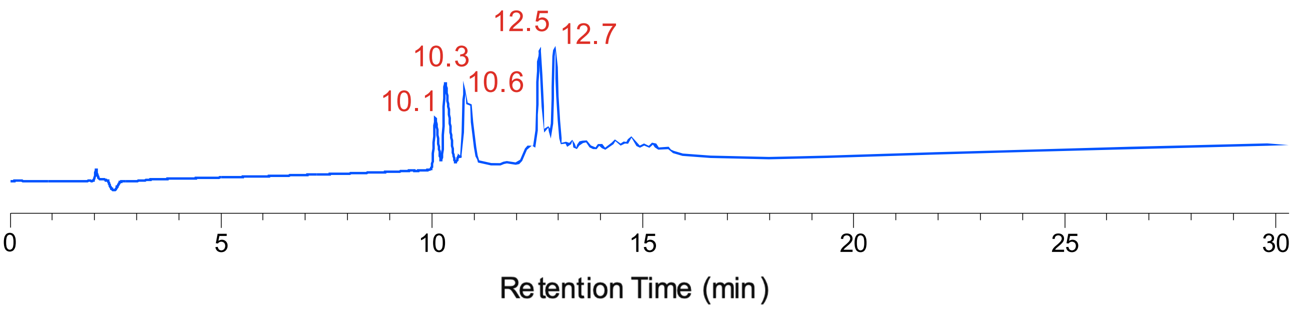

HRMS Trace of Peak at 10.1 min

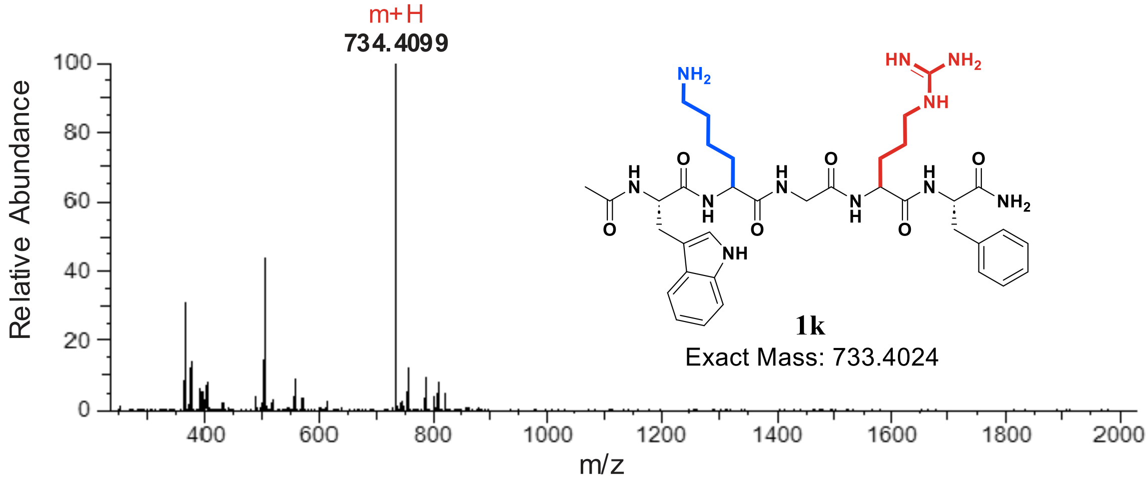

HRMS Trace of Peak at 10.3 min

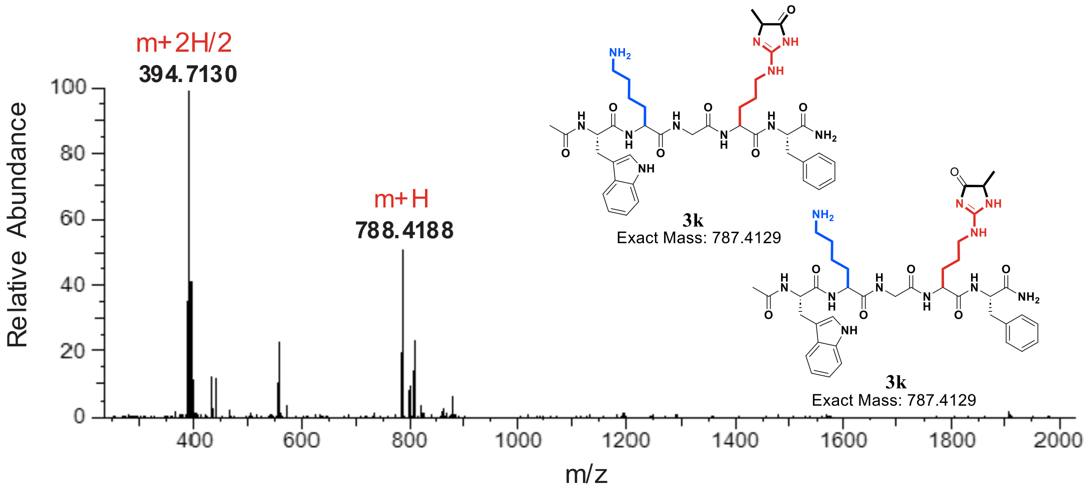

HRMS Trace of Peak at 10.6 min

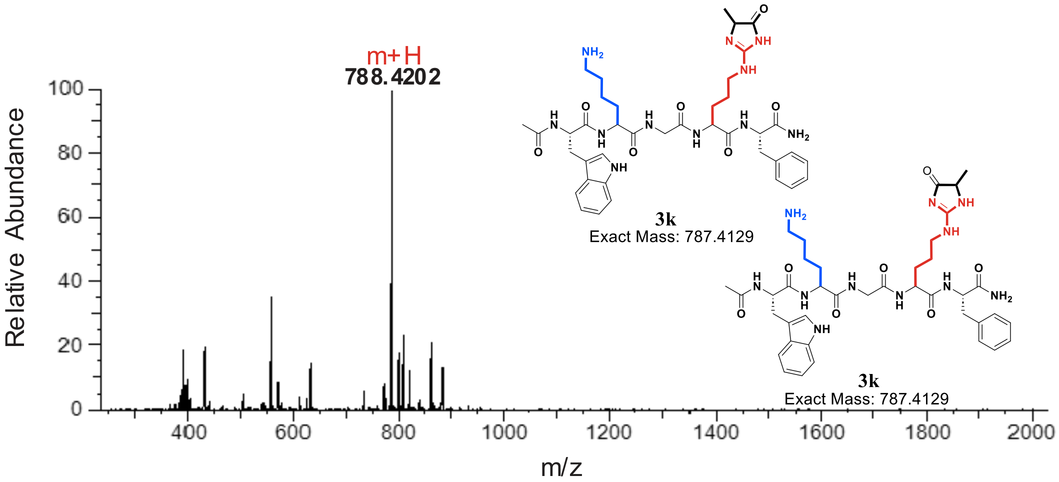

HRMS Trace of Peak at 12.5 and 12.7 min

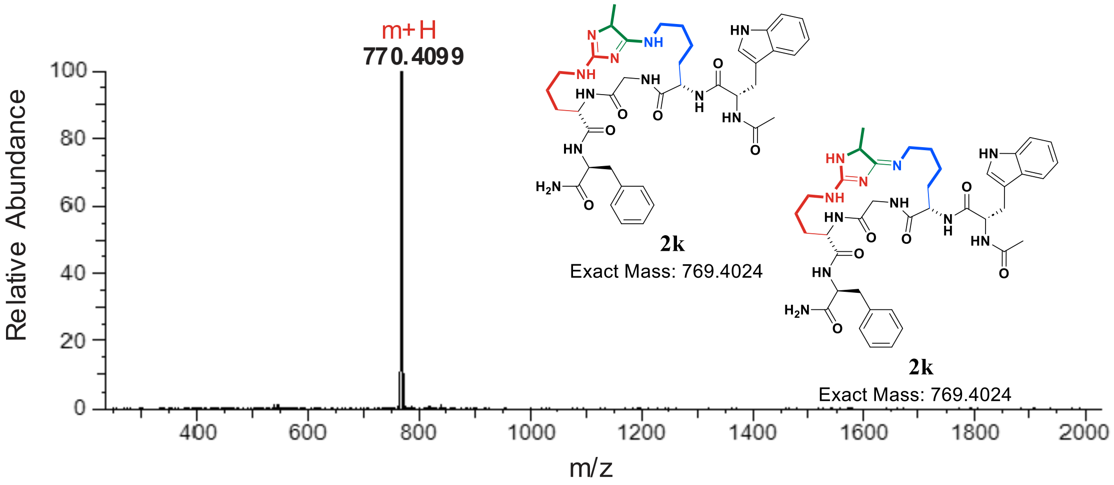

Supplementary Figure 5l. Cyclization of Peptide 1l

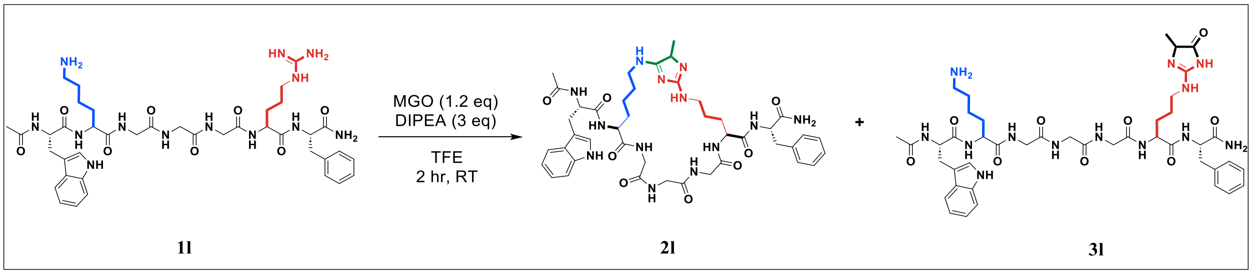

Linear peptide **1l** (1.0 mg, 0.001 mmol, 1.0 eq) was dissolved in 250 μL of TFE and the solution was left to stir at room temperature for 5 minutes with DIPEA (3 eq). Methylglyoxal (1.2 eq) was added to the reaction mixture and was left to stir at room temperature for 3-4 hours. Samples were taken from the reaction mixture and injected into the HPLC using **HPLC Method A** to determine % conversion. The masses of the products were confirmed with LC-MS and compiled below. The conversion of the cyclized product was determined to be (52%).

Ac-WKGGGRF-CONH$_2$ ($C_{40}H_{57}N_{13}O_8$) linear peptide 1l. LCMS: *m/z* 848.4827 (calcd $[M+H]^+$ = 848.4526), *m/z* 424.9517 (calcd $[M+2H/2]^+$ = 424.7305), (HPLC analysis at 220 nm). Retention time in HPLC: 9.3 min

Reaction Mixture:

Ac-WKGGGRF-CONH$_2$ ($C_{40}H_{57}N_{13}O_8$) linear peptide 1l. LCMS: *m/z* 848.6506 (calcd $[M+H]^+$ = 848.4526), *m/z* 425.3295 (calcd $[M+2H/2]^+$ = 424.7305), (HPLC analysis at 220 nm). Retention time in HPLC: 9.6 min. (21%)

Ac-WKGGGRF-CONH$_2$ ($C_{43}H_{59}N_{13}O_9$) arginine adduct products 3l. LCMS: *m/z* 902.4714 (9.9 min) and 902.4867 (10.3 min) (calcd $[M+H]^+$ = 902.4631), *m/z* 451.9451 (9.9 min) and 451.9502 (10.3 min) (calcd $[M+2H/2]^+$ = 451.7358), (HPLC analysis at 220 nm). Retention time in HPLC: 9.9 and 10.3 min. (27%). Two peaks in LC trace indicate the presence of two isomers.

Ac-WKGGGRF-CONH$_2$ ($C_{43}H_{57}N_{13}O_8$) cyclized peptide products 2l. LCMS: *m/z* 884.5677 (calcd $[M+H]^+$ = 884.4526, (HPLC analysis at 220 nm). Retention time in HPLC: 12.1 min. (52%).

HPLC Trace of Ac-WKGGGRF-CONH₂ Starting Peptide at 220 nm

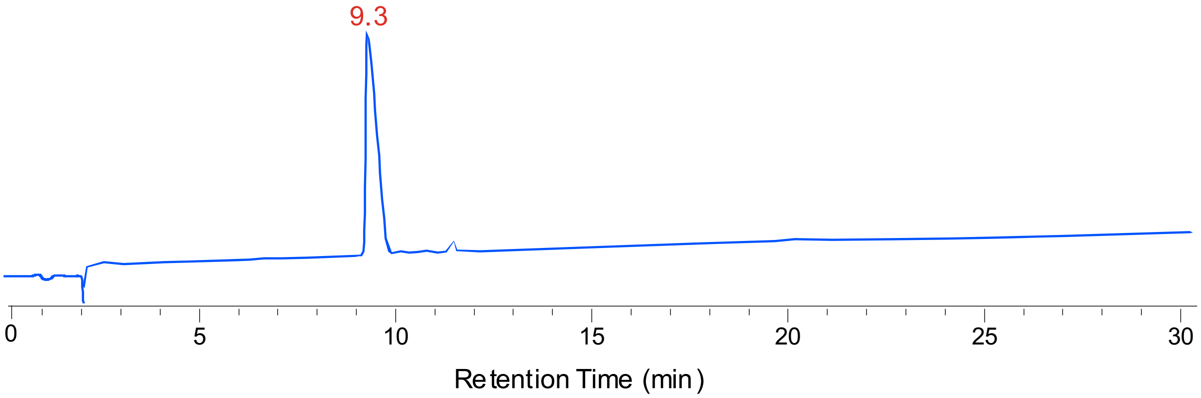

HRMS Trace of Peak at 9.3 min

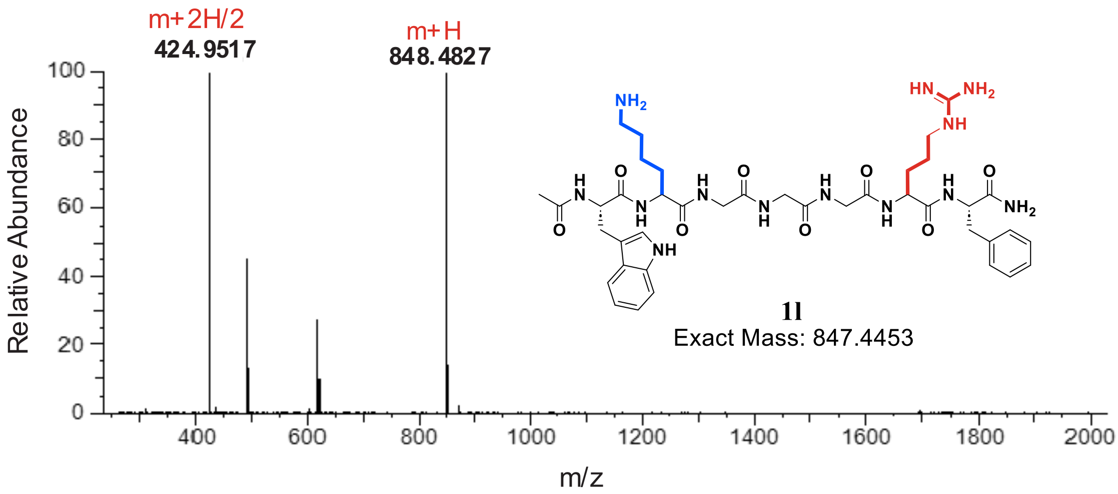

HPLC Trace of Ac-WKGGGRF-CONH2 Reaction Mixture at 220 nm

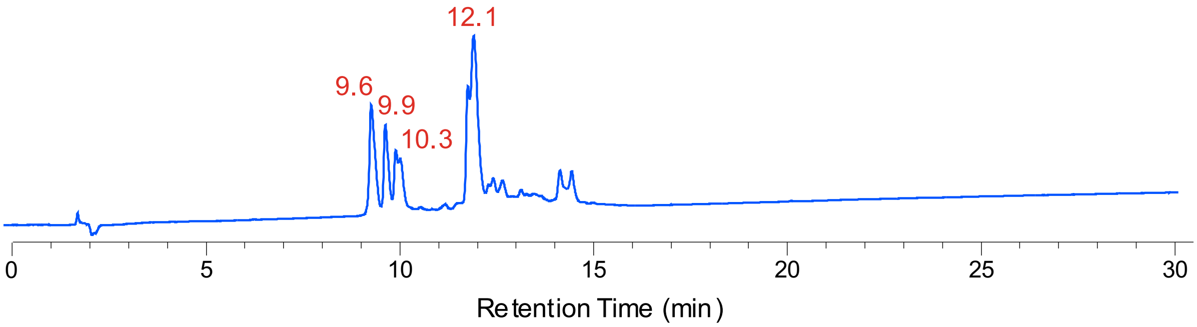

HRMS Trace of Peak at 9.6 min

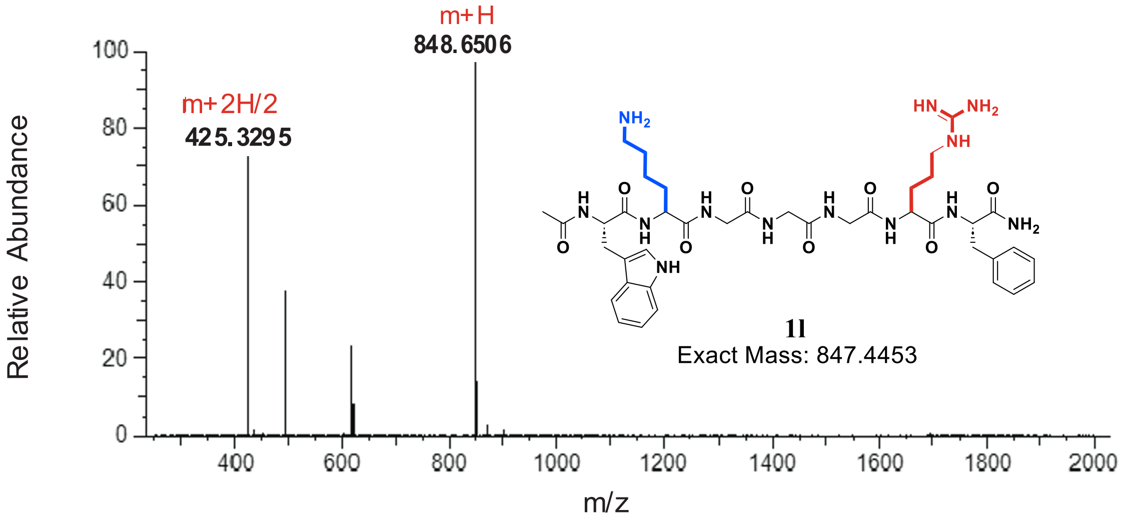

HRMS Trace of Peak at 9.9 min

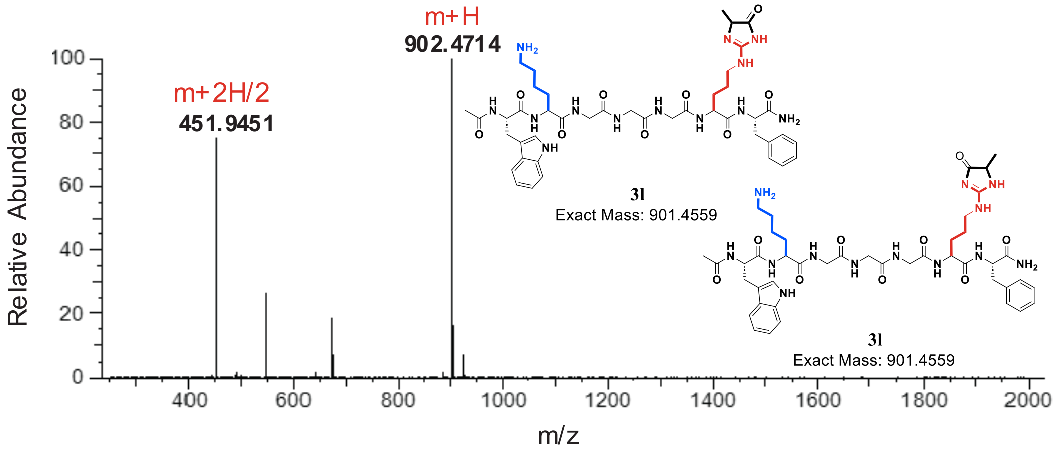

HRMS Trace of Peak at 10.3 min

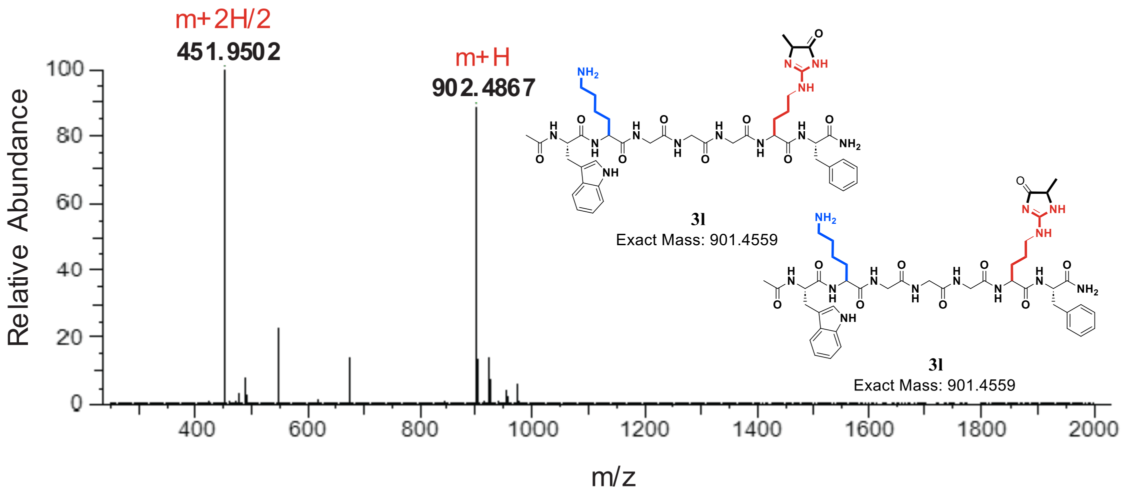

HRMS Trace of Peak at 12.1 min

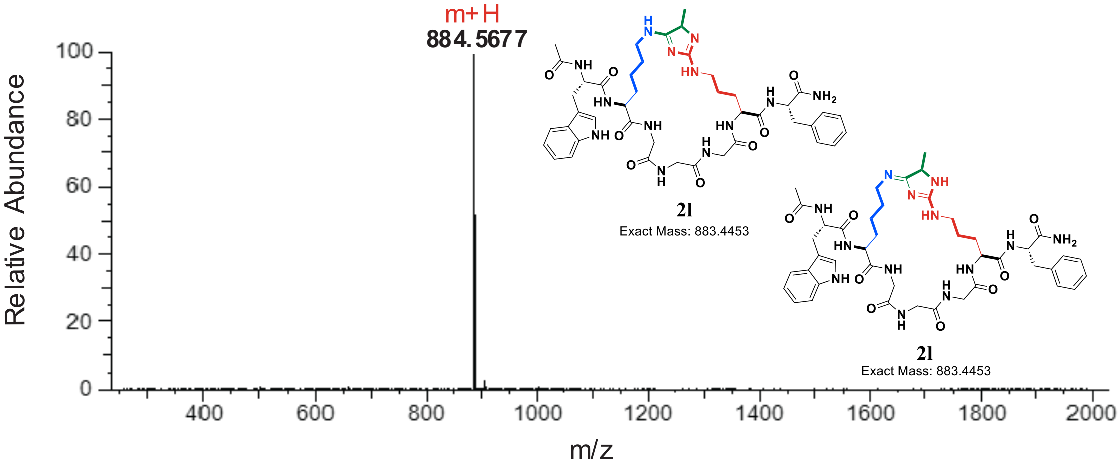

Supplementary Figure 5m. Cyclization of Peptide 1l’

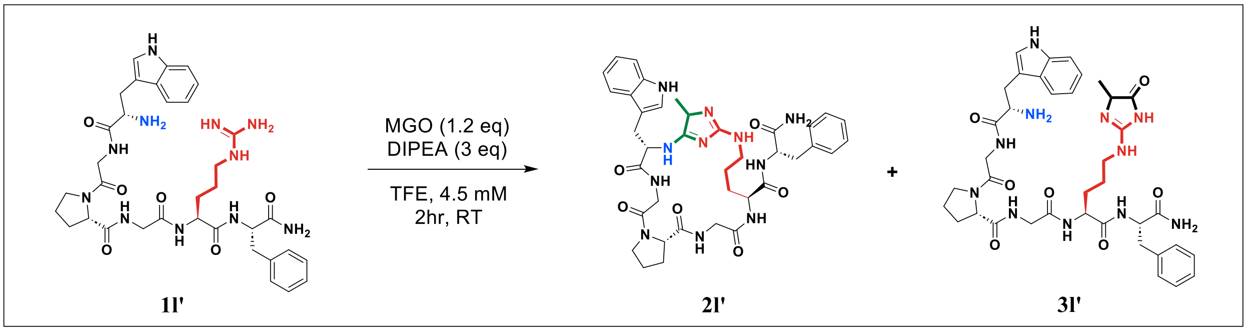

Linear peptide **1l’** (1.0 mg, 0.001 mmol, 1.0 eq) was dissolved in 250 µL of TFE and the solution was left to stir at room temperature for 5 minutes with DIPEA (3 eq). Methylglyoxal (1.2 eq) was added to the reaction mixture and was left to stir at room temperature for 3-4 hours. Samples were taken from the reaction mixture and injected into the HPLC using **HPLC Method A** to determine

% conversion. The masses of the products were confirmed with LC-MS and compiled below. The conversion of the cyclized product was determined to be (59%).

WGPGRF-CONH$_2$ ($C_{35}H_{47}N_{11}O_6$) linear peptide 1l'. LCMS: *m/z* 718.5417 (calcd $[M+H]^+$ = 718.3784) (HPLC analysis at 220 nm). Retention time in HPLC: 7.3 min

WGPGRF-CONH$_2$ ($C_{38}H_{49}N_{11}O_7$) arginine adduct products 3l'. LCMS: 772.5475 (calcd $[M+H]^+$ = 772.3889) (HPLC analysis at 220 nm). Retention time in HPLC: 7.6 min. (41%). Two peaks in LC trace indicate the presence of two isomers.

WGPGRF-CONH$_2$ ($C_{38}H_{47}N_{11}O_6$) cyclized peptide products 2l'. LCMS: *m/z* 754.5650 (11.2 min) and 754.5846 (11.5 min) (calcd $[M+H]^+$ = 754.3784) (HPLC analysis at 220 nm). Retention time in HPLC: 11.2 and 11.5 min. (59%). Two peaks in LC trace indicate the presence of two isomers.

HPLC of WGPGRF-CONH$_2$ Starting Peptide at 220 nm

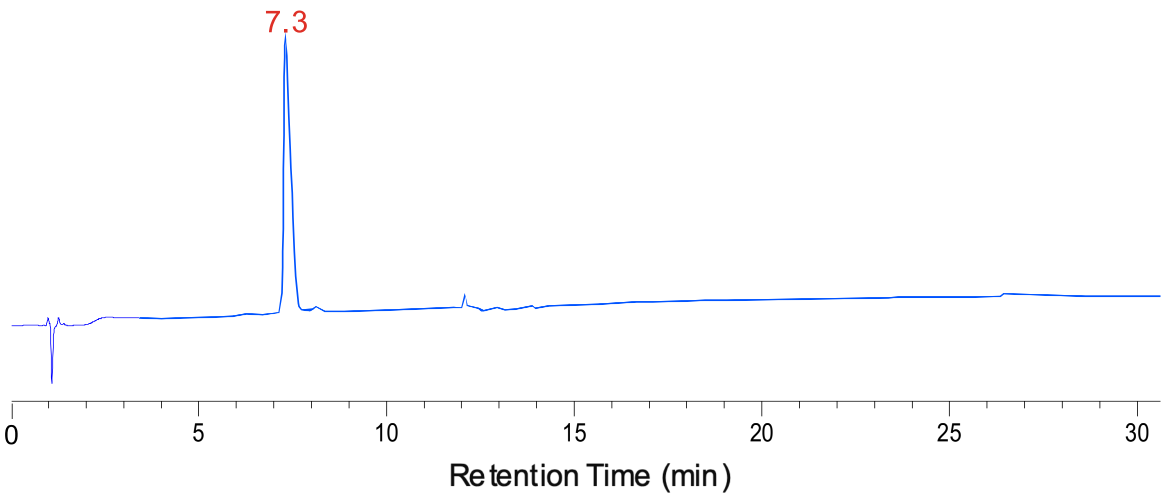

HRMS Trace of Peak at 7.3 min

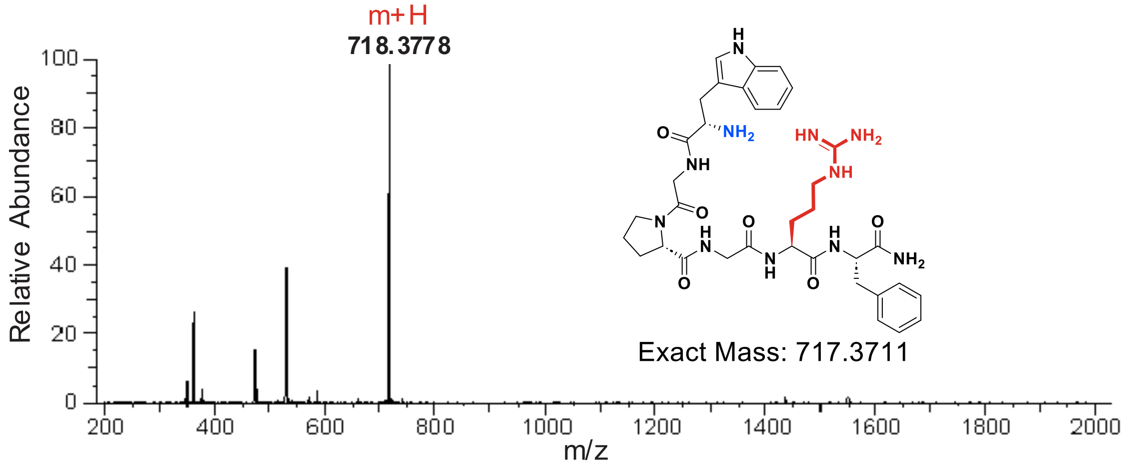

HPLC Trace of WGPGRF-CONH$_2$ Reaction Mixture at 220 nm

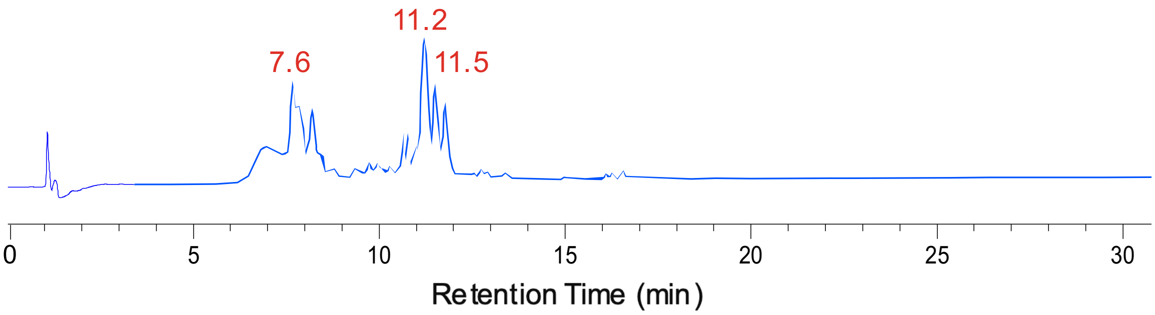

HRMS Trace of Peak at 7.6 min

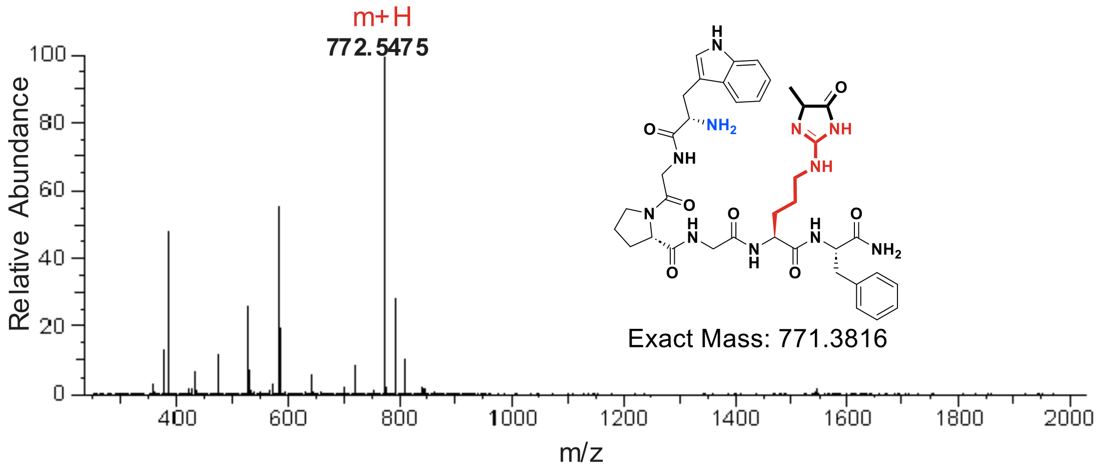

HRMS Trace of Peak at 11.2 and 11.5 min

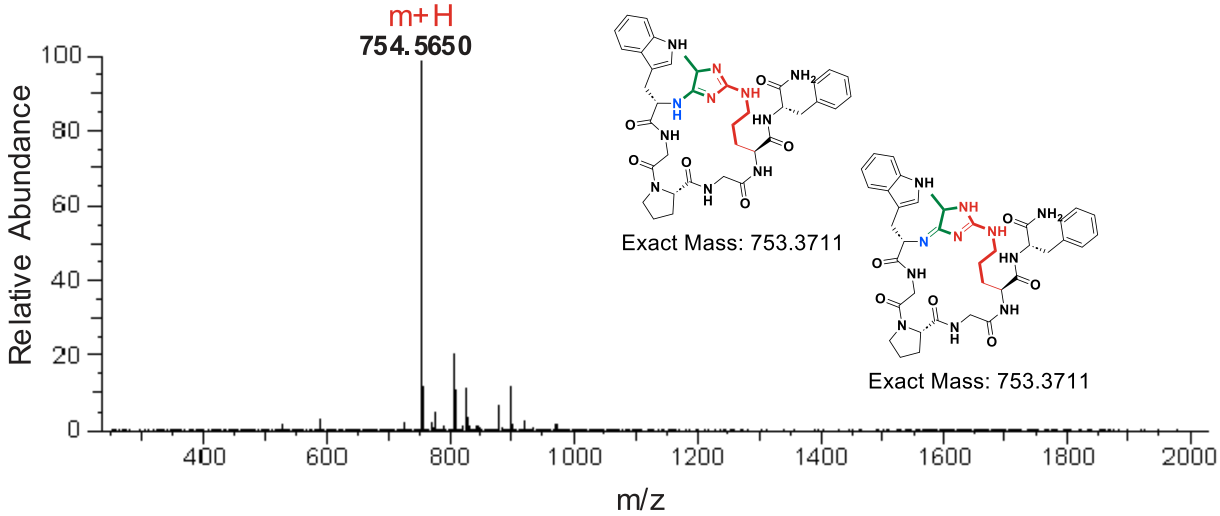

Supplementary Figure 6a: Intermolecular Labeling of Peptide 1m with Guanidine Hydrochloride

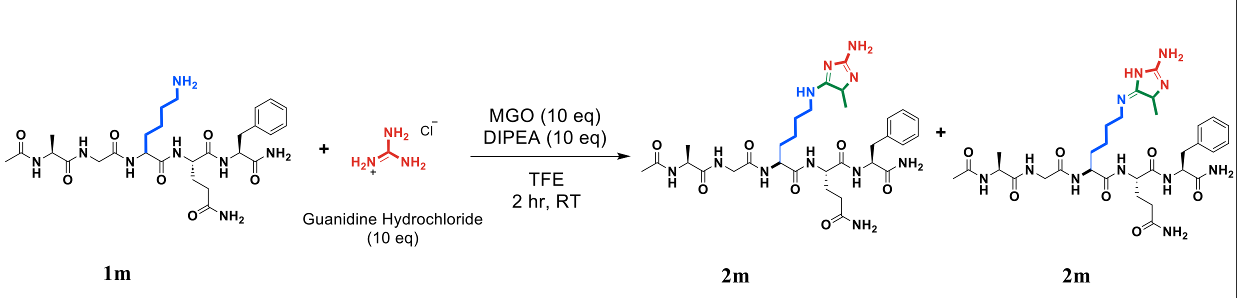

Linear peptide **1m** (1.0 mg, 0.002 mmol, 1.0 eq) was dissolved in 250 µL of TFE and the solution stirred at room temperature for 20 minutes with DIPEA (10 eq) and methylglyoxal (10 eq). Then, guanidine hydrochloride (10 eq) was added, and the reaction mixture was left to stir at room temperature for 2 hours. Samples were taken from the reaction mixture and injected into the HPLC using **HPLC Method A** to determine % conversion. The masses of the products were confirmed with LC-MS and compiled below. The conversion of the labeled product was determined to be (>98%).

Ac-AGKQF-CONH2 ($C_{27}H_{42}N_8O_7$) linear peptide 1m. LCMS: *m/z* 591.3249 (calcd $[M+H]^+$ = 591.3249) (HPLC analysis at 220 nm). Retention time in HPLC: 6.0 min

Ac-AGKQF-CONH2 ($C_{31}H_{47}N_{11}O_7$) labeled products 2m. LCMS: 686.3729 (calcd $[M+H]^+$ = 686.3733) (HPLC analysis at 220 nm). Retention time in HPLC: 8.3 min. (>98%)

HPLC of Ac-AGKQF-CONH2 Starting Peptide at 220 nm

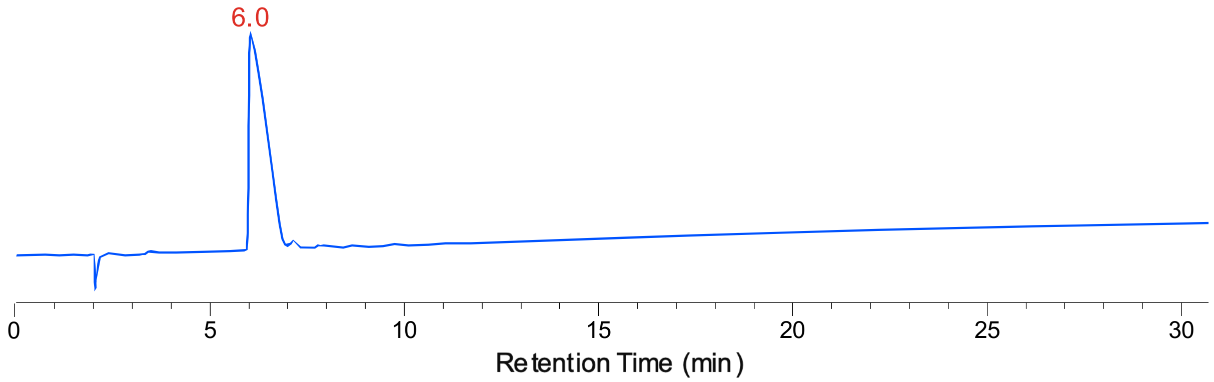

HRMS Trace of Peak at 6.0 min

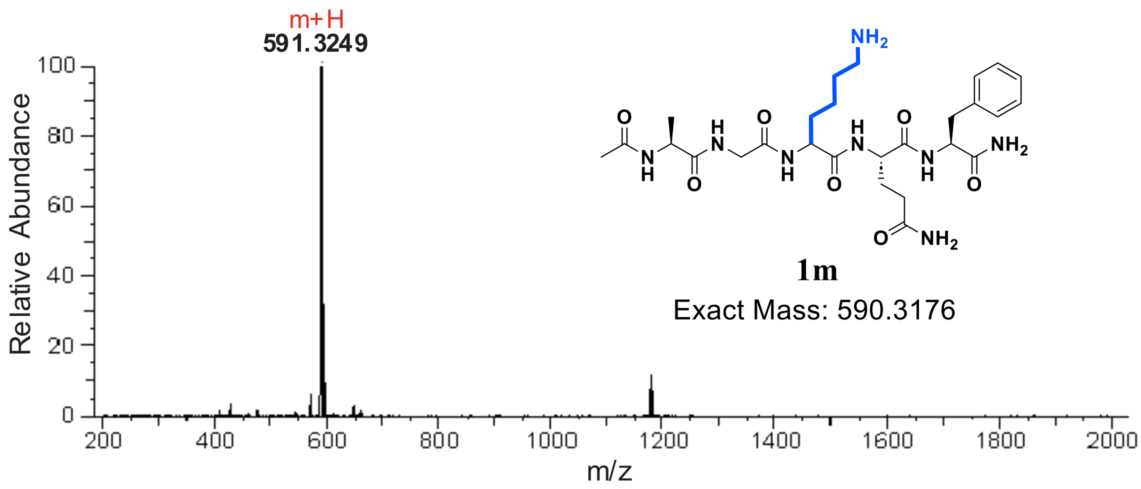

HPLC Trace of Ac-AGKQF-CONH₂ Reaction Mixture at 220 nm

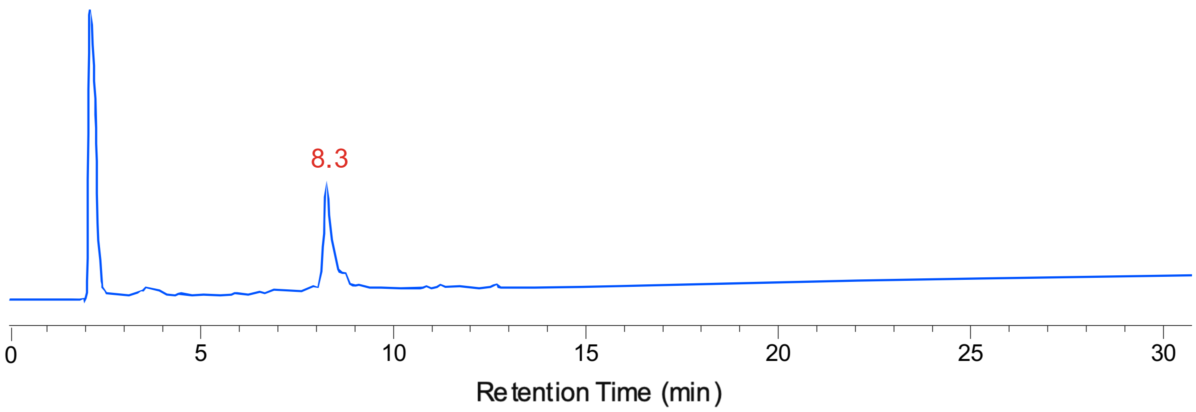

HRMS Trace of Peak at 8.3 min

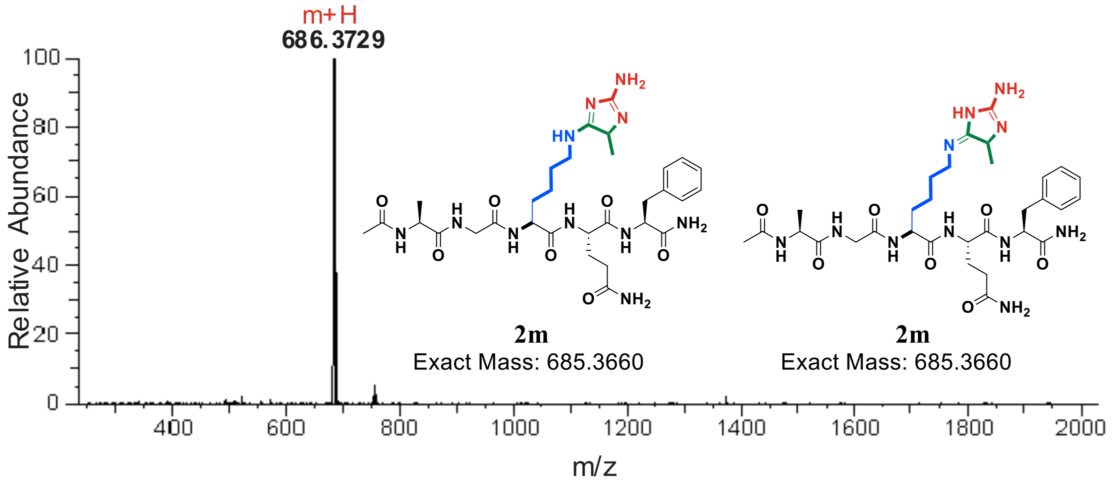

Supplementary Figure 6b: Intermolecular Labeling of Peptide 1n with Guanidine Hydrochloride

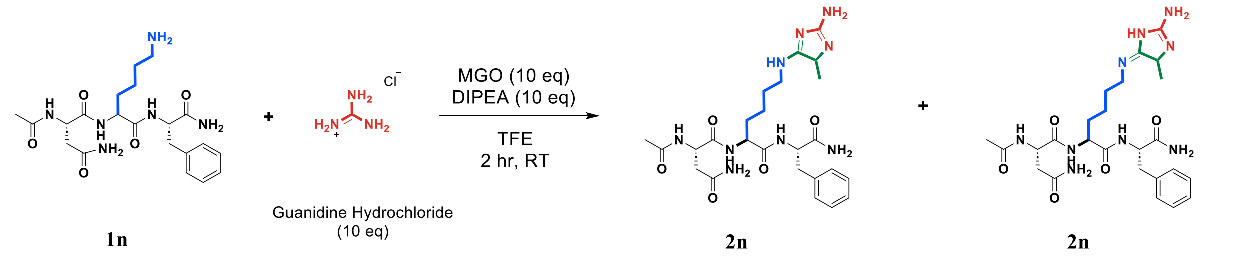

Linear peptide **1n** (1.0 mg, 0.002 mmol, 1.0 eq) was dissolved in 250 µL of TFE and the solution stirred at room temperature for 20 minutes with DIPEA (10 eq) and methylglyoxal (10 eq). Then, guanidine hydrochloride (10 eq) was added and the reaction mixture was left to stir at room temperature for 2 hours. Samples were taken from the reaction mixture and injected into the HPLC using **HPLC Method A** to determine % conversion. The masses of the products were confirmed with LC-MS and compiled below. The conversion of the labeled product was determined to be (>98%).

Ac-NKF-CONH$_2$ ($C_{21}H_{32}N_6O_5$) linear peptide 1n. LCMS: *m/z* 449.2506 (calcd $[M+H]^+$ = 449.2507), *m/z* 471.2325 (calcd $[M+2H/2]^+$ = 471.2326) (HPLC analysis at 220 nm). Retention time in HPLC: 5.4 min

Ac-NKF-CONH$_2$ ($C_{25}H_{37}N_9O_5$) labeled products 2n. LCMS: 544.3426 (calcd $[M+H]^+$ = 544.2990) (HPLC analysis at 220 nm). Retention time in HPLC: 8.1 min. (>98%)

HPLC of Ac-NKF-CONH$_2$ Starting Peptide at 220 nm

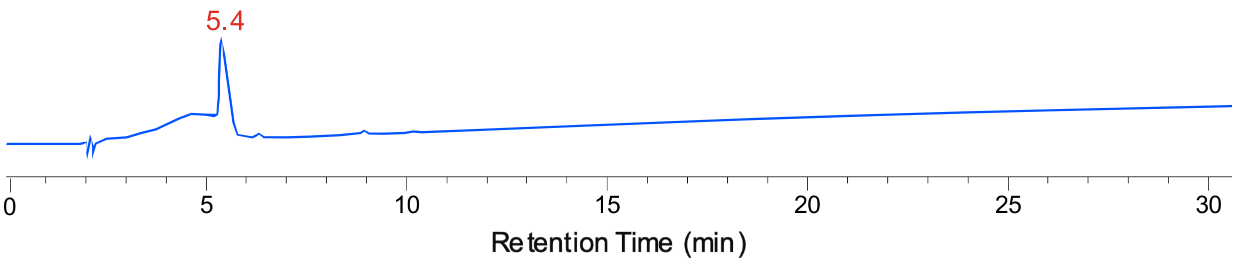

HRMS Trace of Peak at 5.4 min

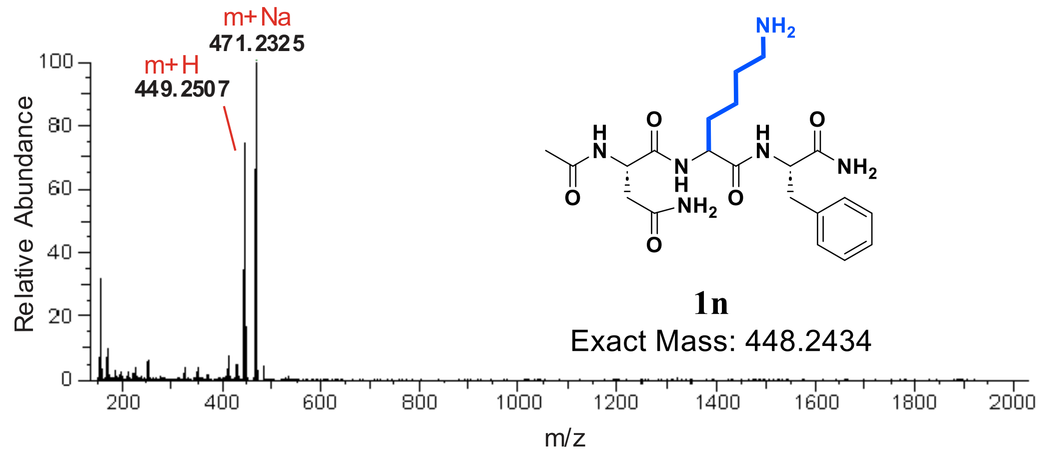

HPLC Trace of Ac-NKF-CONH₂ Reaction Mixture at 220 nm

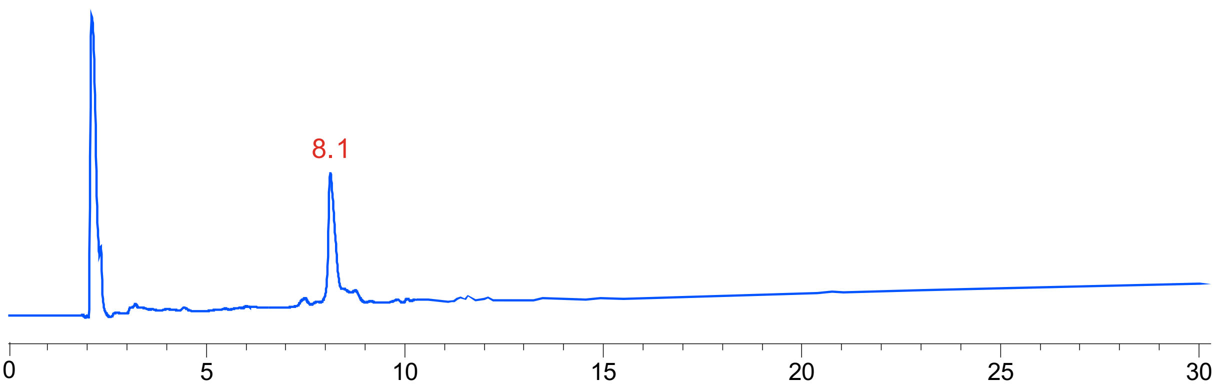

HRMS Trace of Peak at 8.1 min

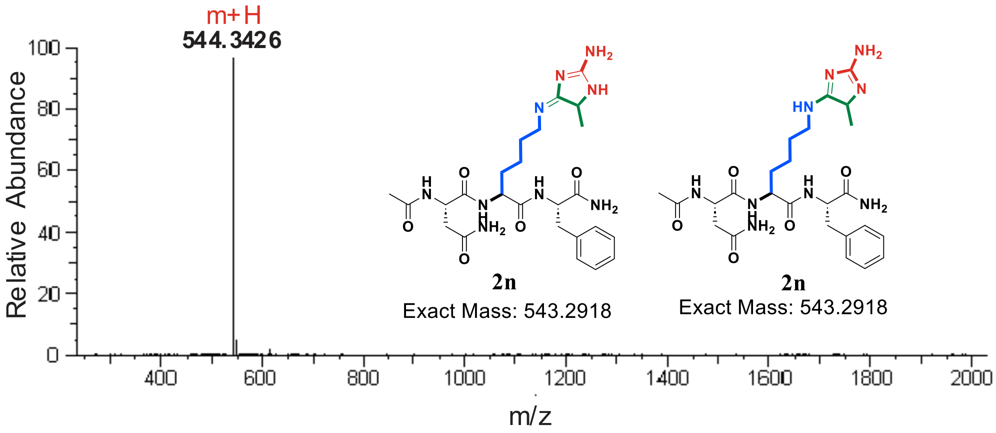

Supplementary Figure 6c: Intermolecular Labeling of Peptide 1o with Guanidine Hydrochloride

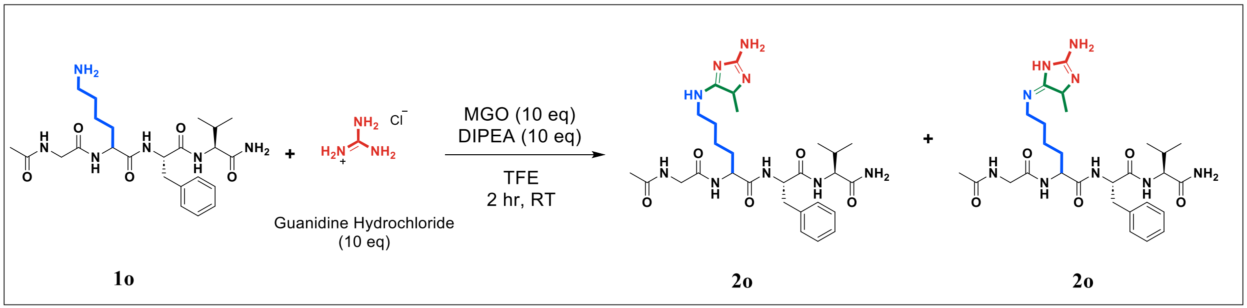

Linear peptide **1o** (1.0 mg, 0.002 mmol, 1.0 eq) was dissolved in 250 µL of TFE and the solution stirred at room temperature for 20 minutes with DIPEA (10 eq) and methylglyoxal (10 eq). Then, guanidine hydrochloride (10 eq) was added, and the reaction mixture was left to stir at room temperature for 2 hours. Samples were taken from the reaction mixture and injected into the HPLC using **HPLC Method A** to determine % conversion. The masses of the products were confirmed with LC-MS and compiled below. The conversion of the labeled product was determined to be (>98%).

Ac-GKFV-CONH2 ($C_{24}H_{38}N_6O_5$) linear peptide 1o. LCMS: *m/z* 491.2751 (calcd $[M+H]^+$ = 491.2976) (HPLC analysis at 220 nm). Retention time in HPLC: 7.1 min

Ac-GKFV-CONH2 ($C_{28}H_{43}N_9O_5$) labeled products 2o. LCMS: 586.5103 (calcd $[M+H]^+$ = 586.3460) (HPLC analysis at 220 nm). Retention time in HPLC: 12.3 min. (>98%)

HPLC of Ac-GKFV-CONH2 Starting Peptide at 220nm

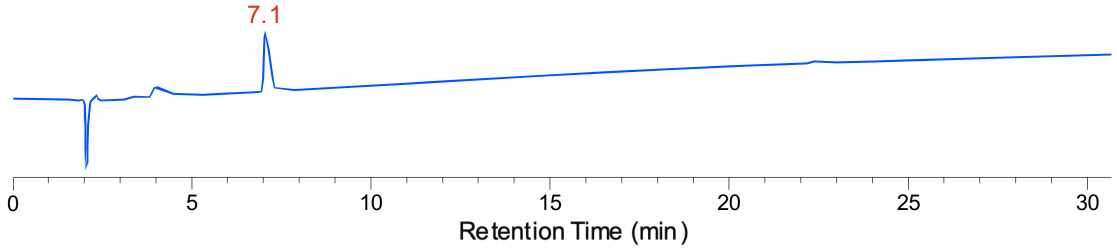

HRMS Trace of Peak at 7.1 min

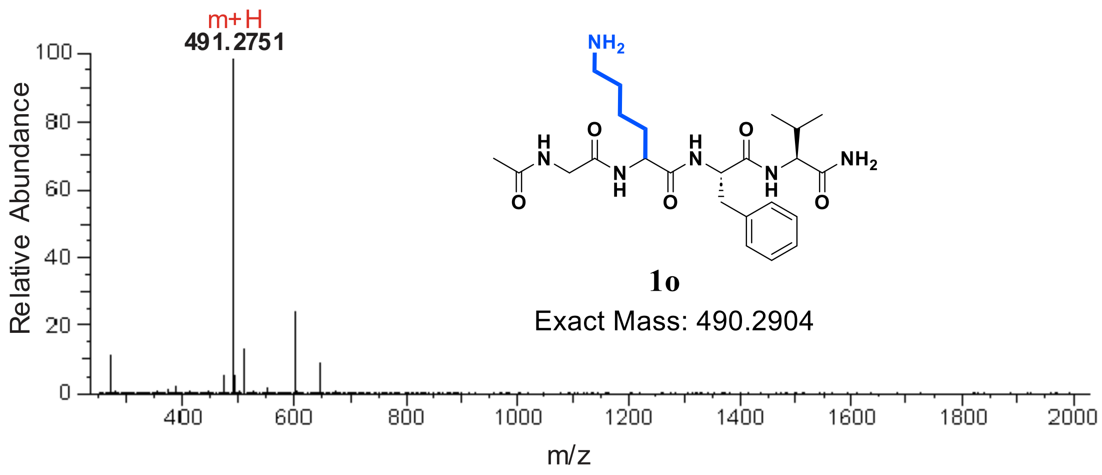

HPLC Trace of Ac-GKFV-CONH₂ Reaction Mixture at 220nm

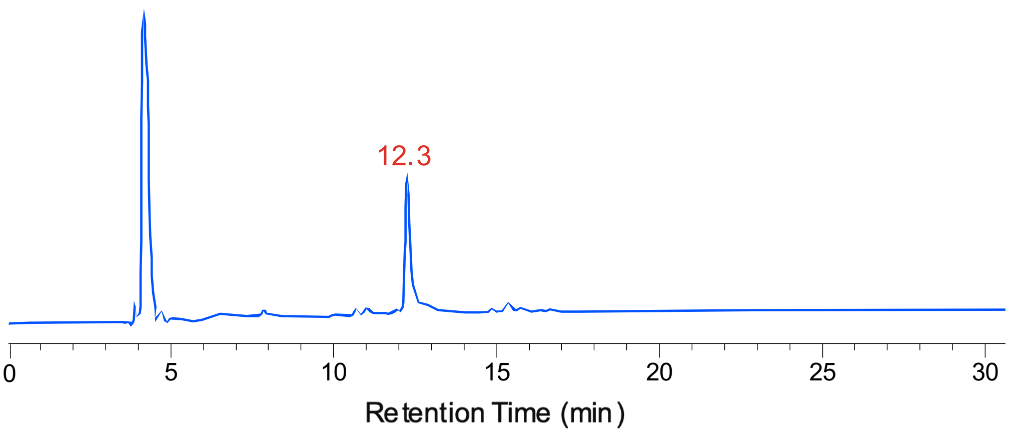

HRMS Trace of Peak at 12.3 min

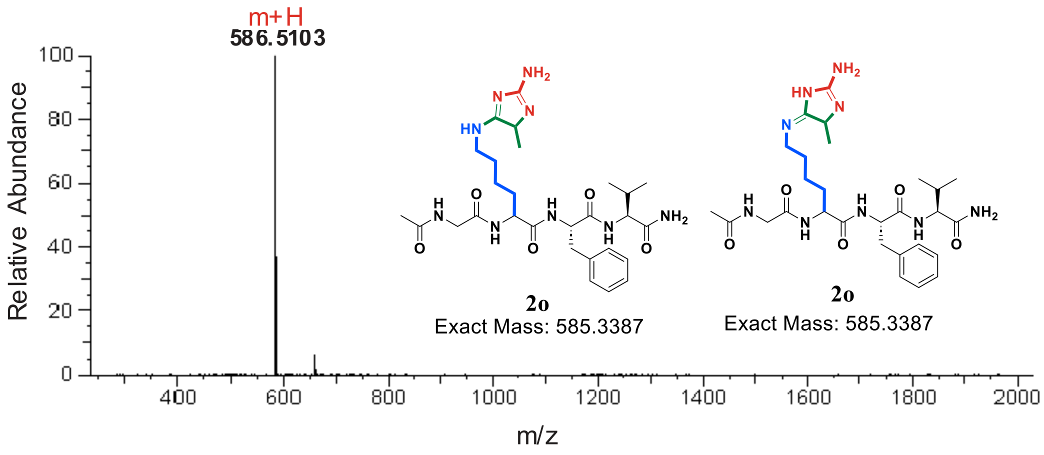

Supplementary Figure 6d: Intermolecular Labeling of Peptide 1p with Guanidine Hydrochloride

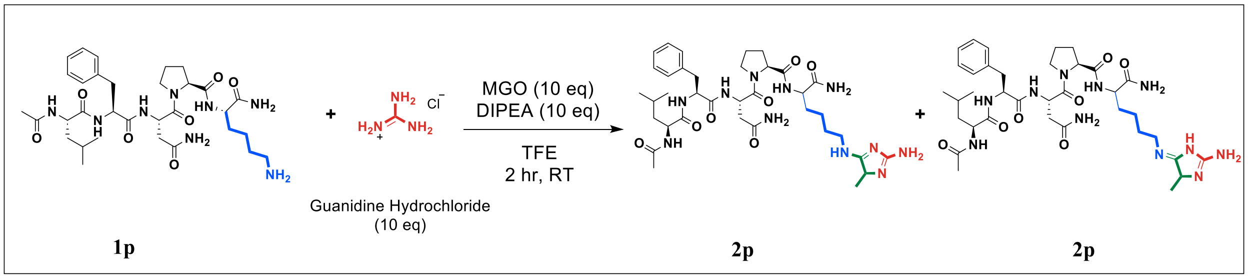

Linear peptide **1p** (1.0 mg, 0.002 mmol, 1.0 eq) was dissolved in 250 µL of TFE and the solution stirred at room temperature for 20 minutes with DIPEA (10 eq) and methylglyoxal (10 eq). Then, guanidine hydrochloride (10 eq) was added, and the reaction mixture was left to stir at room temperature for 2 hours. Samples were taken from the reaction mixture and injected into the HPLC using **HPLC Method A** to determine % conversion. The masses of the products were confirmed with LC-MS and compiled below. The conversion of the labeled product was determined to be (93%).

Ac-LFNPK-CONH2 ($C_{32}H_{50}N_8O_7$) linear peptide 1p. LCMS: *m/z* 659.3868 (calcd $[M+H]^+$ = 659.3875) (HPLC analysis at 220 nm). Retention time in HPLC: 10.2 min

Ac-LFNPK-CONH2 ($C_{36}H_{55}N_{11}O_7$) labeled products 2p. LCMS: 754.6190 (calcd $[M+H]^+$ = 754.4359) (HPLC analysis at 220 nm). Retention time in HPLC: 11.3 min. (93%)

HPLC of Ac-LFNPK-CONH2 Starting Peptide at 220 nm

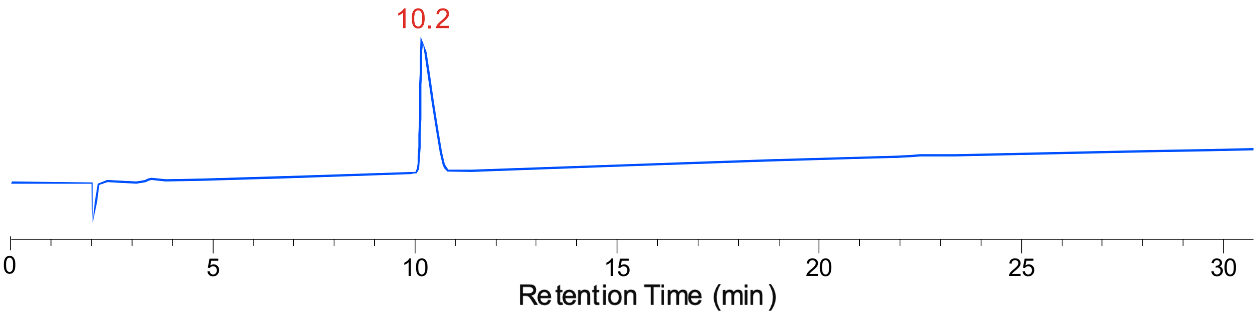

HRMS Trace of Peak at 10.2 min

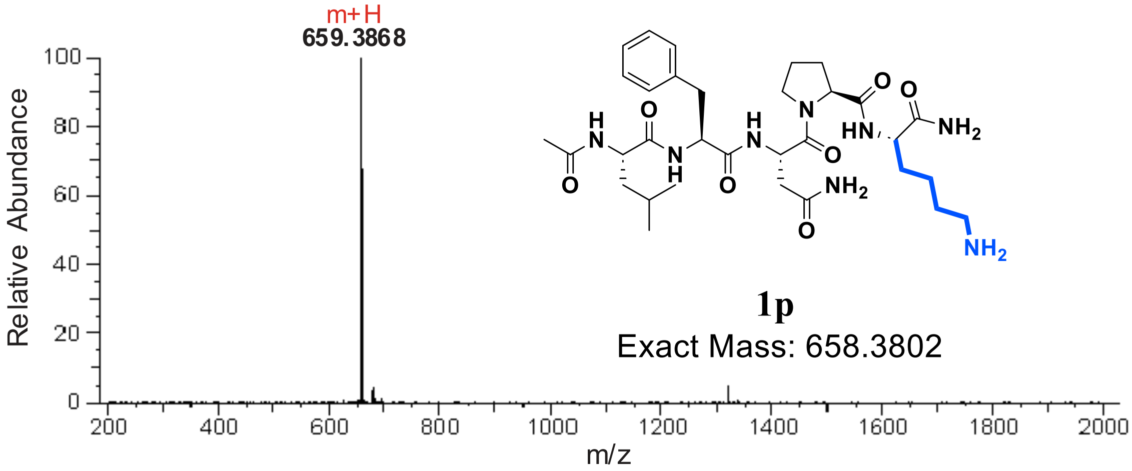

HPLC Trace of Ac-LFNPK-CONH$_2$ Reaction Mixture at 220 nm

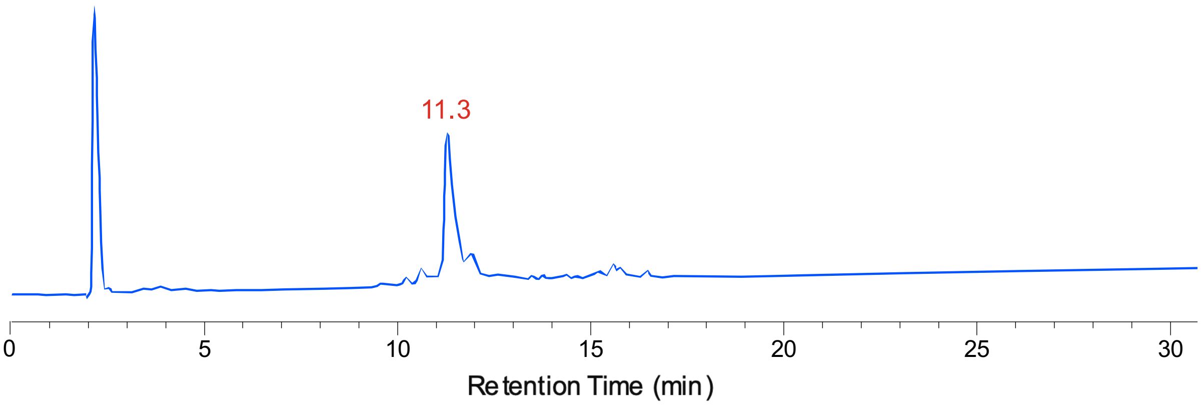

HRMS Trace of Peak at 11.3 min

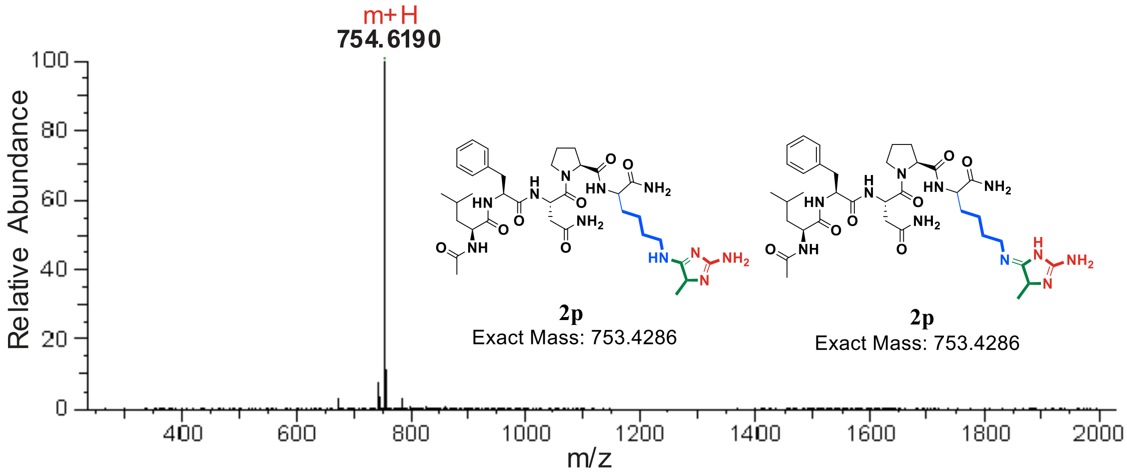

Supplementary Figure 6e: Intermolecular Labeling of Peptide 1p' with Guanidine Hydrochloride and Phenylglyoxal

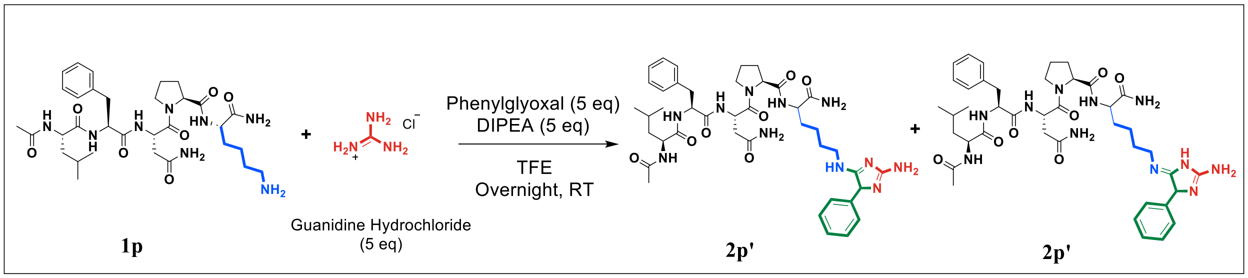

Linear peptide **1p** (1.0 mg, 0.002 mmol, 1.0 eq) was dissolved in 250 µL of TFE and the solution stirred at room temperature for 20 minutes with DIPEA (5 eq) and phenylglyoxal (5 eq). Then, guanidine hydrochloride (5 eq) was added, and the reaction mixture was left to stir at room temperature overnight. Samples were taken from the reaction mixture and injected into the HPLC using **HPLC Method A** to determine % conversion. The masses of the products were confirmed with LC-MS and compiled below. The conversion of the labeled product was determined to be (45%).

Ac-LFNPK-CONH$_2$ ($C_{32}H_{50}N_8O_7$) linear peptide 1p. LCMS: *m/z* 659.3868 (calcd $[M+H]^+$ = 659.3875) (HPLC analysis at 220 nm). Retention time in HPLC: 10.2 min

Reaction Mixture

Ac-LFNPK-CONH$_2$ ($C_{32}H_{50}N_8O_7$) linear peptide 1p. LCMS: *m/z* 659.3881 (calcd $[M+H]^+$ = 659.3875) (HPLC analysis at 220 nm). Retention time in HPLC: 10.2 min. (55 %)

Ac-LFNPK-CONH$_2$ ($C_{41}H_{57}N_{11}O_7$) labeled products 2p'. LCMS: 816.4509 (calcd $[M+H]^+$ = 816.4515) (HPLC analysis at 220 nm). Retention time in HPLC: 10.7 min. (45%)

HPLC of Ac-LFNPK-CONH$_2$ Starting Peptide at 220 nm

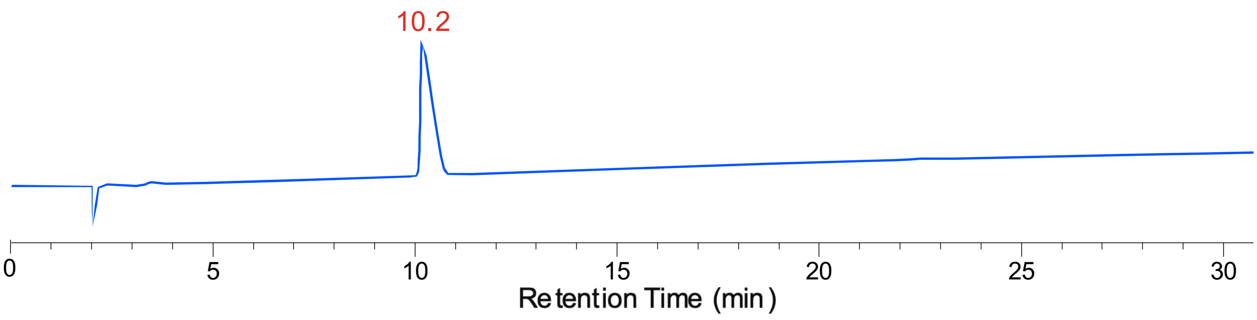

HRMS Trace of Peak at 10.2 min

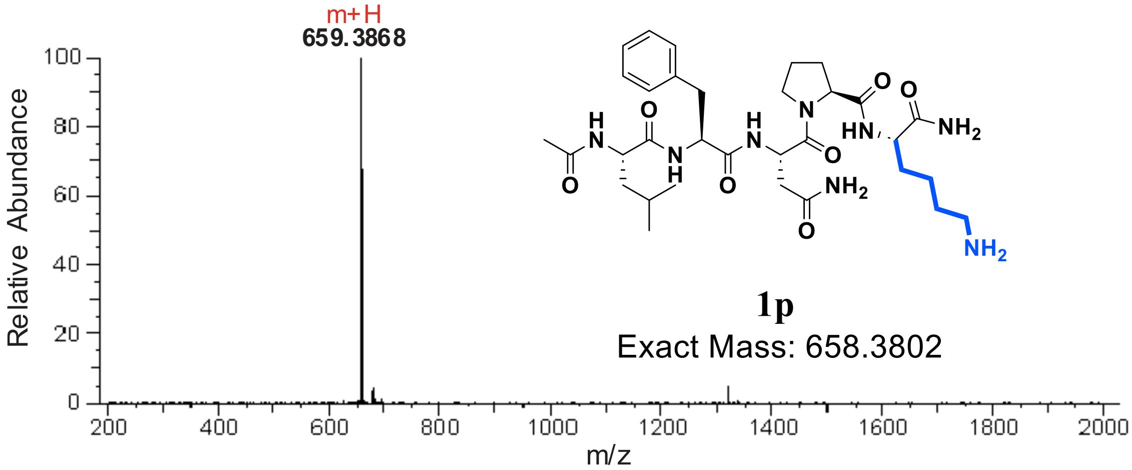

HPLC Trace of Ac-LFNPK-CONH₂ Reaction Mixture at 220 nm

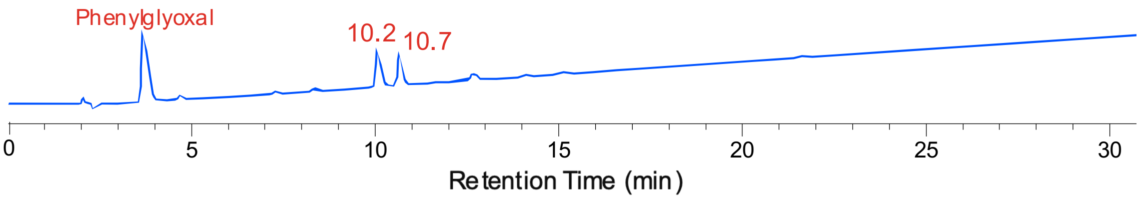

HRMS Trace of Peak at 10.2 min

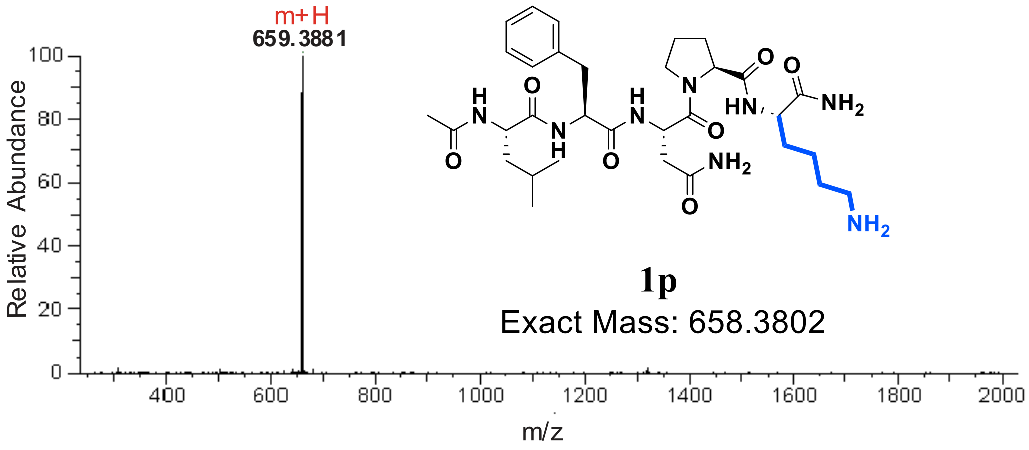

HRMS Trace of Peak at 10.7 min

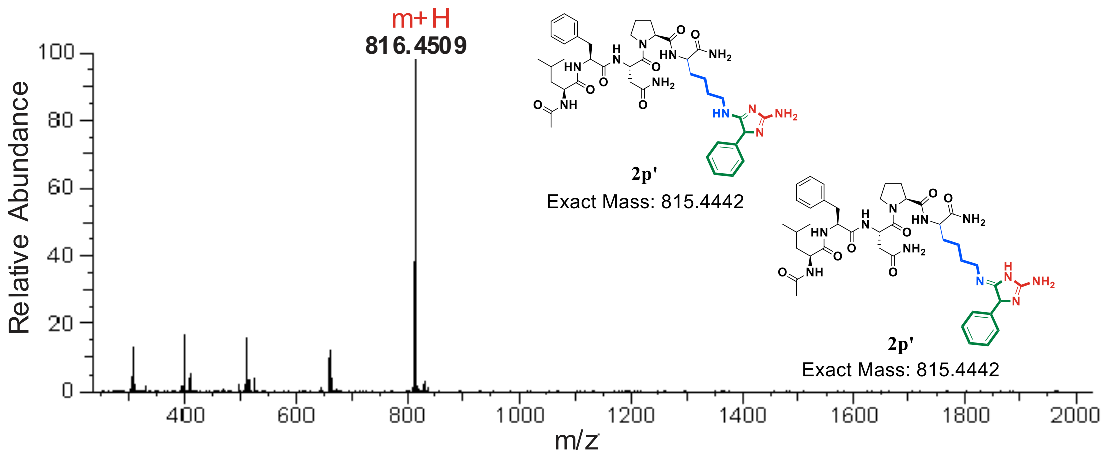

Supplementary Figure 6f: Intermolecular Labeling of Peptide 1q with Guanidine Hydrochloride

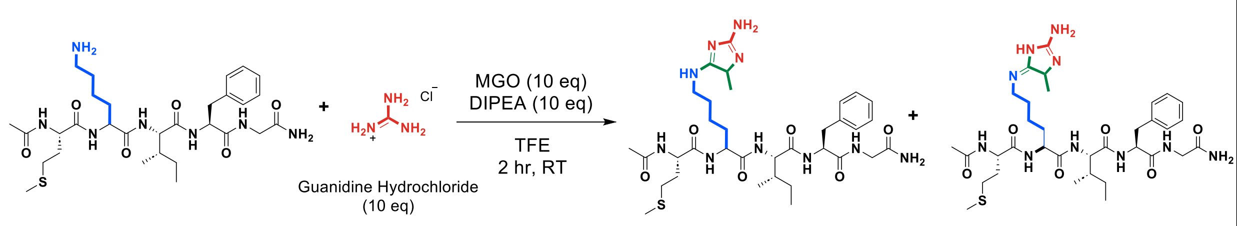

Linear peptide **1q** (1.0 mg, 0.002 mmol, 1.0 eq) was dissolved in 250 µL of TFE and the solution stirred at room temperature for 20 minutes with DIPEA (10 eq) and methylglyoxal (10 eq). Then, guanidine hydrochloride (10 eq) was added, and the reaction mixture was left to stir at room temperature for 2 hours. Samples were taken from the reaction mixture and injected into the HPLC using **HPLC Method A** to determine % conversion. The masses of the products were confirmed with LC-MS and compiled below. The conversion of the labeled product was determined to be (90%).

Ac-MKIFG-CONH2 ($C_{30}H_{49}N_7O_6S$) linear peptide 1q. LCMS: *m/z* 636.3527 (calcd $[M+H]^+$ = 636.3538) (HPLC analysis at 220 nm). Retention time in HPLC: 11.2 min

Reaction Mixture:

Ac-MKIFG-CONH2 ($C_{30}H_{49}N_7O_6S$) linear peptide 1q. LCMS: *m/z* 636.4589 (calcd $[M+H]^+$ = 636.3538) (HPLC analysis at 220 nm). Retention time in HPLC: 11.8 min. (10%)

Ac-MKIFG-CONH2 ($C_{34}H_{54}N_{10}O_6S$) labeled products 2q. LCMS: *m/z* 731.5867 (calcd $[M+H]^+$ = 731.4021) (HPLC analysis at 220 nm). Retention time in HPLC: 12.4 min. (90%)

HPLC of Ac-MKIFG-CONH₂ Starting Peptide at 220 nm

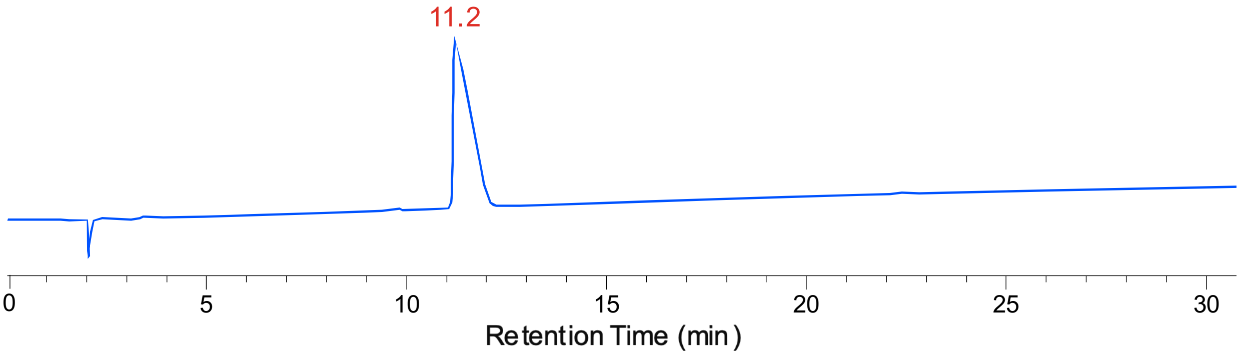

HRMS Trace of Peak at 11.2 min

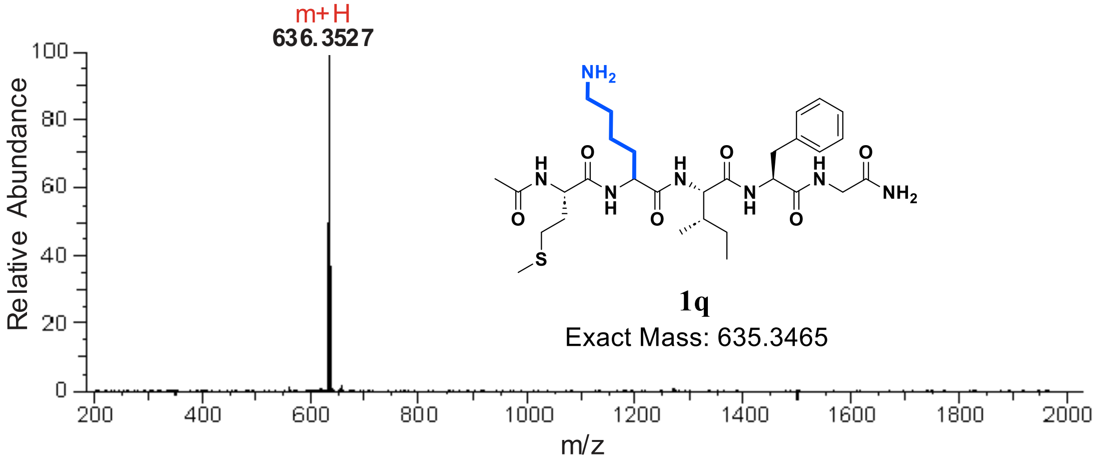

HPLC Trace of Ac-MKIFG-CONH₂ Reaction Mixture at 220 nm

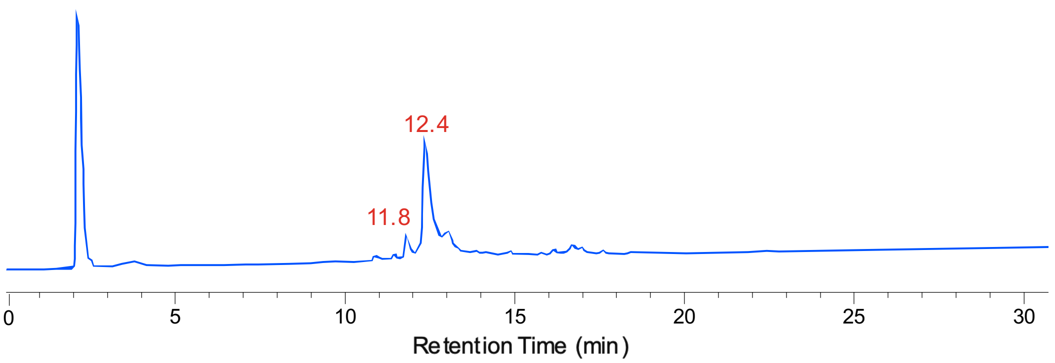

HRMS Trace of Peak at 11.8 min

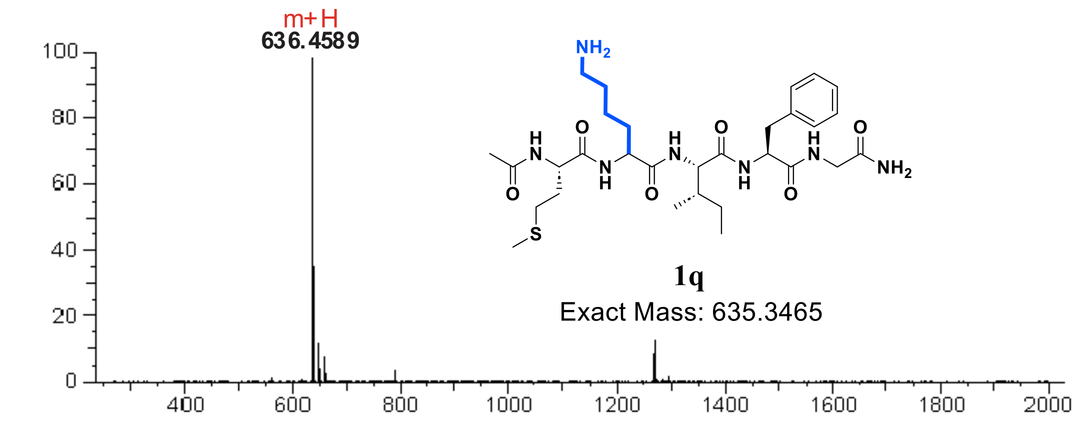

HRMS Trace of Peak at 12.4 min

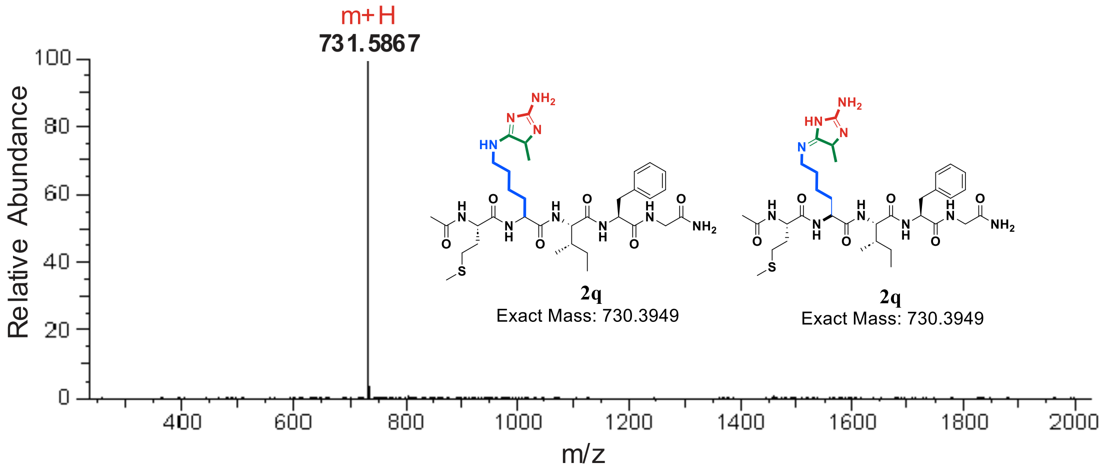

Supplementary Figure 6g: Intermolecular Labeling of Peptide 1r with Guanidine Hydrochloride

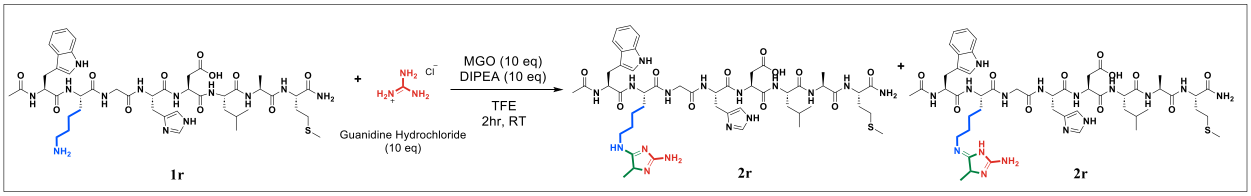

Linear peptide **1r** (1.0 mg, 0.002 mmol, 1.0 eq) was dissolved in 250 µL of TFE and the solution stirred at room temperature for 20 minutes with DIPEA (10 eq) and methylglyoxal (10 eq). Then, guanidine hydrochloride (10 eq) was added, and the reaction mixture was left to stir at room temperature for 2 hours. Samples were taken from the reaction mixture and injected into the HPLC using **HPLC Method A** to determine % conversion. The masses of the products were confirmed with LC-MS and compiled below. The conversion of the labeled product was determined to be (70%).

Ac-WKGHDLAM-CONH2 ($C_{45}H_{67}N_{13}O_{11}S$) linear peptide 1r. LCMS: *m/z* 998.6251 (calcd $[M+H]^+$ = 998.4876) *m/z* 499.9830 (calcd $[M+2H/2]^+$ = 499.4876) (HPLC analysis at 220 nm). Retention time in HPLC: 7.8 min

Reaction Mixture:

Ac-WKGHDLAM-CONH2 ($C_{45}H_{67}N_{13}O_{11}S$) linear peptide 1r. LCMS: *m/z* 998.5234 (calcd $[M+H]^+$ = 998.4876), *m/z* 500.0280 (calcd $[M+2H/2]^+$ = 499.4876) (HPLC analysis at 220 nm). Retention time in HPLC: 8.0 min. (30%)

Ac-WKGHDLAM-CONH2 ($C_{49}H_{72}N_{16}O_{11}S$) labeled products 2r. LCMS: *m/z* 1093.6323 (calcd $[M+H]^+$ = 1093.5360), *m/z* 547.6424 (calcd $[M+2H/2]^+$ = 547.2722) (HPLC analysis at 220 nm). Retention time in HPLC: 8.6 min. (70%)

HPLC Trace of Ac-WKGHDLAM-CONH2 Starting Peptide at 220 nm

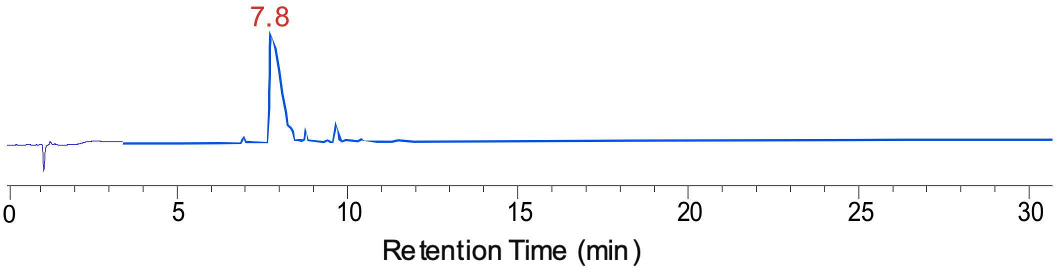

HRMS Trace of Peak at 7.8 min

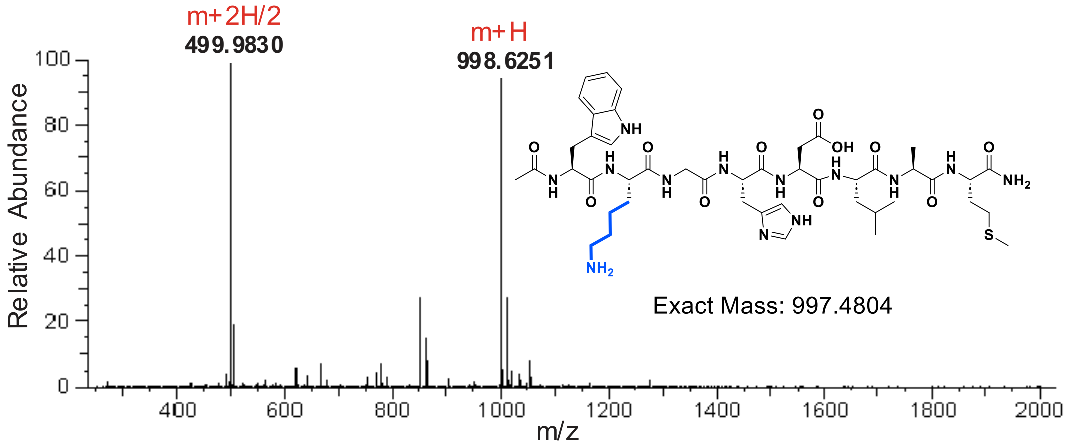

HPLC Trace of Ac-WKGHDLAM-CONH$_2$ Reaction Mixture at 220 nm

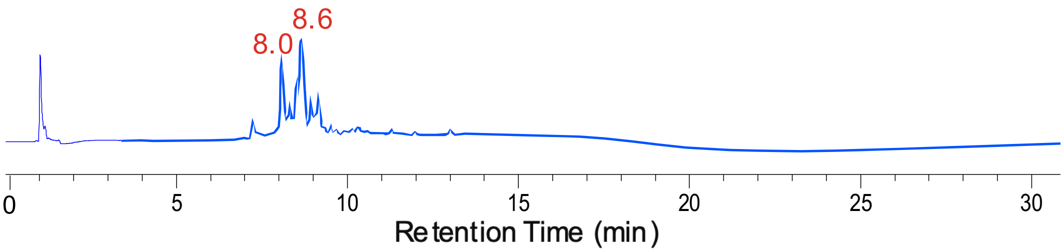

HRMS Trace of Peak at 8.0 min

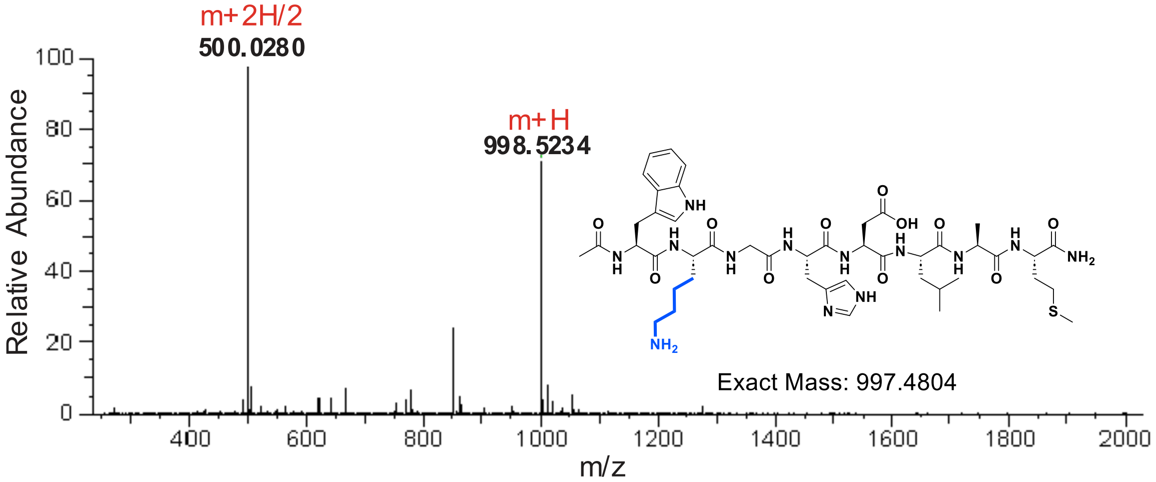

HRMS Trace of Peak at 8.6 min

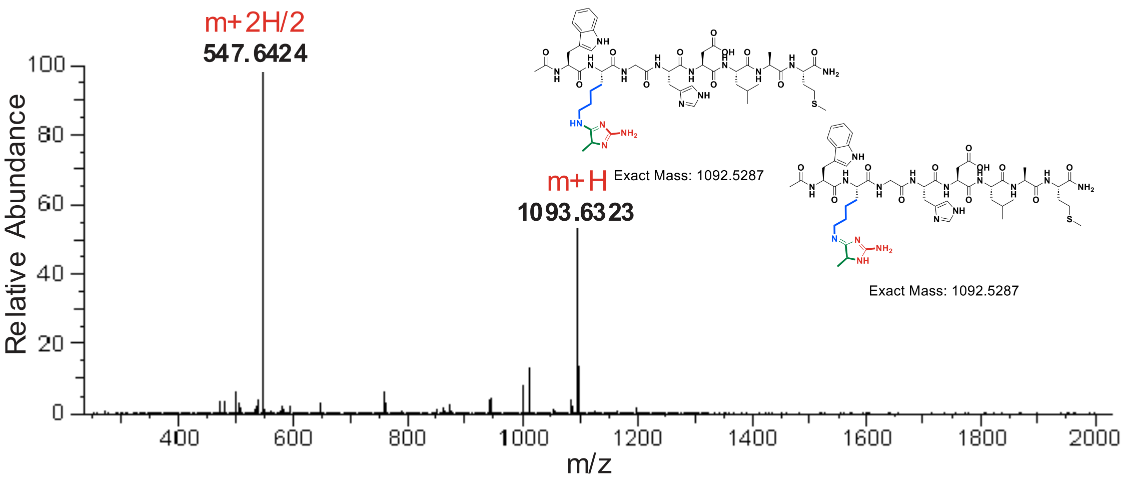

Supplementary Figure 6h: Intermolecular Labeling of Peptide 1r' with Guanidine Hydrochloride

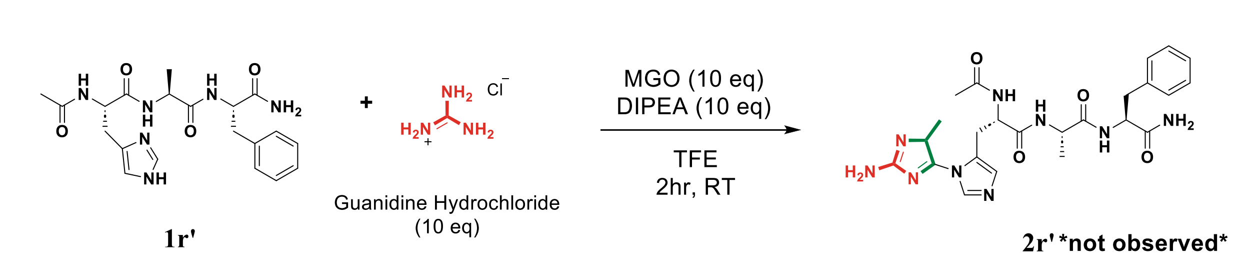

Linear peptide **1r'** (1.0 mg, 0.002 mmol, 1.0 eq) was dissolved in 250 µL of TFE and the solution stirred at room temperature for 20 minutes with DIPEA (10 eq) and methylglyoxal (10 eq). Then, guanidine hydrochloride (10 eq) was added, and the reaction mixture was left to stir at room temperature for 2 hours. Samples were taken from the reaction mixture and injected into the HPLC using **HPLC Method A** to determine % conversion. The masses of the products were confirmed with LC-MS and compiled below. The conversion of the labeled product was determined to be (0%).

Ac-HAF-CONH$_2$ ($C_{20}H_{26}N_6O_4$) linear peptide 1r. LCMS: *m/z* 415.2086 (calcd $[M+H]^+$ = 415.2088) (HPLC analysis at 220 nm). Retention time in HPLC: 6.1 min

Reaction Mixture:

Ac-HAF-CONH$_2$ ($C_{20}H_{26}N_6O_4$) linear peptide 1r. LCMS: *m/z* 415.2087 (calcd $[M+H]^+$ = 415.2088) (HPLC analysis at 220 nm). Retention time in HPLC: 5.8 min. (>99%)

HPLC Trace of Ac-HAF-CONH$_2$ Starting Peptide at 220 nm

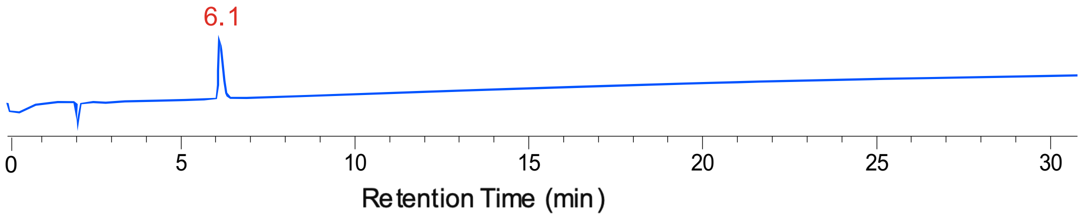

HRMS Trace of Peak at 6.1 min

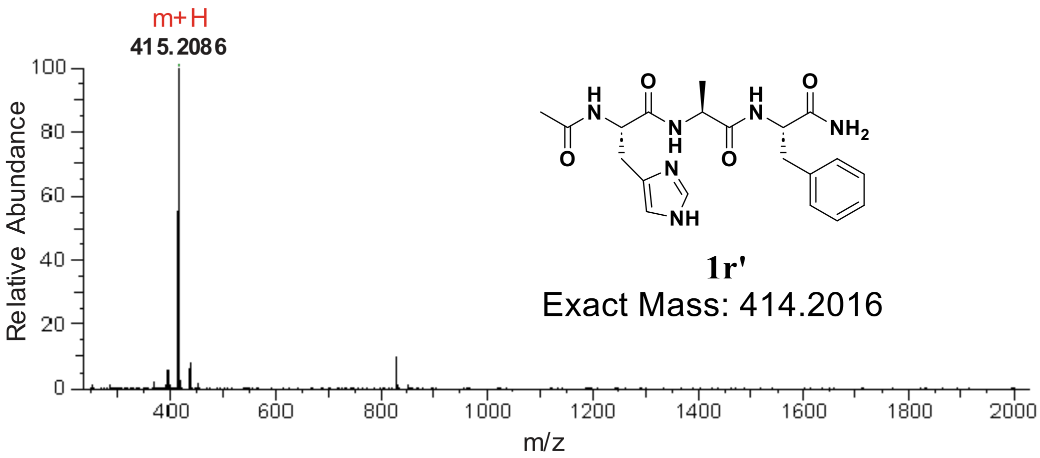

HPLC Trace of Ac-HAF-CONH$_2$ Reaction Mixture Starting Peptide at 220 nm

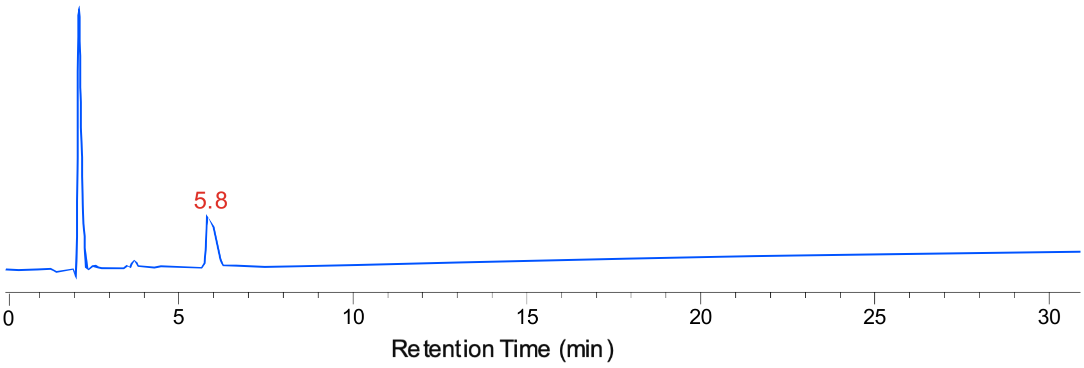

HRMS Trace of Peak at 5.8 min

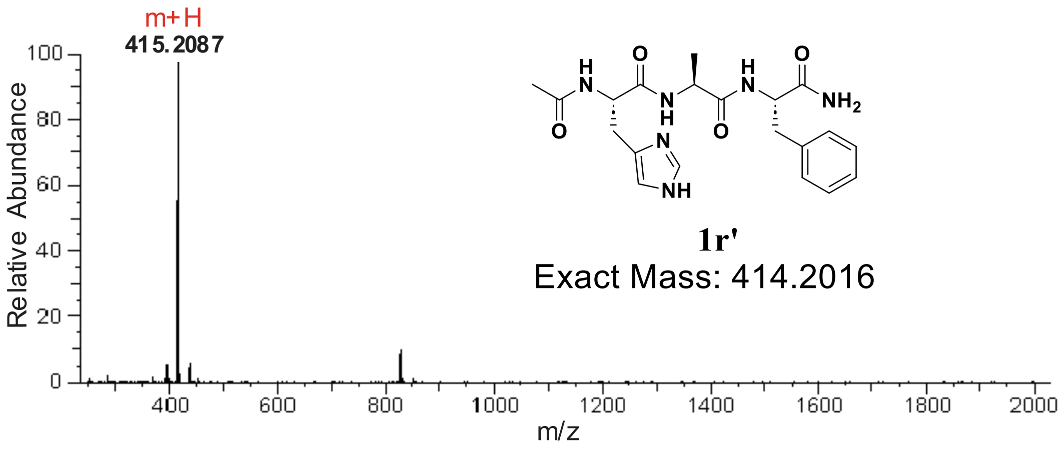

Supplementary Figure 7: Cyclization of Peptide 1s in TFE

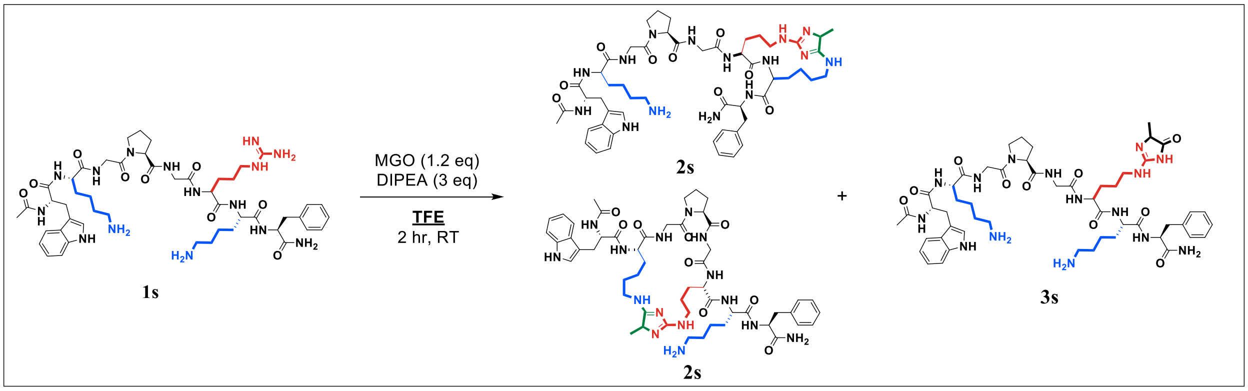

Linear peptide **1s** (1.0 mg, 0.001 mmol, 1.0 eq) was dissolved in 250 µL of TFE and the solution was left to stir at room temperature for 5 minutes with DIPEA (3 eq). Methylglyoxal (1.2 eq) was added to the reaction mixture and was left to stir at room temperature for 2 hours. Samples were taken from the reaction mixture and injected into the HPLC using **HPLC Method A** to determine % conversion. The masses of the products were confirmed with LC-MS and compiled below. The conversion of the cyclized products was determined to be (83%).

Ac-WKGPGRKF-CONH2 ($C_{49}H_{73}N_{15}O_9$) linear peptide 1s. LCMS: *m/z* 1016.6824 (calcd $[M+H]^+$ = 1016.5788) (HPLC analysis at 220 nm). Retention time in HPLC: 8.4 min

Reaction Mixture:

Ac-WKGPGRKF-CONH2 ($C_{49}H_{73}N_{15}O_9$) linear peptide 1s. LCMS: *m/z* 1016.6629 (calcd $[M+H]^+$ = 1016.5788), *m/z* 509.0260 (calcd $[M+2H/2]^+$ = 508.7936) (HPLC analysis at 220 nm). Retention time in HPLC: 9.0 min (8%)

Ac-WKGPGRKF-CONH2 ($C_{52}H_{75}N_{15}O_{10}$) arginine adduct products 3s. LCMS: 1070.6469 (calcd $[M+H]^+$ = 1070.5894), *m/z* 536.0228 (calcd $[M+2H/2]^+$ = 535.7989) (HPLC analysis at 220 nm). Retention time in HPLC: 9.3 min (9%)

Ac-WKGPGRKF-CONH2 ($C_{52}H_{73}N_{15}O_9$) cyclized peptide products 2s. LCMS: *m/z* 1052.6835 (calcd $[M+H]^+$ = 1052.5788), 527.1143 (calcd $[M+2H/2]^+$ = 526.7936) (HPLC analysis at 220 nm). Retention time in HPLC: 11.6 min (83%)

HPLC Trace of Ac-WKGPGRKF-CONH$_2$ Starting Peptide at 220 nm

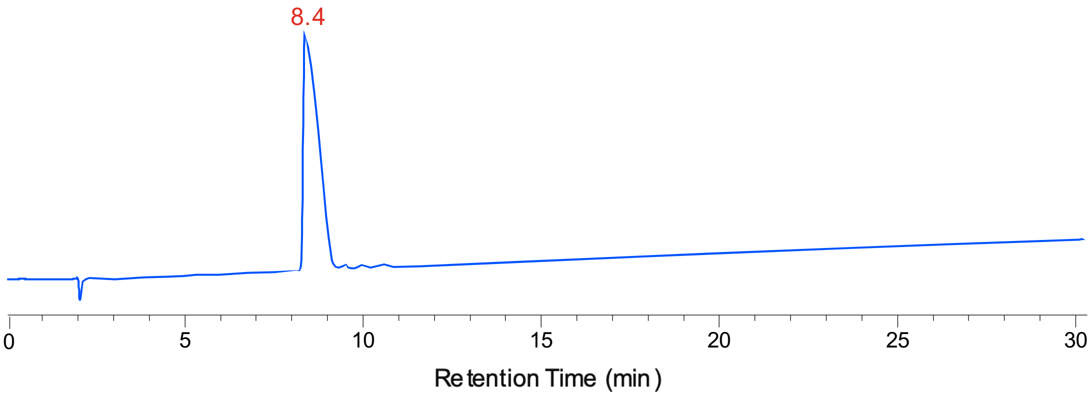

HRMS Trace of Peak at 8.4 min

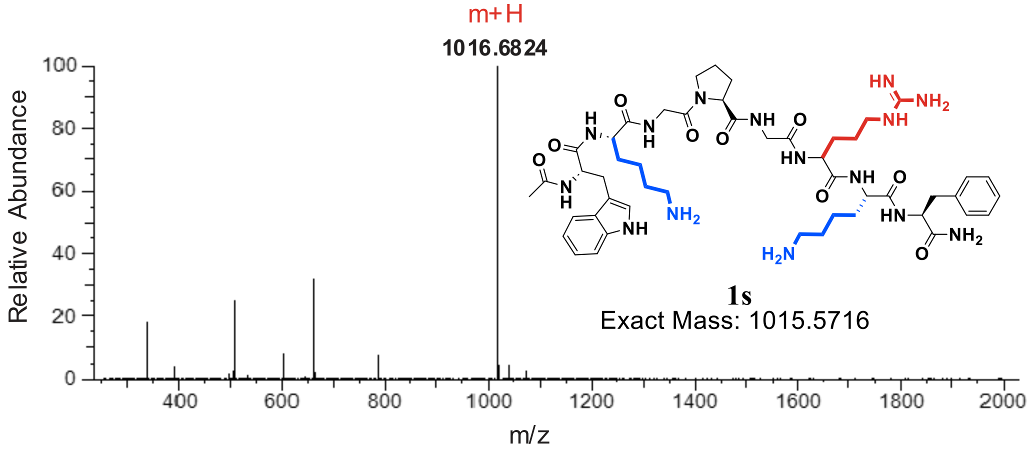

HPLC Trace of Ac-WKGPGRKF-CONH$_2$ Reaction Mixture at 220 nm

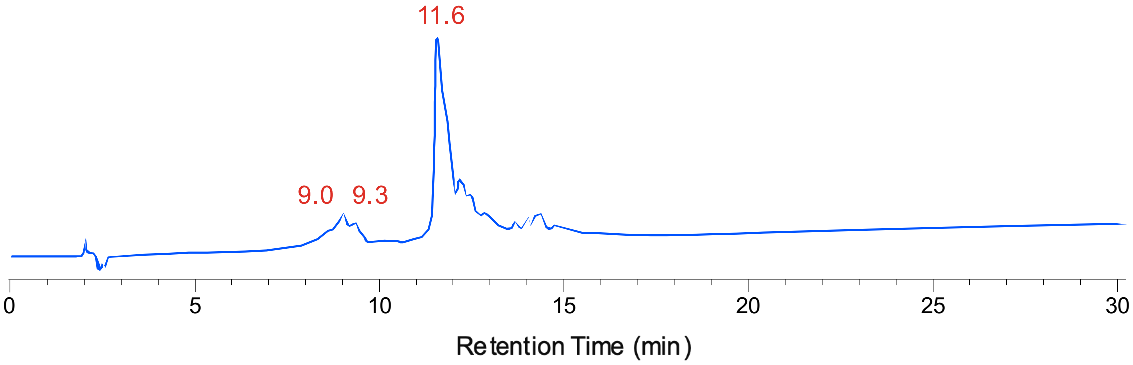

HRMS Trace of Peak at 9.0 min

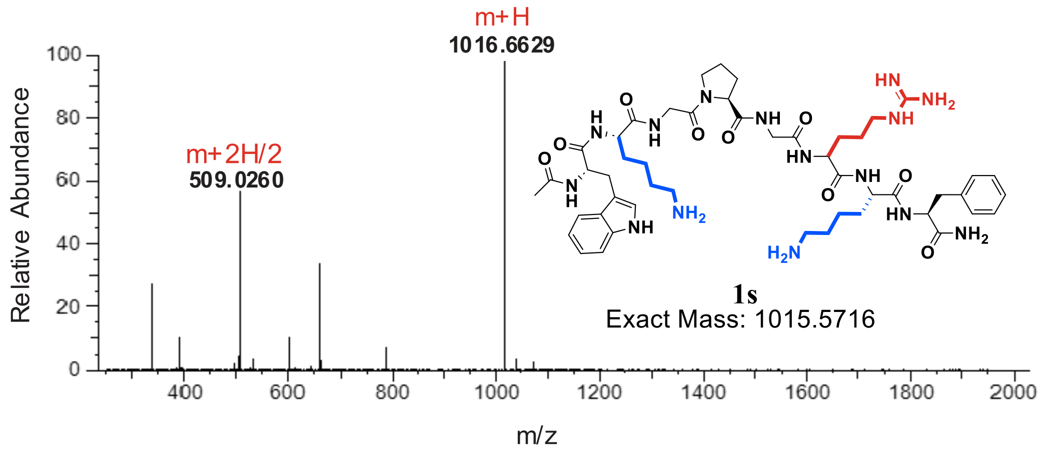

HRMS Trace of Peak at 9.3 min

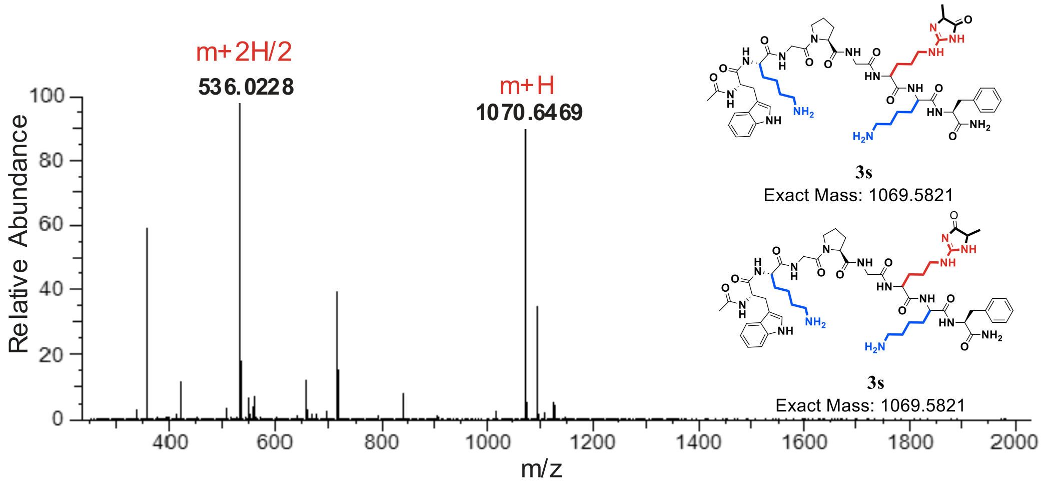

HRMS Trace of Peak at 11.6 min

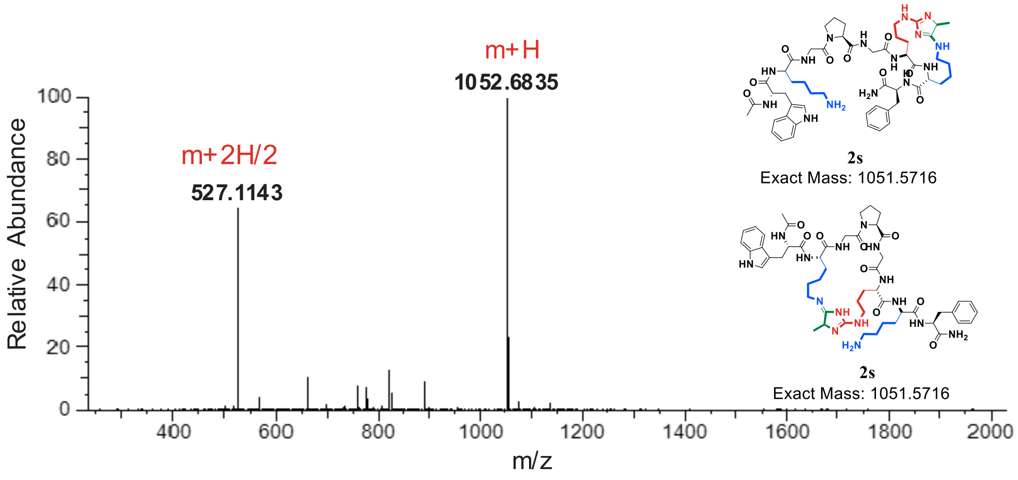

Supplementary Figure 8. Cyclization of Peptide 2s in HFIP[2]

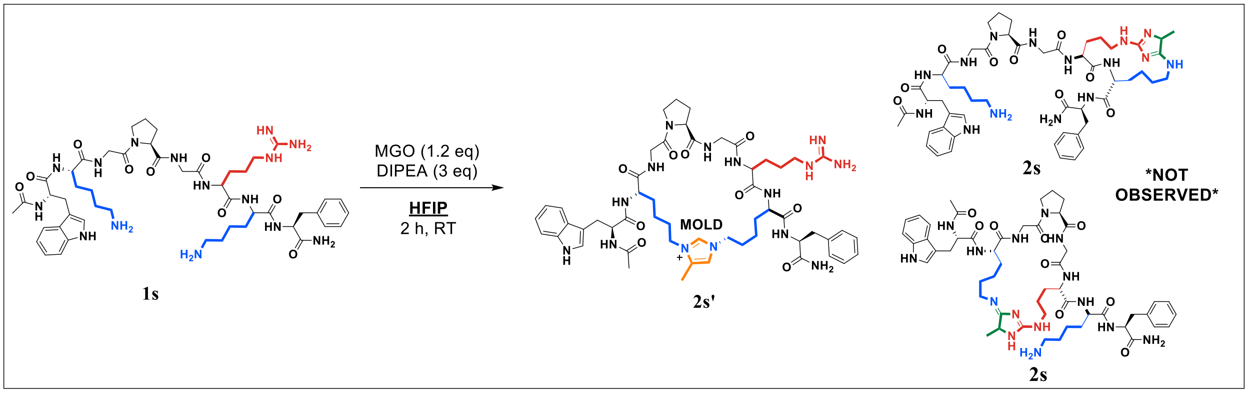

Linear peptide **1s** (1.0 mg, 0.001 mmol, 1.0 eq) was dissolved in 250 µL of HFIP and the solution was left to stir at room temperature for 5 minutes with DIPEA (3 eq). Methylglyoxal (1.2 eq) was added to the reaction mixture and was left to stir at room temperature for 2 hours. Samples were taken from the reaction mixture and injected into the HPLC using **HPLC Method A** to determine % conversion. The masses of the products were confirmed with LC-MS and compiled below. The formation of the cyclized product was not observed, the conversion of the Lys-Lys cyclized product was determined to be (76%).

Ac-WKGPGRKF-CONH$_2$ ($C_{49}H_{73}N_{15}O_9$) linear peptide 1s. LCMS: *m/z* 1016.6824 (calcd $[M+H]^+$ = 1016.5788) (HPLC analysis at 220 nm). Retention time in HPLC: 8.4 min

Reaction Mixture:

Ac-WKGPGRKF-CONH$_2$ ($C_{49}H_{73}N_{15}O_9$) linear peptide 1s. LCMS: *m/z* 1016.5789 (calcd $[M+H]^+$ = 1016.5788 (HPLC analysis at 220 nm). Retention time in HPLC: 8.8 min. (24%)

Ac-WKGPGRKF-CONH$_2$ ($C_{53}H_{74}N_{15}O_9^+$) MOLD crosslink product 2s'. LCMS: *m/z* 1064.5782 (calcd $[M+H]^+$ = 1064.5788), 532.7928 (calcd $[M+2H/2]^+$ = 532.2894) (HPLC analysis at 220 nm). Retention time in HPLC: 11.9 min. (76%)

HPLC Trace of Ac-WKGPGRKF-CONH$_2$ Starting Peptide at 220 nm

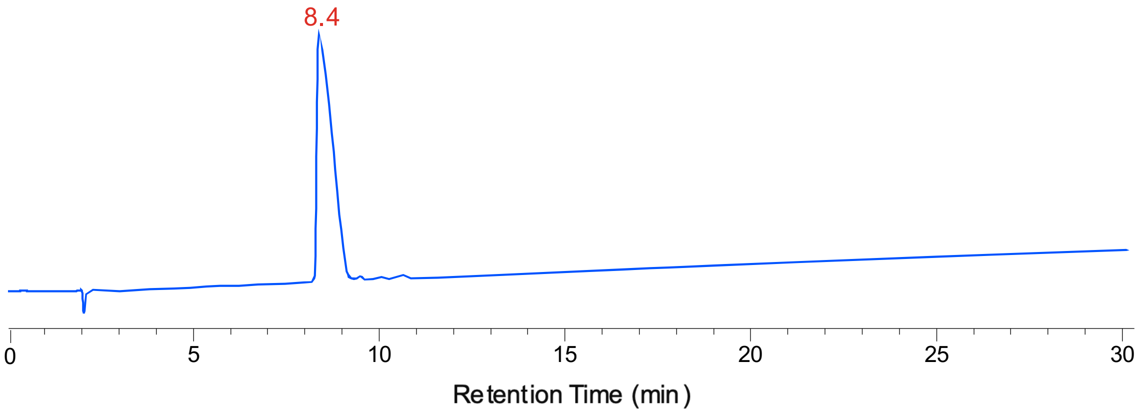

HRMS Trace of Peak at 8.4 min

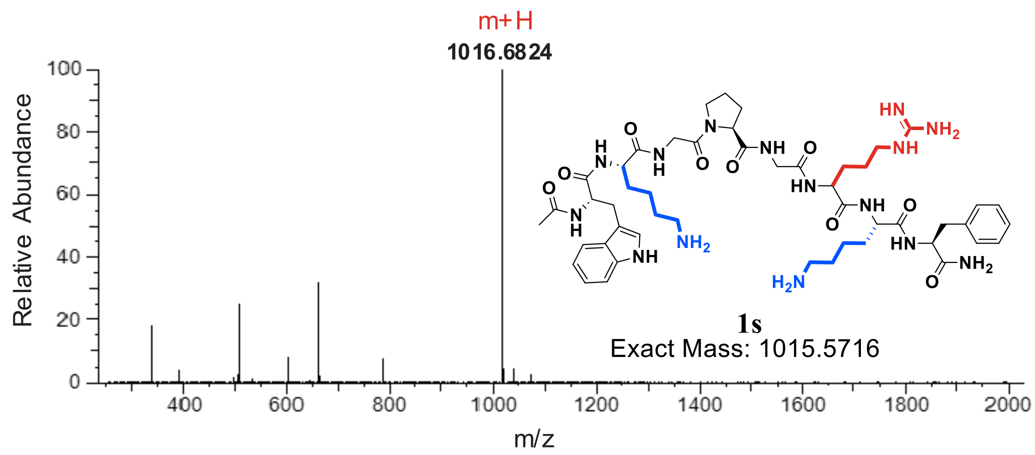

HPLC Trace of Ac-WKGPGRKF-CONH$_2$ Reaction Mixture in HFIP at 220 nm

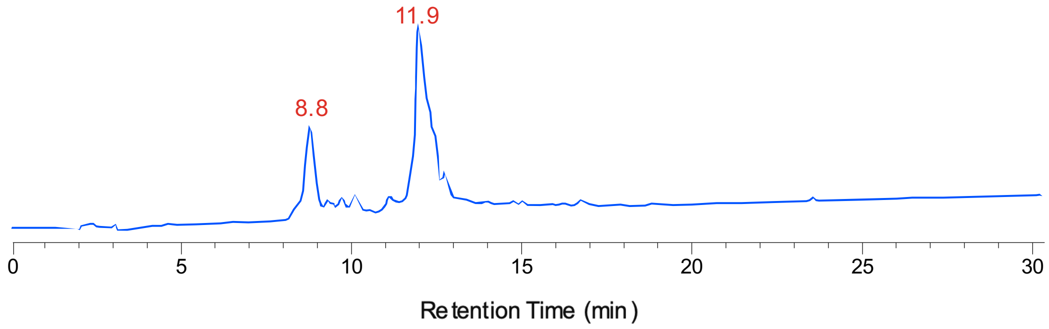

HRMS Trace of Peak at 8.8 min

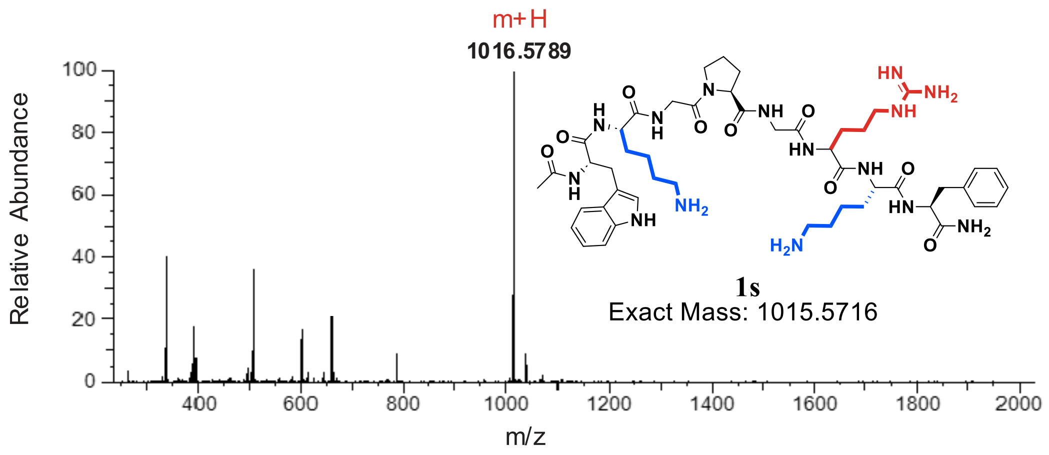

HRMS Trace of Peak at 11.9 min

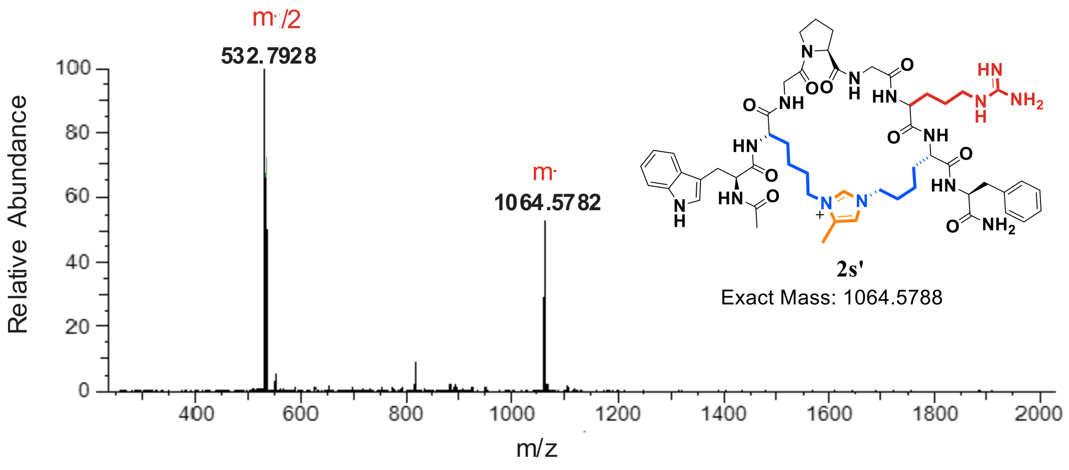

References

1. Chan, W. C.; White, P. D. Fmoc solid phase peptide synthesis: A practical approach (Oxford Univ. Press, New York, 2000).

2. Guo, P.; Chu, X.; Wu, C.; Qiao, T.; Guan, W.; Zhou, C.; Wang, T.; Tian, C.; He, G.; Chen, G. Peptide stapling by crosslinking two amines with a-ketoaldehydes through diverse modified glyoxal-lysine dimer linkers. *Angew. Chem. Int. Ed.* **2024**, *63*, 1-8.
